# Dynamic Cap‐Mediated Substrate Access and Potent Inhibitor Design of Monkeypox Virus I7L Protease

**DOI:** 10.1002/advs.202501625

**Published:** 2025-04-07

**Authors:** Haixia Su, Guoqing Wu, Muya Xiong, Yuhang Wang, Junyuan Cao, Mengyuan You, Yingchun Xiang, Tianqing Nie, Minjun Li, Gengfu Xiao, Leike Zhang, Qiang Shao, Yechun Xu

**Affiliations:** ^1^ State Key Laboratory of Drug Research, Shanghai Institute of Materia Medica Chinese Academy of Sciences Shanghai 201203 China; ^2^ University of Chinese Academy of Sciences Beijing 100049 China; ^3^ Lingang Laboratory Shanghai 200031 China; ^4^ School of Physical Science and Technology ShanghaiTech University Shanghai 201210 China; ^5^ School of Pharmaceutical Science and Technology, Hangzhou Institute for Advanced Study University of Chinese Academy of Sciences Hangzhou 310024 China; ^6^ School of Chinese Materia Medica Nanjing University of Chinese Medicine Nanjing 210023 China; ^7^ CAS Key Laboratory of Special Pathogens, Wuhan Institute of Virology, Center for Biosafety Mega‐Science Chinese Academy of Sciences Wuhan 430064 China; ^8^ Hubei Jiangxia Laboratory Wuhan 430200 China; ^9^ Shanghai Synchrotron Radiation Facility, Shanghai Advanced Research Institute Chinese Academy of Sciences Shanghai 201204 China

**Keywords:** covalent inhibitors, I7L protease, monkeypox virus, protein structures, substrate proteolysis

## Abstract

Monkeypox virus (MPXV), an orthopoxvirus that has long been endemic in Africa, has posed a significant global health threat since 2022. The I7L protease, a highly conserved cysteine proteinase essential for orthopoxvirus replication, represents a promising target for broad‐spectrum antiviral drug development. Here, the first crystal structure of MPXV I7L protease is reported, revealing its unique dimeric form and different conformations of a cap region nearby the active site. Molecular dynamics simulations and AlphaFold3 prediction of protease‐substrate structures both suggest that this highly flexible cap acts as a conformational switch, regulating the substrate access to the active site. Additionally, the structural basis of substrate recognition and the catalytic mechanism of the protease are elucidated, mapping determinants of substrate specificity. These insights enable us to design covalent inhibitors to mimic the natural substrates and develop a fluorescence resonance energy transfer (FRET)‐based protease assay to effectively assess the inhibitory activity, leading to the discovery of first‐in‐class inhibitors of MPXV I7L protease with nanomolar potency. Therefore, this work provides a comprehensive understanding of the MPXV I7L protease's structure, dynamics, and function, and presents an example of successful rational design of covalent peptidomimetic inhibitors, serving as a good starting point for drug development against MPXV.

## Graphical abstract

The first crystal structure of MPXV I7L protease, revealing its unique dimeric form is resolved. The structures and MD simulations uncovered a dynamic cap region that regulates substrate access to the active site. The structural basis of substrate recognition and the catalytic mechanism are elucidated, which led to the development of a FRET‐based assay for inhibitor screening and the design of potent inhibitors of MPXV I7L protease.

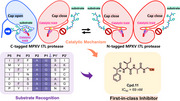

## 1 Introduction

Monkeypox virus (MPXV) is a double‐stranded DNA virus belonging to the *Orthopoxvirus* genus within the *Chordopoxvirinae* subfamily, alongside notable members such as variola (smallpox), vaccinia, cowpox, and ectromelia viruses.^[^ ^1^ ^]^ MPXV primarily causes zoonotic infections, characterized by symptoms such as vesiculopapular rashes, fever, and lymphadenopathy.^[^ ^2^ ^]^ First identified in humans in 1970 in the Democratic Republic of the Congo, MPXV has remained endemic in West and Central Africa. However, since 2022, an unprecedented global surge in cases has captured widespread attention. Between January 2022 and December 2024, over 100,000 laboratory‐confirmed cases of MPXV, including more than 250 deaths, have been reported across 126 countries, prompting the World Health Organization (WHO) to declare a Public Health Emergency of International Concern twice, in May 2022 and again in August 2024. Genomic studies have revealed two primary MPXV clades, clade I, which includes subclades 1a and 1b, and clade II, which includes subclades IIa and IIb.^[^ ^2^ ^]^ Clade I is associated with a higher virulence and mortality rate, ranging from 4% to 11%, whereas clade II generally presents with milder symptoms and a lower mortality rate of less than 4%.^[^ ^3^ ^]^ The recent global spread of clade I, which is characterized by enhanced human‐to‐human transmissibility, has raised significant concern.^[^ ^3^ ^]^ Although antiviral drugs, such as tecovirimat, brincidofovir, and cidofovir, have been approved for use against orthopoxviruses,^[^ ^4^ ^]^ clinical data regarding their efficacy specifically against MPXV remains limited.

The lifecycle of orthopoxviruses such as MPXV involves several key stages, including host cell entry through membrane fusion or macropinocytosis, uncoating, viral DNA transcription and replication, viral particle assembly, morphogenesis, intracellular transport, envelopment, and the release of mature virions.^[^ ^4^ ^]^ Among the viral proteins essential to this lifecycle, I7L, a 423‐residue cysteine protease, is highly conserved among orthopoxviruses and plays a critical role in viral maturation by cleaving core protein precursors at the AG↑X motif.^[^ ^5^, ^6^, ^7^ ^]^ Disruption of the I7L protease's hydrolytic activity effectively blocks virion morphogenesis, thereby preventing the production of mature viral particles and inhibiting viral replication.^[^ ^5^, ^8^ ^]^ Given the essential role of the proteases in the viral lifecycle as well as pathogenesis, they have been extensively targeted for antiviral drug development. This has been demonstrated by the successful inhibition of human immunodeficiency virus type 1 (HIV‐1) protease, hepatitis C virus (HCV) NS3/4A protease, and SARS‐CoV‐2 3C‐like protease (3CL^pro^).^[^ ^9^, ^10^, ^11^, ^12^, ^13^, ^14^, ^15^, ^16^ ^]^ The therapeutic benefits of protease inhibitors in a variety of infectious diseases highlight the potential of targeting I7L protease to develop anti‐MPXV drugs.

Despite its functional parallels with other viral proteases, I7L protease exhibits distinct structural and mechanistic features. Unlike HIV‐1 protease, an aspartic protease that utilizes a catalytic aspartyl dyad (Asp25/Asp25′) and accommodates a broad range of substrates,^[^ ^17^ ^]^ or HCV NS3/4A, a serine protease that relies on a His57‐Asp81‐Ser139 catalytic triad stabilized by a zinc cofactor,^[^ ^18^ ^]^ I7L protease belongs to the CE clan of cysteine proteases and employs a His241‐Asp258‐Cys328 catalytic triad. This configuration is also distinct from SARS‐CoV‐2 3CL^pro^, which works through a His41‐Cys145 dyad.^[^ ^19^ ^]^ Furthermore, I7L protease exhibits a unique substrate specificity, strictly recognizing glycine at the P1 position, whereas SARS‐CoV‐2 3CL^pro^ significantly prefers glutamine at this position.^[^ ^19^ ^]^ These differences influence both the enzymatic function and inhibitor design. Therefore, a comprehensive structural and functional characterization of I7L protease is crucial for the rational design and development of MPXV‐specific protease inhibitors.

In the present study, we report the first crystal structure of MPXV I7L protease, demonstrating a unique dimeric conformation. A detailed insight into the structure revealed a cap region nearby the active site that adopts two distinct states, termed “cap‐open” and “cap‐closed”, which may play an important role in facilitating or restricting the substrate/inhibitor access to the active site. The follow‐up molecular dynamics (MD) simulations on the crystal structure not only evaluated the stability of these two conformational states but also captured the interconversion between them. In addition, we extensively explored the substrate binding modes and catalytic mechanism of the protease using AlphaFold3^[^ ^20^ ^]^ and QM/MM simulations, respectively, which provides key insights into the substrate binding pocket and enables us to further develop a fluorescence resonance energy transfer‐based (FRET‐based) assay for high‐throughput measurement of the enzymatic activity of I7L protease. On this basis, we successfully designed several peptidomimetic inhibitors with IC_50_ values as low as 69 nM, significantly more potent compared to previously reported orthopoxvirus I7L protease inhibitors, such as TTP‐6171^[^ ^21^, ^22^ ^]^ and E‐64.^[^ ^22^ ^]^ These findings provide an excellent starting point for further design and development of lead or candidate compounds targeting MPXV I7L protease.

## 2 Results

### 2.1 A Crystal Structure of MPXV I7L Protease

We successfully expressed the full‐length MPXV I7L protease in *Spodoptera frugiperda* (Sf9) cells, engineered with a C‐terminal His tag linked by a Tobacco Etch Virus (TEV) protease cleavage site. The protein purification process involved nickel‐affinity chromatography and proteolytic cleavage to remove the His tag, followed by ion‐exchange and gel filtration chromatography for further purification. The resulting I7L protease retained an additional segment with ENLYFQ sequence at its C‐terminus, namely the C‐tagged protease, which eluted at a position corresponding to a dimeric form in size‐exclusion chromatography (Figure S1a, Supporting Information). This protease was incubated with its substrate M4R and then analyzed by SDS‐PAGE. The appearance of smaller products in the gel suggests the efficient cleavage of M4R by the protease (Figure S2, Supporting Information). This demonstrates that the purified protease is active. Crystallization trials, followed by optimization, yielded well‐diffracting crystals, enabling the structure determination at 2.6 Å resolution (Table S1, Supporting Information). The resolved structure contains protomer A with well‐defined electron density for all amino acids (residues 1–423) and protomer B displaying missing or poorly defined electron density for residues 1–11 and 136–158.

**Figure 1:**
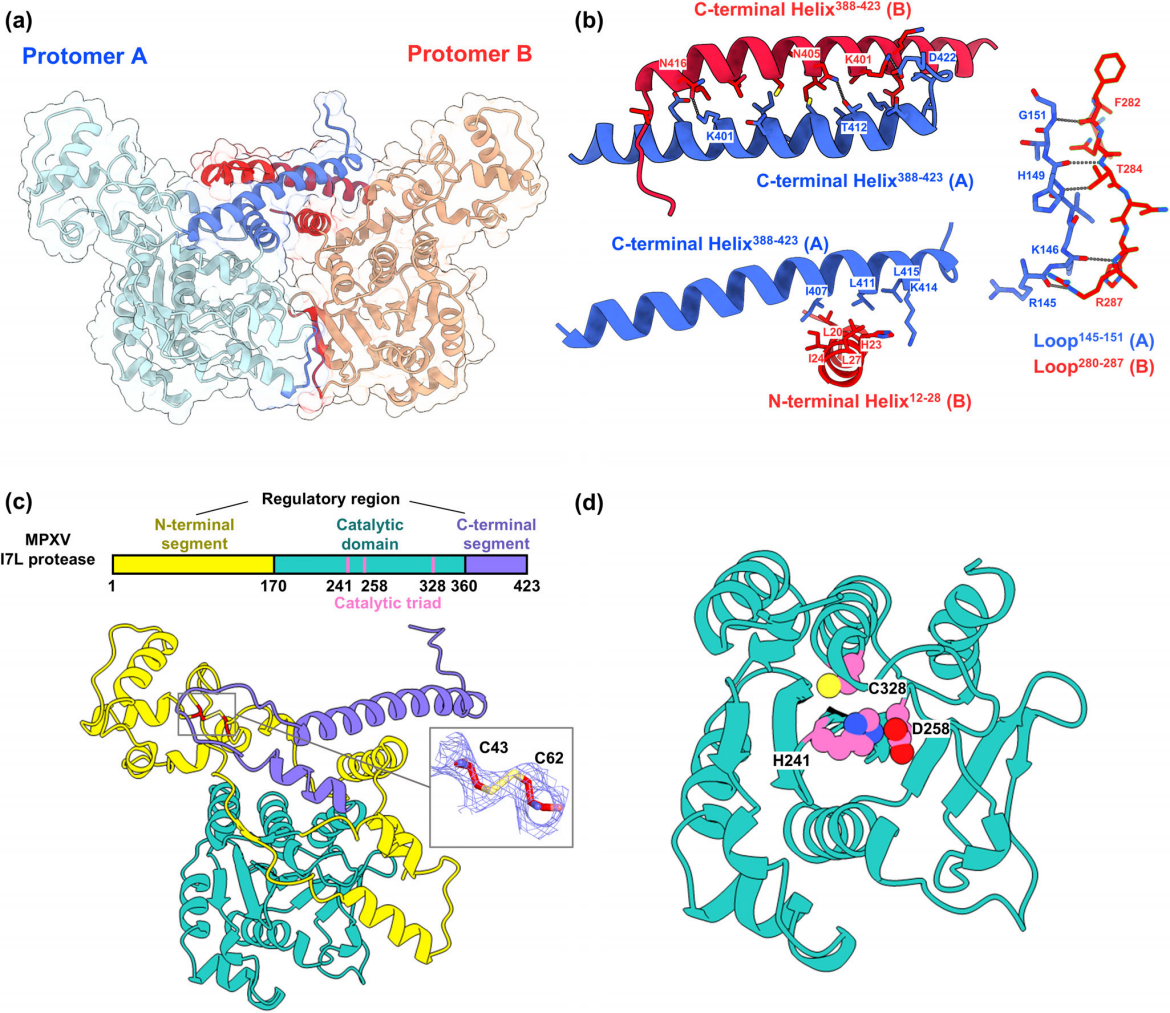
The crystal structure of C‐tagged MPXV I7L protease. a) Cartoon and surface representation of a homodimer of the MPXV I7L protease. Protomer A is shown in pale cyan, and protomer B in wheat. In protomer A, the N‐ and C‐terminal helices, along with residues 145–151 at the dimer interface, are highlighted in blue, while in protomer B, the corresponding N‐ and C‐terminal helices and residues 280–287 at the dimer interface are highlighted in red. b) Enlarged view of interactions between protomers A (blue) and B (red) at the dimer interface. c) Cartoon representation of protomer A. The N‐terminal segment is shown in yellow, the C‐terminal segment in purple, and the catalytic domain in teal. d) Close‐up view of the catalytic domain of MPXV I7L protease. The catalytic triad residues (His241‐Asp258‐Cy328) are depicted as pink spheres.

The crystal structure of C‐tagged MPXV I7L protease shows a dimeric configuration, with two protomers (A and B) arranged symmetrically to form a butterfly‐like shape (**Figure** **1**a). Such a dimeric assembly is stabilized by multiple interactions between the two protomers (Figure 1a,b). The two C‐terminal helices, one from each protomer, engage in extensive hydrophobic as well as hydrogen‐bonding interactions, reinforcing the dimer interface (Figure 1b). Additional hydrophobic interactions occur between the C‐terminal helix of one protomer and an N‐terminal helix of the other protomer (Figure 1b), and residues 145–151 of protomer A also form four hydrogen bonds with residues 280–287 of protomer B, significantly enhancing the stability of the dimer (Figure 1b). Together, these interactions generate a robust dimeric structure that is essential for the structural integrity and function of the I7L protease.

Each protomer of the I7L protease dimer consists of a regulatory domain and a catalytic domain (Figure 1c). The former includes an N‐terminal segment (residues 1–170) and a C‐terminal segment (residues 360–423). The N‐terminal segment is relatively flexible but subject to a disulfide bond (Cys43‐Cys62) (Figure 1c). It is noteworthy that the regulatory domain of I7L protease differs significantly from those of other CE clan proteases (Figure S3, Supporting Information). Both the N‐terminal and C‐terminal helices of the regulatory domain are critical for the dimerization of MPXV I7L protease, representing the first example in the CE clan that the regulatory domain is essential to the dimer assembly. The catalytic domain (residues 171–359) adopts a structural fold similar to other CE clan proteases, despite low sequence similarity (Figure 1d; Figure S3, Supporting Information). This domain consists of a range of β‐sheets sandwiched between upper and lower helices. The catalytic residue Cys328 is positioned at an upper helix, while the other two (His241 and Asp258) are located in a loop connecting the central β‐sheet (Figure 1d). This structural arrangement effectively aligns the catalytic triad and facilitates the binding and cleavage of protease substrates.

### 2.2 Conformational Switches of the Cap Region in MPXV I7L Protease

**Figure 2:**
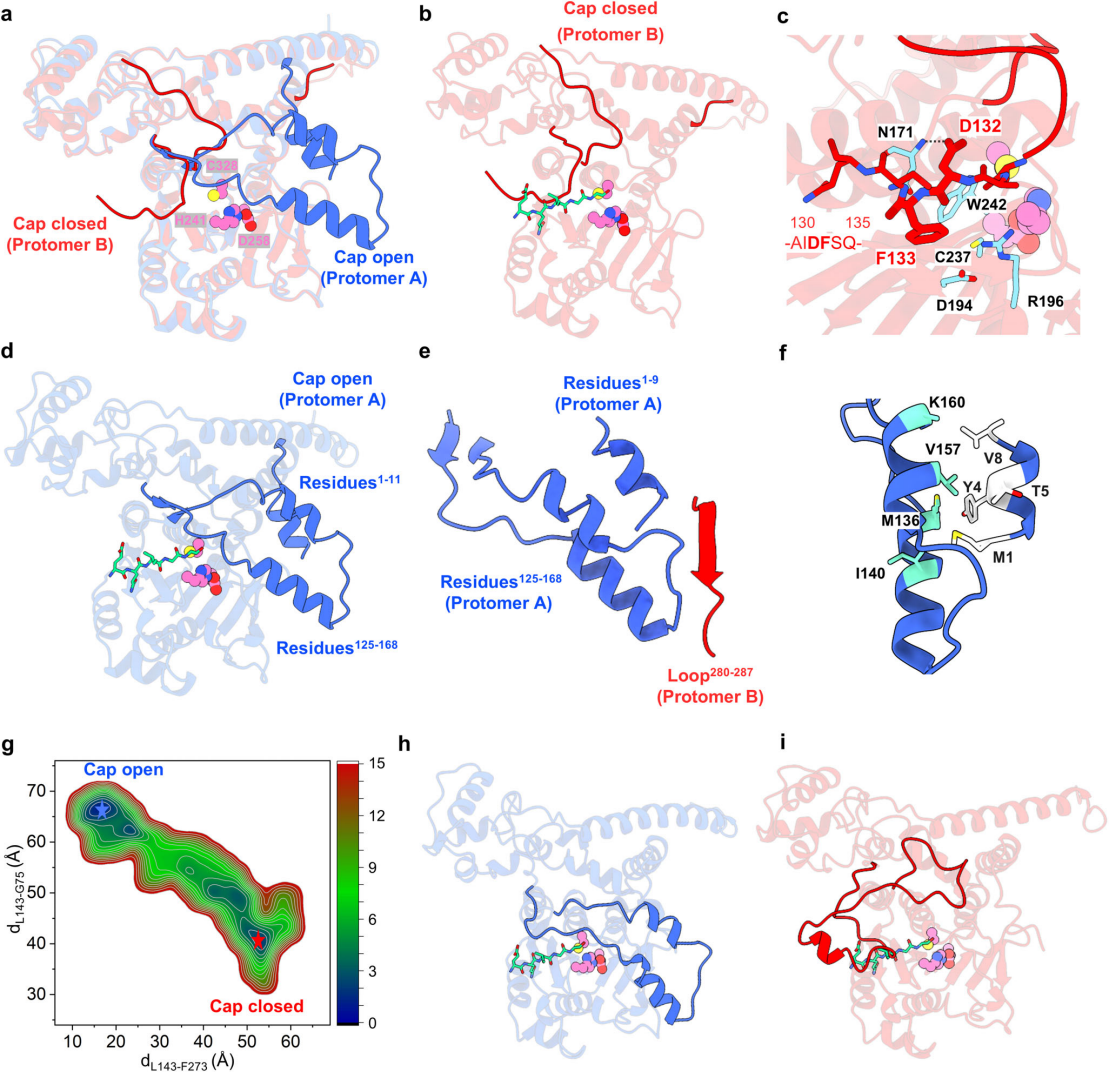
Conformational changes of the cap region in MPXV I7L protease revealed by crystal structures and MD simulations. a) Superimposition of protomers A and B of the crystal structure of C‐tagged MPXV I7L protease (PDB code: 9LIK). Protomer A and B are shown in red and blue cartoons, respectively. The catalytic triad residues are depicted as pink spheres. b) Overlay of the catalytic domain of MPXV I7L protease (protomer B, red cartoons) with the catalytic domain of Ulp1 protease in complex with SUMO3. And only the segment of SUMO3 that bound to the substrate‐binding pocket of Ulp1 (residues 94–98) is shown in green sticks. c) Interactions of Asp132 and Phe133 with the substrate‐binding pocket of protomer B. Residues Asp132 and Phe133 are shown as red sticks, while the interacting residues in the active site are displayed as cyan sticks. d) Overlay of the catalytic domain of MPXV I7L protease (protomer A, blue cartoons) and the catalytic domain of Ulp1 protease in complex with SUMO3. And only the segment of SUMO3 that bound to the substrate‐binding pocket of Ulp1 (residues 94–98) is shown in green sticks. e) Cartoon representation of the region stabilizing the “cap‐open” conformation in protomer A. f) Interactions formed between the cap region and the N‐terminal helix of protomer A. g) The free energy landscape (FEL) calculated based on the MD trajectory shows the conformation distribution of the cap region of MPXV I7L protease. The FEL was drawn along two coordinates: the distances between residues from Leu143 to Gly75 in one protomer (y‐axis) and to Phe273 in the other protomer (x‐axis). The color gradient indicates the population density of each conformation, with blue and red representing the most and the least populated ones, respectively. The contours in the 2D subspace are spaced at intervals of 1.0 kcal mol^−1^. h,i) Overlay of the catalytic domain of representative snapshots for the most populated “cap‐open” h) and “cap‐closed” i) conformations extracted from the MD trajectory with the catalytic domain of Ulp1 protease in complex with SUMO3. Only the segment of SUMO3 that bound to the substrate‐binding pocket of Ulp1 (residues 94–98) is shown in green sticks.

Superimposition of the two protomers (A and B) in the crystal structure of C‐tagged MPXV I7L protease reveals that the segment spanning residues 125–168 adopts markedly different conformations although most structural regions of the two protomers are identical (**Figure** **2**a). This segment, located near the catalytic triad, was further analyzed by aligning protomers A and B with the crystal structure of the Ulp1 protease (CE clan, C48 family) bound with a substrate, small ubiquitin‐like modifier 3 (SUMO3) protein^[^ ^23^ ^]^ (Figure S4, Supporting Information). The alignment clearly shows the positioning of residues 125–168 relative to the substrate‐binding site in each protomer. In protomer B, this segment extends into the substrate‐binding pocket, with residues 136–158 exhibiting flexibility or disorder. Notably, residues 130–135 partially overlap with the SUMO3‐binding region in the Ulp1 protease (Figure 2b), suggesting the occupation of the substrate‐binding pocket by these residues. Specifically, Asp132 forms a hydrogen bond with Asn171 in the active site, while Phe133 occupies a hydrophobic site by forming hydrophobic interactions with Asp194, Arg196, Cys237, and Trp242 (Figure 2c). This segment acts like a cap covering the active site, its conformation is thus described in terms of “cap‐open” and “cap‐closed” states in the following. In the “cap‐closed” conformation shown in protomer B, residues 125–168 obstruct the substrate access to the catalytic triad, maintaining the protease an inactive conformation. Intriguingly, protomer A adopts a “cap‐open” conformation (Figure 2d), in which the C‐terminus remaining ENLYFQ segement from another copy of protease in the crystallographic asymmetric unit occupies the substrate‐binding pocket, expelling the cap region from the active site (Figure S5, Supporting Information). This “cap‐open” conformation is stabilized by multiple hydrogen bonds formed between residues 280–287 of protomer B and the cap region of protomer A (Figure 1b and Figure 2e). Additionally, residues 1–11 of protomer A adopt a helical structure that interacts hydrophobically with the cap region (Figure 2e,f), further stabilizing the “cap‐open” conformation. It is noteworthy to point out that such an N‐terminal helix is disordered in protomer B (Figure 2a,b). Therefore, the different conformations of the cap region (residues 125–168) displayed in the two protomers of our crystal structure might be associated with the active and inactive conformations of MPXV I7L protease.

To investigate the conformational states of the cap region in the absence of the ENLYFQ segment at the C‐terminus of I7L protease, we expressed the protease again with a new construct in which an N‐terminal His tag is linked via a TEV protease cleavage site (N‐tag) to exclude the ENLYFQ sequence. Size‐exclusion chromatography analysis shows that this N‐tagged protease also eluted at the position corresponding to a dimeric form (Figure S1b, Supporting Information). Furthermore, incubation with substrate M4R followed by SDS‐PAGE analysis confirmed efficient cleavage (Figure S2, Supporting Information). The N‐tagged protease was crystallized under two different conditions, both of which yielded high‐quality crystals. X‐ray diffraction analysis successfully determined two structures, with space groups *I*4 and *P*2_1_2_1_2_1_ and resolutions of 2.2 and 2.3 Å, respectively (Table S1, Supporting Information). Remarkably, these two crystal structures, despite belonging to different space groups, present similar structural configurations in which the N‐tagged MPXV I7L protease forms a dimer but both protomers adopt the “cap‐closed” conformation, similar to that shown in protomer B of the C‐tagged protease (Figure 2b; Figure S6, Supporting Information). The variation in the cap region observed across our three crystal structures suggests that the “cap‐open” conformation, captured in protomer A of the C‐tagged protease crystal structure, is induced by the binding of the tailed ENLYFQ segment, which somehow mimics substrate binding to the protease.

Despite these crystal structures exhibit different conformations of the protease, one with a “cap‐closed” state in both protomers and the other showing a mixture of “cap‐closed” and “cap‐open” states, these proteases used for crystallization are both active in solution as they are able to cleave the substrate M4R. It is thereby inferred that the cap region may be flexible and can quickly switch from a closed state to an open conformation to recognize the substrate. To explore the conformational flexibility of the cap region, we conducted a 1‐µs conventional MD simulation on the crystal structure of the C‐tagged protease. Of note, only a dimer of the protease was used in the MD simulations so as to eliminate the binding of the ENLYFQ segment from another copy in the crystallographic asymmetric unit and to exclude the impact of crystal packing. As shown in Figure S7a,b (Supporting Information), while each protomer in the dimeric protease maintains its overall structure, the cap region in protomer A adopts the crystallographically observed “cap‐open” conformation (with small root‐mean‐square deviations (RMSDs)) whereas in protomer B it highly fluctuates around the starting “cap‐closed” state (with a large value of RMSDs). The root‐mean‐square fluctuation (RMSF) values of protomer A are generally lower than those of protomer B, particularly in the cap region (Figure S7c,d, Supporting Information). These results imply that the cap region in the open conformation can be as stable as that in the closed conformation even without the binding of the ENLYFQ segment.

Next, to investigate whether the cap region could act as a conformational switch toggling between “open” and “closed” states, we conducted a set of sequential enhanced‐sampling MD simulations implemented with Gaussian accelerated molecular dynamics (GaMD) technique on the same system used for conventional MD simulations.^[^ ^24^ ^]^ The frame with the largest motion tendency on the cap region of protomer A in each round of the sequential simulations was chosen as the starting structure for the next round to facilitate conformational transition of the cap. In an accumulated simulation time of ≈10 µs, the protease accomplished a conformational change from the “cap‐open” state to the “cap‐closed” state, demonstrating the structural flexibility of the cap region. Intriguingly, the simulation‐captured “cap‐open” and “cap‐closed” states, shown as the two distinct low‐energy conformations in the free energy landscape (Figure 2g), share the similar structural features as the counterparts observed in our crystal structures (Figure 2h,i). Accordingly, crystal structures determination followed by MD simulations reveal that the cap region in the regulatory domain of MPXV I7L protease is intrinsically flexible and could serve as a dynamic switch to modulate the substrate access to the active site.

Overall, the present work provides novel insights into the structural features and regulatory mechanisms of CE clan proteases, and in particular lays a foundation for studying the dynamic structure‐function relationships of MPXV I7L protease and its homologs.

### 2.3 Substrate Recognition and Catalytic Mechanism

**Figure 3:**
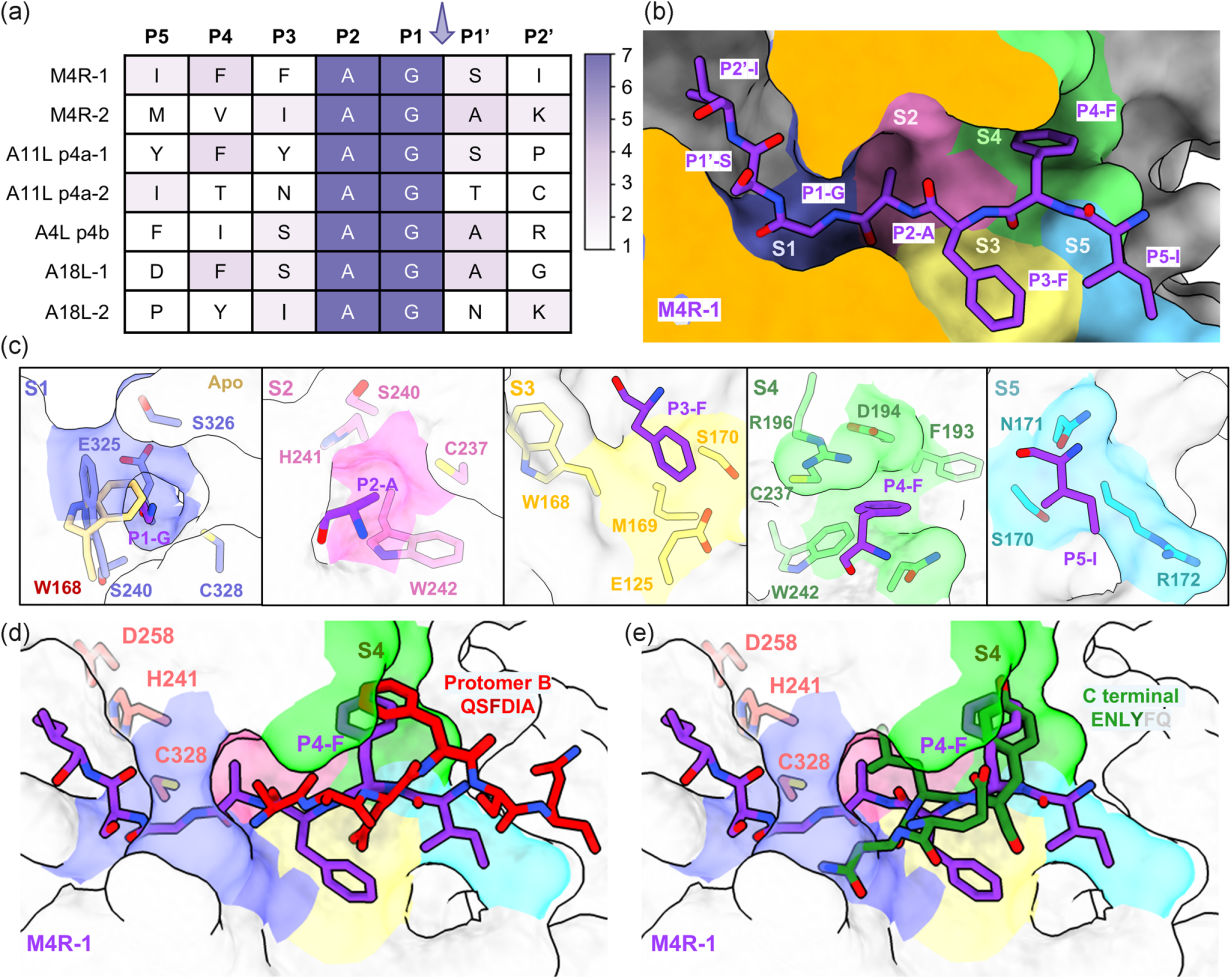
Substrate binding modes and subsites of MPXV I7L protease. a) Substrate sequences (P2’–P5) subjected to the cleavage by MPXV I7L protease. The sequences were derived from the MPXV polyproteins with reference to vaccinia virus I7L protease substrates cleavage motifs. The cleavage sites are marked with arrows. b) Molecular surface representation of M4R‐1 binding to MPXV I7L protease predicted by AlphaFold3. M4R‐1 is shown in purple sticks. The subsites S1‐S5 are colored as purple, pink, yellow, green, and sky blue, respectively. c) Residue compositions of each subsite of MPXV I7L protease. d, e) Overlay of the binding modes of M4R‐1 with those of residues 130–135 (QSFDIA) d) in protomer B and the C‐terminal residues (ENLYFQ) e) bound in protomer A. The MPXV I7L protease is represented by molecular surface. The substrate M4R‐1, residues 130–135, and the C‐terminal residues is represented by purple, red, and dark green sticks, respectively. The color‐codes of subsites S1‐S5 are as follows: S1 (purple), S2 (pink), S3 (yellow), S4 (green), and S5 (cyan).

Previous studies have shown that vaccinia virus I7L protease cleaves two terminal sites in polyproteins including L4R, A10L P4a, and A17L, and a single site in A3L P4b by targeting the conserved AG/X motif, which is crucial for viral core maturation.^[^ ^5^, ^6^, ^7^ ^]^ The vaccinia and MPXV I7L proteases share 99% sequence identity, only differing in six residues distal from the active site (Figure S8a, Supporting Information). Their substrates also exhibit 97%–99% sequence homology (Figure S8b, Supporting Information), with identical amino acids at P2'‐P5 positions of six cleavage motifs, except for a P3 variation in the second cleavage motif of A18L/A17L (Figure S8c, Supporting Information). Given these similarities, we speculated that the MPXV I7L protease also cleaves the conserved AG/X motifs of MPXV polyproteins (M4R, A11L P4a, A18L, and A4L P4b) homologous to those of vaccinia virus. Consequently, we analyzed the P2'–P5 positions of the seven cleavage motifs involved in these four substrates, revealing a high degree of sequence conservation (**Figure** **3**a). The P2 alanine (A) and P1 glycine (G) residues are consistently present at all motifs. The P1′ position is frequently occupied by an amino acid with small side chain, such as alanine (A) and serine (S). At the P4 position, hydrophobic residues predominate, usually aromatic amino acids. In contrast, the P2', P3, and P5 residues are highly variable, exhibiting higher side‐chain tolerance.

To further explore the structural basis for substrate recognition, we employed AlphaFold3 to predict the binding modes of seven substrate peptides (namely cleavage motifs) extracted from the polyproteins including M4R, A11L P4a, A18L, and A4L P4b. Except for A11L P4a‐2 (ITNAGTC), the other six cleavage motifs fit well into the substrate‐binding pocket of MPXV I7L protease with a similar binding mode (Figure 3b; Figure S9, Supporting Information). In these complexes, the catalytic cysteine (Cys328) is positioned in close proximity to the carbonyl carbon of the P1 residue of each substrate (Figure 3; Figure S9, Supporting Information), suitable for the follow‐up catalytic reaction. A comparative structural analysis between I7L protease in complex with M4R‐1, as predicted by AlphaFold3, and the ligand‐free, cap‐closed conformation revealed steric occlusion of the substrate‐binding site by the cap region spanning Ala130 to Ser134, suggesting that a conformational opening of this structural motif is required for substrate accommodation (Figure S10a, Supporting Information). Notably, in the predicted complexes, the cap region adopts a conformation closely resembling the “cap‐open” state observed in the crystal structure of Protomer A of C‐tagged I7L protease (Figure S10b,c, Supporting Information), further supporting the functional relevance of cap dynamics in substrate recognition and binding.

A comparative structural analysis of the corresponding S1–S5 subsites of the protease, which are crucial for inhibitor design, revealed their distinct features and roles in substrate binding. S1, S2, and S4 subsites are embedded within the protease, while S3 and S5 subsites are solvent‐exposed (Figure 3b). The S1 subsite features a small cavity where the substrate binding induces a side‐chain conformation shift of Trp168 (Figure 3c), creating a compact structure that likely explains the strict conservation of glycine at the P1 position. Interestingly, MD simulations of the apo MPXV I7L protease revealed that the side chain of Trp168 can spontaneously shift away from the catalytic triad to create space for substrate entry, but only when the cap region adopts the open conformation (Figure S7e,f, Supporting Information). The S2 subsite, formed by residues Ser240, His241, Cys237, and Trp242, creates a small cavity (Figure 3c). The S4 subsite, comprising Trp242, Arg196, Asp194, and Phe193, forms a relatively larger cavity compared to S1. This subsite strongly prefers aromatic amino acids at the P4 position, which is indeed often aromatic in the substrates. For example, in the “cap‐open” conformation, Phe132 occupies the S4 subsite. In addition, in the crystallographic asymmetric unit the additional ENLYFQ segment occupies the substrate pocket, with a tyrosine (Y) specifically interacting with the S4 subsite (Figure 3e). The S3 and S5 subsites, in contrast, are solvent‐accessible and exhibit high tolerance. The S3 subsite contains polar residues like Ser170 and Glu125, while the S5 subsite features Ser170, Asn171, and Arg172. Therefore, the structure prediction of the MPXV I7L protease in complex with peptide substrates provides a clear depiction of each subsite of the substrate‐binding pocket, facilitating the subsequent structure‐based inhibitor design.

**Figure 4:**
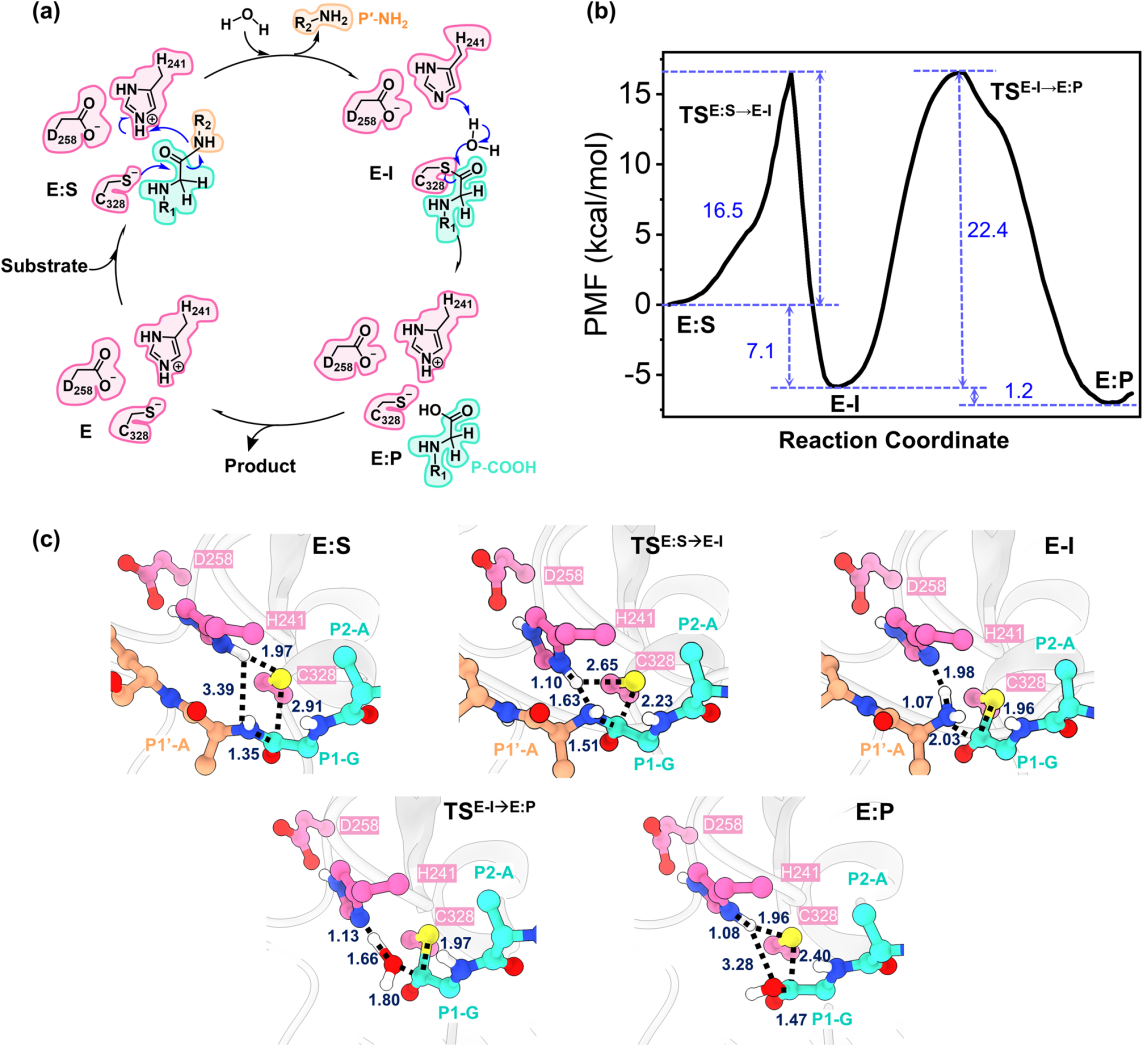
The catalytic mechanism of MPXV I7L protease revealed by QM/MM calculations. a) An overall scheme of the substrate proteolysis cycle of MPXV I7L protease. b) Free energy profiles associated with the acylation and deacylation reactions performed by the protease to proteolyze the substrate resulted from the QM/MM MD simulations. c) Representative structures of different states in the proteolysis cycle as shown in panel b. Distances are in angstroms.

Building on the above analysis of substrate binding modes, we next investigated the catalytic mechanism of MPXV I7L protease by QM/MM calculations. In principle, the reaction mechanism of cysteine proteases includes three steps:^[^ ^25^ ^]^ 1) the protease and substrate associate to form a non‐covalent complex, 2) the nucleophilic attack of the catalytic cysteine toward the P1 residue of the substrate releases the fragment corresponding to P′ residues (P′‐NH_2_ fragment) and forms a covalent acyl‐enzyme complex (the acylation step, E:S→E‐I in **Figure** **4**a), 3) the acyl‐enzyme complex is hydrolyzed to release the fragment corresponding to P residues (P‐COOH fragment) and the active site is then recovered to the initial state for the next catalytic cycle (the deacylation step, E‐I→E:P in Figure 4a). In the catalytic triad of MPXV I7L protease, Cys328 serves as a nucleophile, His241 acts as a general acid‐base, and Asp258 pairs with His241 to facilitate the deprotonation of Cys328. Our previous studies of cysteine proteases like SARS‐CoV‐2 3CL^pro^ and PL^pro^ have suggested that the catalytic cysteine and histidine often exist in an ion pair form.^[^ ^26^, ^27^ ^]^ Additionally, neutron diffraction crystallography experiments directly observed the ion pair form of Cys145‐His41 in apo SARS‐CoV‐2 3CL^pro^.^[^ ^28^ ^]^ Accordingly, we defined the protonation states of Cys328 and His241 of MPXV I7L protease as an ion pair as well (Cys328^−^/His241H^+^). To elucidate the detailed catalytic mechanism of MPXV I7L protease, the non‐covalent protease‐substrate complex predicted by AlphaFold3 underwent a 1 µs MD simulations. The results showed that the complex remained stable in the simulations, with the Sγ atom of Cys328 maintaining close contact with the carbonyl carbon atom of the P1 glycine (P1‐C), while the Nδ atom of His241 placed near the amide nitrogen atom of the P1′ alanine (P1′‐N) (<4.2 Å; Figure S11a,b, Supporting Information). Such a configuration is ideally for the nucleophilic attack of Cys328‐Sγ to P1‐C and the proton transfer from His241 toward P1′‐N to generate the P′‐NH_2_ fragment in the upcoming acylation reaction.

The free energy profile calculated by QM/MM MD simulations for the acylation and deacylation steps is depicted in Figure 4b. Either acylation or deacylation maintains a single transition state (TS^E:S→E‐I^ or TS^E‐I→E:P^) with a free energy barrier of 16.5 or 22.4 kcal mol^−1^, respectively. As a result, the deacylation is the rate‐limiting step in the entire proteolysis process. Such a feature of the free energy profile of MPXV I7L protease is similar to those of SARS‐CoV‐2 3CL^pro^ and PL^pro^ resulted from various computational simulations, and the calculated free energy barrier values for these proteases are also in a similar range.^[^ ^26^, ^29^, ^30^ ^]^ Additionally, the free energy level of the intermediate E‐I (the acyl‐enzyme complex) is 7.1 kcal mol^−1^ lower than that of the E:S non‐covalent complex and the final product E:P is even 1.2 kcal mol^−1^ lower than E‐I, resulting in a thermodynamically favored process of substrate proteolysis by the MPXV I7L protease.

Figure 4c shows the representative structures of important states involved in substrate proteolysis that were clustered from the QM/MM MD simulations. As compared to the non‐covalent E:S complex, in the TS^E:S→E‐I^ state, the Cys328‐Sγ atom approaches to the P1‐C atom (2.91 → 2.23 Å) and the peptide bond P1‐C‒P1′‐N distance is lengthened from 1.35 to 1.51 Å, meanwhile, the Nδ‐H proton of His241 also moves closer to the substrate P1′‐N atom (3.39 → 1.63 Å). The analysis of the QM/MM MD trajectories further indicated that in the acylation step, the nucleophilic attack of Cys328‐Sγ to P1‐C and the associated P1‐C‒P1′‐N bond breakage follow the proton transfer from His241 to the P1′‐N atom, identical to the acylation steps of SARS‐CoV‐2 3CL^pro^ and PL^pro^ described in our previous QM/MM MD simulations.^[^ ^26^, ^29^ ^]^

A P′‐NH_2_ fragment is yielded in the acylation step and is then released from the substrate‐binding pocket of MPXV I7L protease. Instead, a water molecule comes in, stays at the position neighboring His241 and the substrate P1‐C atom, and plays an important role in the deacylation step. This water is activated by hydrogen bonding to His241 and then its oxygen atom attacks the P1‐C atom to release the P‐COOH fragment (Figure 4a). A 1‐µs MD simulation on the E‐I intermediate displays that a deacylating water molecule continuously exists in between His241 and the P1‐C atom, with an existing probability of 0.52. In contrast, the existing probability of such a water molecule is close to 0 in the E:S non‐covalent complex, implying that the deacylating water might come into the binding pocket only when the P′‐NH_2_ fragment is released (Figure S11c,d, Supporting Information). In the representative structure of the TS^E‐I→E:P^ state of the deacylation reaction, the water oxygen atom approaches to the P1‐C atom (1.80 Å) without forming a covalent bond yet while the proton transfer from the water molecule to His241 is almost completed (Figure 4c). After the E:P state is reached, the hydroxyl group and the carbonyl carbon are finally bound with a distance of 1.47 Å and the Cys328‐Sγ‒P1‐C bond is broken (2.40 Å), regenerating the protease with a Cys328^−^/His241H^+^ ion‐paired catalytic triad and yielding the P‐COOH fragment. Overall, the multistep biochemical reactions of substrate proteolysis were well characterized by accurately calculated free energy profiles and atomic‐level configurations, allowing us to fully understand the catalytic mechanism of MPXV I7L protease and design covalent inhibitors.

### 2.4 Structure‐Based Design of Peptidomimetic Inhibitors

Due to the lack of high‐throughput methods to measure the proteolytic activity of MPXV I7L protease, we developed a fluorescence resonance energy transfer (FRET)‐based protease assay using N‐tagged I7L protease. We synthesized a fluorescently labeled substrate, DABCYL‐KDDFSAGAGVLDE(EDANS), designed based on the cleavage motif sequence of A18L and incorporated with a DABCYL quencher at the N‐terminus and an EDANS fluorophore at the C‐terminus. Upon protease cleavage, separation of the quencher and fluorophore results in a measurable increase in fluorescence, allowing real‐time monitoring of enzymatic activity.

Under physiological conditions (pH 7.5, 150 mM NaCl), N‐tagged I7L protease exhibited proteolytic activity toward the fluorescent substrate, with fluorescence intensity increasing as protein concentration increased (Figure S12a, Supporting Information). To investigate whether environmental factors influence I7L protease's enzymatic activity, we assessed the effects of glycerol (0%–50%), NaCl (0–800 mM), pH (6–9), and DNA on protease cleaving fluorescently labeled substrate. The results showed that glycerol significantly enhanced the enzymatic activity, with the highest activity observed at 50% glycerol (Figure S12b, Supporting Information). In contrast, increasing NaCl concentrations led to a gradual decrease in the enzymatic activity, with the highest activity observed in the absence of NaCl (Figure S12c, Supporting Information). Additionally, pH had a notable impact on the enzymatic activity, with an optimal activity observed at pH 7.5 (Figure S12d, Supporting Information). Notably, DNA selectively inhibited I7L protease processing the fluorescently labeled substrate (Figure S13a, Supporting Information), whereas the enzyamtic activity of SARS‐CoV‐2 3CL^pro^, used as a control, remained unaffected by DNA (Figure S13b, Supporting Information). These findings are consistent with previous studies on the vaccinia virus K7L protease, where glycerol, pH, and DNA were also found to modulate the enzymatic activity.^[^ ^31^ ^]^

In addition, besides the NaCl‐free condition (Figure 2g), GaMD simulations were also conducted to monitor the conformational states of the cap region of apo I7L protease under 150 mM NaCl condition (≈10 µs). In the presence of 150 mM NaCl, a transition from the “cap‐open” to the “cap‐closed” state can also be observed in the free energy landscape (Figure S14a, Supporting Information), however, the sampled “cap‐open” populations of the protease are less compared to the case without NaCl (Figure S14b, Supporting Information). This simulation result is in line with the experimental observation that the enzymatic activity of I7L protease is decreased with the increase of NaCl concentration.

Based on these results, we established an optimized assay condition with 75 mM NaCl and 50% glycerol, pH 7.5, to maximize the enzymatic activity while maintaining protein stability. Using this optimized assay, we determined the catalytic efficiency (*k*_cat_/*K*_m_) of MPXV I7L protease containing either a C‐tag (with the C‐terminal ENLYFQ fragment) or an N‐tag (with the N‐terminal GSG fragment), showing a value of 678 or 4067 M^−1^ s^−1^, respectively (Figure S15, Supporting Information). Given the relatively higher catalytic efficiency of the N‐tagged protease, it was selected for subsequent testing of inhibitory activities of compounds.

**Figure 5:**
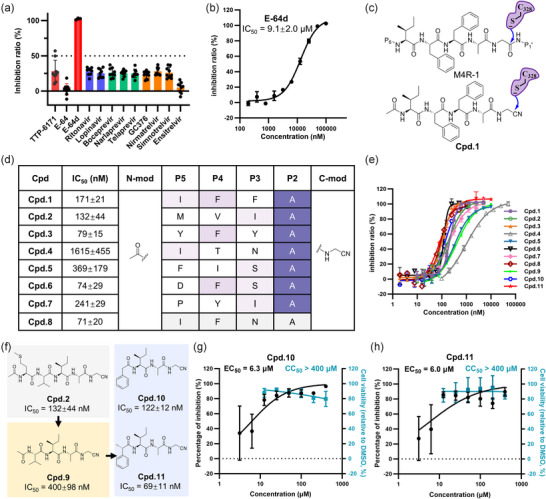
Discovery and design of inhibitors against the MPXV I7L protease inhibitors. a) Inhibitory activities of previously reported orthopoxvirus I7L protease inhibitors and a range of approved viral protease inhibitors toward the MPXV I7L protease. Except for TTP‐6171, which was tested at a concentration of 50 µM, all other compounds were evaluated at a concentration of 100 µM. b) The inhibition profile of E‐64d against the MPXV I7L protease. c) The design strategy for pentapeptide‐based covalent inhibitors. d) Structures of designed peptide inhibitors and their IC_50_ values against the MPXV I7L protease. e) Inhibition curves of compounds **1**–**11** against the MPXV I7L protease. All inhibitory activity data were resulted from the FRET‐based protease assay that we developed. Three independent experiments, each with triplicate replicates, were conducted to determine the IC_50_ values. f) The design strategy to minimize the number of peptide bonds, along with the chemical structures and IC_50_ values of the inhibitors. g,h) Dose‐response curves of compounds **10** g) and **11** h) and measured EC_50_ values for vaccinia virus replication inhibition in HeLa cells and CC_50_ values for cytotoxicity. Two independent experiments, each with triplicate replicates, were conducted to determine the EC_50_ and CC_50_ values. Error bars represent mean ± SD.

Utilizing this FRET‐based protease assay, we first determined the inhibitory activity of orthopoxvirus I7L protease inhibitors reported previously, specifically TTP‐6171^[^ ^21^, ^22^ ^]^ and E‐64^[^ ^22^ ^]^ (known as a broad‐spectrum protease inhibitor), both of which had not been characterized at the enzymatic level for their inhibition against the MPXV I7L protease. The results showed that both compounds exhibit fairly weak inhibitory activity against the protease: TTP‐6171 at a concentration of 50 µM displayed an inhibition rate of 28.1% while E‐64 even at a concentration of 100 µM showed an inhibition rate of 2.4% (**Figure** **5**a). We also tested the inhibitory activity of E‐64′s analogue, E‐64d, against the MPXV I7L protease. The results showed that E‐64d achieved an inhibition rate of 102.5% at 100 µM (Figure 5a) and an IC_50_ value of 9.1 µM (Figure 5b). Additionally, we assessed the inhibitory potential of several approved or clinical‐stage protease inhibitors, including HIV protease inhibitors (Ritonavir and Lopinavir),^[^ ^12^ ^]^ HCV NS3/4A protease inhibitors (Boceprevir,^[^ ^9^ ^]^ Narlaprevir,^[^ ^10^ ^]^ and Telaprevir^[^ ^11^ ^]^), and SARS‐CoV‐2 3CL^pro^ inhibitors (GC376,^[^ ^14^ ^]^ Nirmatrelvir,^[^ ^16^ ^]^ Simnotrelvir,^[^ ^15^ ^]^ and Ensitrelvir^[^ ^13^ ^]^). Unfortunately, none of these compounds exhibited significant inhibitory activity against this protease, with the inhibition rates consistently below 50% at a concentration of 100 µM (Figure 5a). Therefore, the FRET‐based protease assay we developed rapidly evaluated the inhibitory activity of compounds, highlighting that previously reported orthopoxvirus I7L protease inhibitors as well as a variety of approved viral protease inhibitors have rather limited potency against the MPXV I7L protease.

Guided by the structural insight into the substrate binding mode, conservation of substrate cleavage sequence, the properties of the subsites, and the covalent mechanism of substrate cleavage shown in Figure 3 and Figure 4, we designed and synthesized seven pentapeptide‐based covalent inhibitors of MPXV I7L protease (compounds **1**–**7**), derived from its natural substrates (Figure 5c,d). Each inhibitor incorporated a nitrile warhead at the P1 glycine position to facilitate covalent binding to the catalytic cysteine. The nitrile warhead was specifically chosen due to its structural compatibility with the narrow S1' subsite, as well as its clinically validated drug‐likeness demonstrated by marketed protease inhibitors such as nirmatrelvir^[^ ^16^ ^]^ and simnotrelvir,^[^ ^15^ ^]^ the latter of which was developed by our group and collaborators. Additionally, an acetyl cap was introduced at the P5 position to enhance stability. The resulting IC_50_ values of these compounds range from 74 to 1615 nM (Figure 5d,e). Notably, compound **4**, derived from a peptide whose binding mode could not be predicted using AlphaFold3, exhibits the weakest inhibitory activity (IC_50_ = 1615 nM). Sequence alignment reveals that compound **4** differs from compound **1** (IC_50_ = 171 nM) only at P3 and P4 positions. Specifically, compound **1** features a phenylalanine at both the P3 and P4 positions, while compound **4** has the P3‐asparagine and P4‐threonine. Structural analysis indicates that the S3 subsite is solvent‐exposed and thereby tolerant of a wide range of substitutions at the P3 position of substrate and inhibitor. In contrast, the S4 subsite is mainly hydrophobic, so the introduction of a polar threonine at the P4 position of compound **4** may not establish favorable hydrophobic interactions with this subsite. To address this, we replaced the polar P4 threonine in compound **4** with a phenylalanine (as in compound **1**) to give compound **8**. Such a replacement significantly enhances the inhibitory activity of compound **8**, with an IC_50_ value of 71 nM versus 1615 nM for compound **4**. The covalent docking of two compounds also revealed the P4‐phenylalanine of compound **8** engages the S4 subsite via hydrophobic interactions with Asn171, Asp194, Arg196, Cys237, and Trp242, whereas the P4‐threonine of compound **4** remains solvent‐exposed (Figure S16, Supporting Information). This suggests that the potency enhancement of compound **8** is likely driven by the favorable hydrophobic interactions with the S4 subsite. These findings highlight the importance of maintaining favorable interactions of the inhibitor with the S4 subsite to achieve strong inhibition of MPXV I7L protease.

Considering that amide bonds in peptide inhibitors are susceptible to metabolic degradation, we sought to reduce the number of peptide bonds while maintaining the inhibitory activity. Recognizing the importance of S4 subsite and the solvent‐exposed nature of S5 subsite, we first removed the P5‐methionine of compound **2**, resulting in compound **9** (Figure 5f). It exhibits potent inhibition against the MPXV I7L protease with an IC_50_ value of 400 nM, approximately three times the IC_50_ value of compound **2** (132 nM) (Figure 5e,f). This implies that the reduction of peptide bonds does not significantly compromise the potency. In addition, sequence and structure analysis of substrate preference shown in Figure 3 has suggested that the S4 subsite favors aromatic amino acids, we therefore introduced a phenyl ring at the P4 position of compound **9**, leading to the design and synthesis of compounds **10** and **11** (Figure 5f). Compound **10** shows an IC_50_ of 122 nM, while compound **11** displays even stronger inhibition, with an IC_50_ of 69 nM (Figure 5e,f). Covalent docking analysis of compound **11** revealed a binding mode highly similar to that of the substrate M4R‐1, both fitting well into respective subsites (Figure S17, Supporting Information). Specifically, its nitrile warhead forms a covalent bond with the catalytic Cys328; its P2‐alanine occupies the small hydrophobic S2 subsite; its P3‐Isoleucine resides in the solvent‐exposed S3 subsite; and the P4 group penetrates deeper into the S4 subsite compared to M4R‐1 (Figure S17, Supporting Information). Subsequently, we utilized vaccinia virus as a surrogate viral model to test the compounds’ antiviral activity at the cellular level, given the high sequence identity (98.58%) between the vaccinia virus and MPXV I7L proteases. Antiviral assays demonstrated that compounds **10** and **11** inhibit vaccinia virus replication in a dose‐dependent manner, with EC_50_ values of 6.3 and 6.0 µM, respectively (Figure 5g,h). Brincidofovir^[^ ^32^ ^]^ served as a positive control and exhibited an EC_50_ of 0.9 µM. Notably, compounds **10** and **11** showed no detectable cytotoxicity at concentrations up to 400 µM (Figure 5g,h). Accordingly, by mimicking the natural substrate binding, introducing the covalent warhead, and reducing the number of peptide bonds, we successfully obtained the potent small‐molecule inhibitors of MPXV I7L protease, offering a promising strategy as well as a good starting point for development of more drug‐like compounds.

## 3 Discussion

The highly conserved I7L protease is essential to the lifecycle of orthopoxviruses. A sequence analysis shows that there is over 92% sequence identity between the I7L protease of MPXV and those of other orthopoxviruses (Figures S18 and S19a, Supporting Information), whereas the structure of any I7L protease has not been reported. In this study, we present the first crystal structure of MPXV I7L protease and it is also the first 3D‐structure of the I7L proteases of orthopoxviruses. Such a structure determination marks a significant milestone as it solves the structure of the last uncharacterized member of the CE clan proteases that are crucial for various biological processes.^[^ ^9^, ^23^ ^]^ Although the CE clan proteases are defined by a conserved catalytic framework, they are coupled with highly variable regulatory domains responsible for their respective functional roles, resulting in very low overall sequence and structural similarity (Figure S3, Supporting Information). Our crystal structures reveal a unique dimeric assembly of MPXV I7L protease, where two regulatory domains play a pivotal role in stabilizing the dimer through extensive hydrophobic and hydrogen‐bonding interactions. Intriguingly, we identified a cap region in the regulatory domain that could act as a flexible conformational switch, modulating the ligand access to the active site. The crystal structures capture two distinct conformations of this cap region: a “cap‐open” state, where the cap is displaced away from the active site, and a “cap‐closed” state, which obstructs the binding of substrate to the active site. MD simulations further validated the stability of these two conformations and demonstrated their dynamic interconversion.

It has been considered that crystal packing interactions might contribute to stabilizing the “cap‐open” conformation which is induced by the binding of the tailed ENLYFQ segment, somehow mimicking the substrate binding to the protease. Nevertheless, the “cap‐closed” state blocks the substrate‐binding site, suggesting that a transition to the cap‐open conformation is necessary for substrate access. However, whether the “cap‐open” state observed in the crystal structure represents the predominant physiological conformation in solution requires further investigation. These structural observations raise critical questions regarding the dynamics of the cap region under physiological conditions. Does the cap region predominantly adopt an open or closed conformation, exist in a dynamic equilibrium between these states, or transition from a closed to an open state specifically upon substrate binding? We found that environmental factors, including glycerol, pH, NaCl concentration, and DNA binding, significantly influence I7L protease′s enzymatic activity. Additionally, MD simulations with/without NaCl indicate that the cap‐open populations of the protease may correlate with the NaCl concentration. Thus, it is important to determine whether and how these factors affect protease function through allosterically regulated conformational changes of the cap region. Addressing these questions in future studies will provide deeper mechanistic insights into the regulation of I7L protease.

In many viral proteases, dimerization is essential for catalytic function. For example, HIV‐1 protease relies on interfacial aspartates for catalysis, HCV NS3/4A requires an NS4A cofactor to properly orient catalytic residues, and SARS‐CoV‐2 3CL^pro^ is stabilized through domain‐swapped substrate‐binding pockets.^[^ ^18^, ^19^, ^33^ ^]^ These cases illustrate a universal coupling between the dimerization and the enzymatic activity. Unlike these viral proteases, the resolved structure of MPXV I7L protease reveals that its dimeric interface is spatially segregated from both the catalytic triad and the substrate‐binding cleft. No interfacial catalytic residues, essential cofactors, or domain‐swapped substrate‐binding pockets were observed in the I7L protease. However, the C‐tagged I7L protease structure, in which protomer A's cap region is in an open state at the dimer interface, raises the intriguing possibility that dimerization may indirectly regulate the enzymatic activity by modulating the cap region's conformation. Further investigations, such as stabilizing the monomeric form or introducing mutations that disrupt dimerization, are needed to elucidate the precise functional role of dimerization in I7L protease activity.

Moreover, we carried out detailed structural analysis of MPXV I7L protease in complex with various substrates and investigated the catalytic mechanism of the protease through QM/MM calculations, revealing key features of the substrate‐binding pocket. Notably, the hydrophobic S4 subsite preferentially accommodates aromatic residues, serving as a key factor in determining ligand binding affinity. The compact S1 subsite favors small‐sized residues, such as glycine. By contrast, the solvent‐exposed S3 and S5 subsites exhibit great flexibility, allowing to bind substrates of various sequences/structures. These insights into substrate binding provide the important foundation for the design of potent MPXV I7L protease inhibitors and also enable us to develop an efficient FRET‐based protease assay for inhibitor screening by utilizing a fluorescently labeled substrate. This high‐throughput assay is capable of real‐time measurement of the protease's enzymatic activity. As expected, we successfully designed and synthesized several covalent peptidomimetic inhibitors that demonstrate potent activities with IC_50_ values as low as 69 nM. The high conservation of the I7L protease across orthopoxviruses and monkeypox clades is supported by mutational analysis, which shows no substitutions in the binding pocket. Nearly all mutations occur more than 14 Å from compound **11**, except one particular mutation (M195I), whose main chain has a minimum distance of 5.8 Å to the compound while the side chain faces away from the compound (Figure S19b,c, Supporting Information), providing a basis for the broad‐spectrum potential of these inhibitors. While these inhibitors represent a significant advance, further development is required to enhance their pharmacokinetic properties and overall therapeutic potential. Overall, this study provides a thorough understanding of the structure, dynamics, and function of the MPXV I7L protease and showcases a successful example of the rational design of covalent peptidomimetic inhibitors.

## Conflict of Interest

The authors declare no conflict of interest.

## Author Contributions

H.S., G.W., M.X., Y.W. and J.C. contributed equally to this work. Y.X. (Y.Xu), H.S., Q.S., and L.Z. conceived and designed the project. H.S., Y.W., M.Y., T.N. and M.L. carried out protein expression and purification, crystallization, X‐ray diffraction data collection, as well as structure determination and analysis. Y.X. (Y.Xu), H.S., G.W. and M.X. designed the inhibitors. G.W. performed the chemical synthesis. H.S. and Y.W. established the molecular‐level assay and evaluated the inhibitory activity of the compounds. M.X. performed in silico structure prediction. Q.S. performed MD and QM/MM simulations. J.C., Y.X. (Y.X.), G.X. and L.Z. measured the inhibitory activity of the compounds in cells. H.S. and M.X. contributed to figure preparation. Y.X. (Y.Xu) and H.S. wrote and revised the manuscript with input from all other authors. All authors read and approved the final version of the manuscript.

## Supporting information



Supporting Information

## Data Availability

The data that support the findings of this study are available from the corresponding author upon reasonable request.
