# Supplementary material for: Dynamic Cap‐Mediated Substrate Access and Potent Inhibitor Design of Monkeypox Virus I7L Protease
Source: Adv Sci (Weinh). 2025 Apr 7;12(26):2501625. doi: 10.1002/advs.202501625 (PMC12245108; doi:10.1002/advs.202501625)
Supplement: Supplementary file 1 — Supporting Information [file ADVS-12-2501625-s001.docx]

**Supporting Information**

**Dynamic Cap-Mediated Substrate Access and Potent Inhibitor Design of Monkeypox Virus I7L Protease**

Haixia Su,^+ [a,b]^ Guoqing Wu,^+ [c,d]^ Muya Xiong,^+ [e]^ Yuhang Wang,^+ [f]^ Junyuan Cao,^+ [g,h]^ Mengyuan You,^[a]^ Yingchun Xiang,^[h]^ Tianqing Nie,^[c,d]^ Minjun Li,^[i]^ Gengfu Xiao,^[g]^ Leike Zhang,* ^[g,h]^ Qiang Shao,*** ^[a,b]^ Yechun Xu*** ^[a,b,e,f]^

[a] Dr. H. Su, M. You, Dr. Q. Shao*, Dr. Y. Xu*

State Key Laboratory of Drug Research, Shanghai Institute of Materia Medica, Chinese Academy of Sciences,

Shanghai 201203 (China)

E-mail: [qshao@simm.ac.cn](mailto:qshao@simm.ac.cn), [ycxu@simm.ac.cn](mailto:ycxu@simm.ac.cn)

[b] Dr. H. Su, Dr. Q. Shao*, Dr. Y. Xu*

University of Chinese Academy of Sciences,

Beijing 100049 (China)

[c] G. Wu, T. Nie

Lingang Laboratory,

Shanghai 200031 (China)

[d] G. Wu, T. Nie

School of Physical Science and Technology, ShanghaiTech University,

Shanghai, 201210 (China)

[e] Dr. M. Xiong, Dr. Y. Xu*

School of Pharmaceutical Science and Technology, Hangzhou Institute for Advanced Study, University of Chinese Academy of Sciences,

Hangzhou 310024 (China)

[f] Y. Wang, Dr. Y. Xu*

School of Chinese Materia Medica, Nanjing University of Chinese Medicine,

Nanjing 210023 (China)

[g] Dr. J. Cao, Dr. G. Xiao, Dr. L. Zhang*

CAS Key Laboratory of Special Pathogens, Wuhan Institute of Virology, Center for Biosafety Mega-Science, Chinese Academy of Sciences,

Wuhan 430064 (China)

E-mail: zhangleike@wh.iov.cn

[h] Dr. J. Cao, Y. Xiang, Dr. L. Zhang*

Hubei Jiangxia Laboratory,

Wuhan, 430200 (China)

[i] Dr. M. Li

Shanghai Synchrotron Radiation Facility, Shanghai Advanced Research Institute, Chinese Academy of Sciences,

Shanghai 201204 (China)

[+] These authors contributed equally to this work.

**Table of contents**

[Experimental Procedures 3](#_Toc192534786)

[Protein expression and purification 3](#_Toc192534787)

[Protein crystallization and structure determination 3](#_Toc192534788)

[Enzymatic activity and inhibition assays 4](#_Toc192534789)

[Determination of catalytic efficiency 4](#_Toc192534790)

[Structure prediction using AlphaFold3 4](#_Toc192534791)

[Covalent Docking 5](#_Toc192534792)

[Simulation system preparation 5](#_Toc192534793)

[Conventional MD simulations 6](#_Toc192534794)

[Enhanced sampling MD simulations 6](#_Toc192534795)

[Quantum Mechanics/Molecular Mechanics (QM/MM) MD simulations 7](#_Toc192534796)

[Antiviral effect of compounds against Vaccinia Virus 8](#_Toc192534797)

[Supplementary Figures and Tables 10](#_Toc192534798)

[Chemistry 30](#_Toc192534799)

[References 56](#_Toc192534800)

# Experimental Procedures

## Protein expression and purification

The cDNA of MPXV I7L protease (GenBank: AAL40526.1), fused with either a C-terminal STREPⅡ-10×His tag (C-Tag) or an N-terminal 8×His-GST tag (N-tag), was inserted into the pFASTBacHTb vector, with a Tobacco Etch Virus (TEV) protease cleavage site between the tag and the protease. Bacmids were generated in DH10Bac cells, and the resulting baculoviruses were generated and amplified in Sf9 insect cells. After infection by baculoviruses for 48 h, the cells were harvested in a lysis buffer containing 50 mM Tris (pH 8.5), 15% glycerol, 500 mM NaCl, and 2 mM 2-mercaptoethanol. The expressed protein was purified by a Ni-NTA column (GE Healthcare) and transformed into the lysis buffer containing TEV protease for removing the tag. The resulting protein sample was further purified by heparin affinity chromatography followed by a size-exclusion chromatography (GE Healthcare) with a solution containing 30 mM 4-(2-hydroxyethyl)-1-piperazineethanesulfonic acid (HEPES, pH 7.0), 10% glycerol, 500 mM NaCl, and 5 mM dithiothreitol. The purified MPXV I7L protease was concentrated to approximately 5 mg/mL for the enzymatic inhibition assay and protein crystallization.

## Protein crystallization and structure determination

Crystals of the C-tagged MPXV I7L protease with a C-terminal remaining segment (amino acid sequence: ENLYFQ) were obtained under the condition of 5-20% PEG 4000, 150 mM imidazole-MES buffer (pH 5.5-6.5), 30% glycerol, and 0.6 M halogen salts (NaF: NaBr: NaI = 1: 1: 1). Crystals of the N-tagged MPXV I7L protease in space group *I*4 were obtained under the condition of 15% PEG3350, 0.1 M Bis-Tris (pH 5.5), and 0.2 M potassium thiocyanate. Crystals of the N-tagged MPXV I7L protease in space group *P*2_1_2_1_2_1_ were obtained under the condition of 12.5% PEG 8000, 0.1 M sodium acetate (pH 5.0), and 0.2 M lithium sulfate. Crystals were flash frozen in liquid nitrogen in the presence of the reservoir solution supplemented with 20% glycerol. X-ray diffraction data were collected at beamline BL10U2 at the Shanghai Synchrotron Radiation Facility.^[1-2]^ The data were processed with HKL3000 software packages.^[3]^ The structures were solved by molecular replacement using the program PHASER^[4]^ with a search model of the AlphaFold3-predicted structure. The model was built using Coot^[5]^ and refined with the program PHENIX.^[6]^ The refined structures were deposited to Protein Data Bank with accession codes listed in Table S1. The complete statistics as well as the quality of the solved structures are also shown in Table S1.

## Enzymatic activity and inhibition assays

The inhibitory activities of compounds against MPXV I7L protease were determined using a fluorescence resonance energy transfer-based (FRET-based) protease assay. The fluorogenic substrate DABCYL-KDDFSAGAGVLD-Glu (EDANS) (GenScript, China) can be cleaved by MPXV I7L protease, generating an EDANS peptide fragment that emits strong fluorescence at excitation/emission wavelengths of 340/490 nm. The FRET-based protease assay was performed as follows. Recombinant N-tagged MPXV I7L protease was mixed with serial dilutions of each compound or HT-DNA (Deoxyribonucleic acid sodium salt from herring testes, Sigma, Product ID: D6898) in 80 μL of the assay buffer containing 30 mM Tris (pH 7.5), 75 mM NaCl, and 50% glycerol. The mixture was incubated for 10 minutes at room temperature. The final concentration of recombinant MPXV I7L protease used in the assays was 200 nM. The reaction was initiated by adding 40 μL of the fluorogenic substrate to a final concentration of 5 μM. Fluorescence signals at 340 nm (excitation) and 490 nm (emission) were immediately measured every minute for 10 minutes using a microplate reader. The reaction velocities with the added compound were compared to the reaction velocity with DMSO, and the IC_50_ values were determined by generating dose-response curves. Each experiment was performed in triplicate, and at least eight concentrations of each compound were tested to determine the IC_50_ values.

## Determination of catalytic efficiency

Either the C-tagged or N-tagged MPXV I7L protease was prepared at a final concentration of 400 nM and mixed with the fluorogenic substrate at various concentrations (80 μM, 40 μM, 20 μM, 10 μM, 5 μM, 2.5 μM, 1.25 μM, and 0.625 μM). The total reaction volume was 80 μL. Fluorescence intensity was monitored using a microplate reader. Initial reaction rates were determined by fitting the linear portion of the fluorescence curves to a straight line. The kinetic parameters, including *K*_m_ and *k*_cat_, were calculated from a double-reciprocal plot.

## Structure prediction using AlphaFold3

The sequences of MPXV I7L protease (GenBank: AAL40526.1) and four putative substrate proteins, M4R (GenBank: AAL40541.1), A11L (GenBank: AAL40579.1), A18L (GenBank: AAL40586.1), and A4L (GenBank: AAL40572.1), were retrieved from the National Center for Biotechnology Information (NCBI) database. The models of apo MPXV I7L protease dimer and in complex with the substrates were predicted using AlphaFold3.^[7]^ Since A4L has one putative cleavage site, the full-length amino acid sequence of A4L and MPXV I7L protease were submitted to AlphaFold3 for the prediction of the complex. While M4R, A11L, and A18L possess two putative cleavage sites, the full-length sequence and truncated sequences consisting the 7 (P2'–P5) or 11 (P5'–P6) residues flanking the cleavage site were used. The binding modes of all substrates (except for the second cleavage motif of A11L) with the MPXV I7L protease were successfully predicted. The most plausible models with the higher predicted local distance difference test (plddt) score were selected for structural analysis.

## Covalent Docking

The prediction of the covalent binding mode between MPXV I7L protease and inhibitors was performed by the tools implemented in the Schrödinger 2015 suite. The AlphaFold3-predicted structure of MPXV I7L protease in complex with the substrate segment (P2'-P4) of A11L p4a-1 was employed as the receptor structure. The receptor was preprocessed using the Protein Preparation Wizard,^[8]^ which facilitated the addition of hydrogen atoms and the missing residue side chains. Subsequently, the overall structure was refined using OPLS3 forced field^[9]^ with harmonic restraints on heavy atoms. The three-dimensional (3D) structures of inhibitors were yielded with LigPrep. Covalent complexes were predicted through Covalent Docking,^[10]^ wherein the catalytic Cys328 was defined as the reactive residue and the centroid of three residues (Cys328, Trp168 and Trp242) was used as the docking box center. The reaction type was determined based on the reactive group of the compound, and the covalent docking was performed with a pose prediction mode. The resultant complex models were analyzed based on docking scores.

## Simulation system preparation

The atomic coordinates of apo MPXV I7L protease dimer were achieved from the determined crystal structure of the C-tagged protease (PDB code: 9LIK). The missing loop of the cap region and the N-terminal helix in protomer B were reconstructed and refined using the cyclic coordinate descent (CCD) and kinematic closure (KIC) protocols in Rosetta V3.10.^[11-12]^ The protonation states of all titratable residues except the catalytic His241 and Cys328 that were supposed to form an ion pair were evaluated at pH 7.5 using Schrodinger suite software. All evaluated residues were found in their standard protonation states. A detailed inspection of the environment surrounding each histidine residue suggested that all histidines except His23 (positively charged) were neutral, among which His76, His214, His268, and His269 were protonated at Nδ atoms while the remaining His101, His150, His204, His286, and His288 were protonated at Nε atoms. The I7L protease system was solvated in 0 or 150 mM NaCl concentrated solution, respectively. In the former case, the system was solvated in a cubic box filled with a total of 48676 water molecules, in which one Cl^-^ ion was added to neutralize the protein charges. In the latter case, the simulation system was constructed using the CHARMM-GUI solution builder,^[13]^ generating a cubic box containing I7L protease, 178 Na^+^ and 179 Cl^-^ ions, and 62426 waters. AMBER 22 suite of program^[14]^ was employed for simulations using FF14SB force field^[15]^ and TIP3P^[16]^ for protein and water molecules, respectively.

## Conventional MD simulations

The system was initially minimized for 50,000 steps and heated to 300 K, with the protein heavy atoms being fixed using a harmonic restraint with a force constant of 10.0 kcal/mol/Å^2^. Subsequently, the protein was relaxed by two short-time steps of equilibrium at constant temperature and constant pressure of 1 atm (*NPT* ensemble): 2 ns for relaxing protein side chain and 2 ns for protein main chain. Finally, a production run lasting 1 μs using the GPU version of AMBER 22 was performed without constraints on any atoms. The atomic coordinates were saved every 10,000 steps for data analysis. The SHAKE algorithm was used to fix all covalent bonds involving hydrogen atoms, and periodic boundary conditions were used to avoid edge effects.^[17]^ The Particle Mesh Ewald method was applied to treat long-range electrostatic interactions and the cutoff distance for long-range terms (electrostatic and van der Waals (vdW) energies) was set as 10.0 Å.^[18]^ The Langevin dynamics with a collision frequency of 3.0 ps^-1^ was adopted to control the temperature.

## Enhanced sampling MD simulations

The abovementioned apo MPXV I7L protease system after short-time equilibration was used as an initial structure for sampling the conformational changes between the “cap-open” and “cap-closed” states using Gaussian accelerated molecular dynamics (GaMD).^[19]^ We performed sequential GaMD simulations following the protocol designed by Ayaz et al.^[20]^: in each round, we ran 3 independent trajectories (300 ns) and then chose the frame having the largest motion tendency on the cap region of protomer A among the trajectories as the starting structure for the 3 independent trajectories in the next round. The sequential GaMD simulations were stopped until the cap region of protomer A could reach the desired target structure. Using such a protocol, we could sample the conformational transition of the cap region from the initial “cap-open” state to the final “cap-closed” state in endurable computational time. The accumulated simulation time is ~10 μs.

Each GaMD simulation trajectory was run at the “dual-boost” level by setting the reference energy to the lower bound, one boost potential being applied to the total potential and the other to the dihedral energetic term. The average and standard deviation (SD) of the system potential energies were calculated every 700,000 steps (1.4 ns) or 800,000 steps (1.6 ns) for I7L protease in 0 or 150 mM NaCl solution, respectively. The upper limit of the boost potential SD was set to 6.0 kcal mol^–1^ for both the dihedral and total potential energetic terms. The coordinates were saved every 10,000 steps for data analysis. The free energy landscape (FEL) on specific collective variables and the recovered structure distribution of the protein was calculated with the accompanied reweighting algorithm of GaMD.

## Quantum Mechanics/Molecular Mechanics (QM/MM) MD simulations

The non-covalent complex system of MPXV I7L protease with substrate was constructed by AlphaFold3. The substrate was prepared by using the IAGAK peptide fragment, with its N- and C-termini being capped with ACE (-C(=O)-CH_3_) and NME (C(=O)-NH-CH_3_) groups. The complex system underwent 1 μs conventional MD simulation following the same process of the apo protease. The MD measured stable complex structure was then clustered to be used as an initial state for consequent QM/MM MD simulations to explore the free energy profiles associated with the protease‒substrate covalent chemical reactions. In the QM region, the sidechains of the catalytic triad (His241, Cys328, and Asp258) and a fragment of the substrate (the P1′-Ala, P1-Gly, and the peptide bonds up to the Cα atoms of P2-Ala and P2′-Ile) were involved. The remaining part of the system was described at the MM level. In the deacylation reaction step, a water molecule between His241 and the substrate P1-C atom was also included in the QM region. As the QM region crossed covalent bonds, the QM/MM boundary was chosen to cut C-C non-polar bonds and link atoms (hydrogens) were added automatically for the QM calculation without user intervention.^[21]^ A DFTB potential was used to describe the QM subsystem^[22]^ while the MM region was described by the force fields of FF14SB for protein and the remaining part of substrate, and TIP3P for water molecules. A cutoff radius of 10 Å was used for QM/MM interactions and the temperature was controlled at 300 K.

First, steered molecular dynamics (SMD)^[23]^ was performed to yield the reaction path in the QM region following specific reaction coordinate. The QM/MM SMD was run using a harmonic force constant of 500 kcal/mol/Å^2^ to pull the system along the predefined reaction coordinate. Then, multiple structures were evenly selected from SMD trajectory with an increment of the reaction coordinate of ~0.15 Å as starting points for subsequent umbrella sampling (US) simulation.^[24]^ A constraint was added along the reaction coordinate with an umbrella force constant of 100 kcal/mol/Å^2^ in each US window, making sure the sampled reaction path is overlapped among individual windows. In every window, simulation was performed for 250 ps at 300 K with a time step of 1 fs. Finally, the detailed free energy profile, in terms of potential of mean force (PMF), was calculated with the Weighted Histogram Analysis Method (WHAM).^[25]^

The reaction coordinate for the acylation reaction is: RC = CV1 + CV2, CV1: d_C328-Sγ‒P1__-C_ (the distance between Cys328-Sγ and substrate’s P1-C atoms); CV2: d_H241-Hδ‒P1′-N_ (the distance between His241-Hδ and substrate’s P1′-N atoms). Meanwhile, the reaction coordinate for the deacylation is: RC = CV1 + CV2 – CV3 – CV4, CV1: d_H241-Nδ‒water-H_ (the distance between His241-Nδ and deacylating water’s hydrogen atoms); CV2: d_water-O‒P1-C_ (the distance between deacylating water’s oxygen and substrate’s P1-C atoms); CV3: d_water-O‒water-H_ (the distance between deacylating water’s oxygen and hydrogen atoms); CV4: d_C328-Sγ‒P1-C_ (the distance between Cys328-Sγ and substrate’s P1-C atoms).

## Antiviral activity of compounds against Vaccinia virus in cells

HeLa cells were seeded at a density of 7×10^4^ cells per well in 48-well plates. After overnight incubation, cells were incubated with varying concentrations of compounds. 1 h later, cells were infected with the Vaccinia virus at an MOI of 0.1 for 2 h. The medium containing both the virus and the compound was removed, and cells were cultured with the compound-only medium. The cells were cultured for an additional 24 h. The supernatant was collected and viral DNA was extracted using a viral DNA/RNA extraction kit (Vazyme, China). Antiviral activities were evaluated by quantifying viral DNA copy number in supernatant via real-time fluorescence quantitative PCR (qRT-PCR), using F primer 5′-gatgatgcaactctatcatgta-3′ and R primer 5′-gtataattatcaaaatacaagacgtc-3′. DMSO was used in the control. The Orthopoxviral HA gene was cloned into PBS vector and used as standard. A standard curve was generated by determining copy numbers from serial dilutions of the plasmid (10^3^-10^9^ copies).

Cell viability was evaluated HeLa cells 96-well plate in triplicate for each compound concentration. All compounds were serial diluted in a maintenance medium (DMEM containing 2% FBS) with a starting concentration of 200 μM. After a 24 h incubation, the supernatant was removed, and 100 μL medium containing CCK-8 was added to cells. Absorbance at 450 nm was measured using a BioTek spectrophotometer after a 1 h incubation, and cell viability was calculated.

# Supplementary Figures and Tables


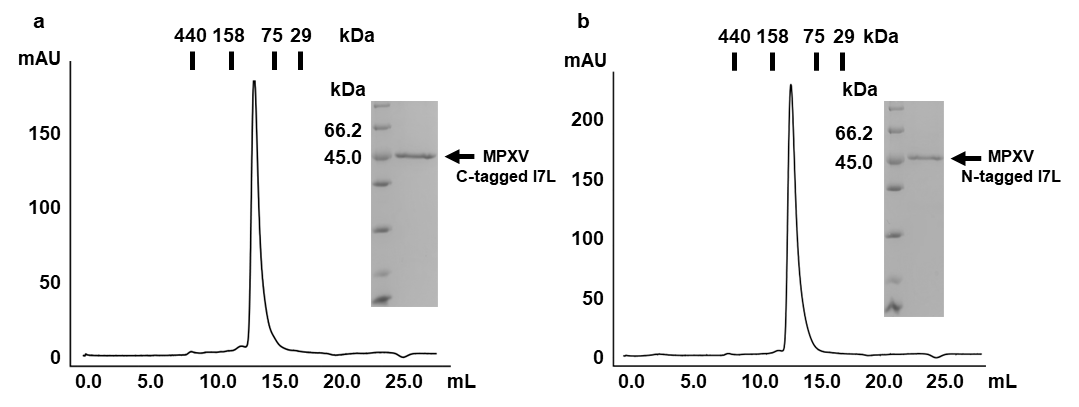


**Figure S1.** Size-exclusion chromatography (SEC) elution profiles and SDS-PAGE analysis of C-tagged (a) and N-tagged (b) MPXV I7L protease. Elution profiles were monitored at 280 nm using a Superdex 200 Increase 10/300 GL column calibrated with molecular weight standards: ferritin (440 kDa, 10.78 mL), aldolase (158 kDa, 13.12 mL), conalbumin (75 kDa, 14.36 mL), and carbonic anhydrase (29 kDa, 16.60 mL). Both C-tagged and N-tagged MPXV I7L proteases eluted at approximately 14.0 mL, consistent with their dimeric forms.

**
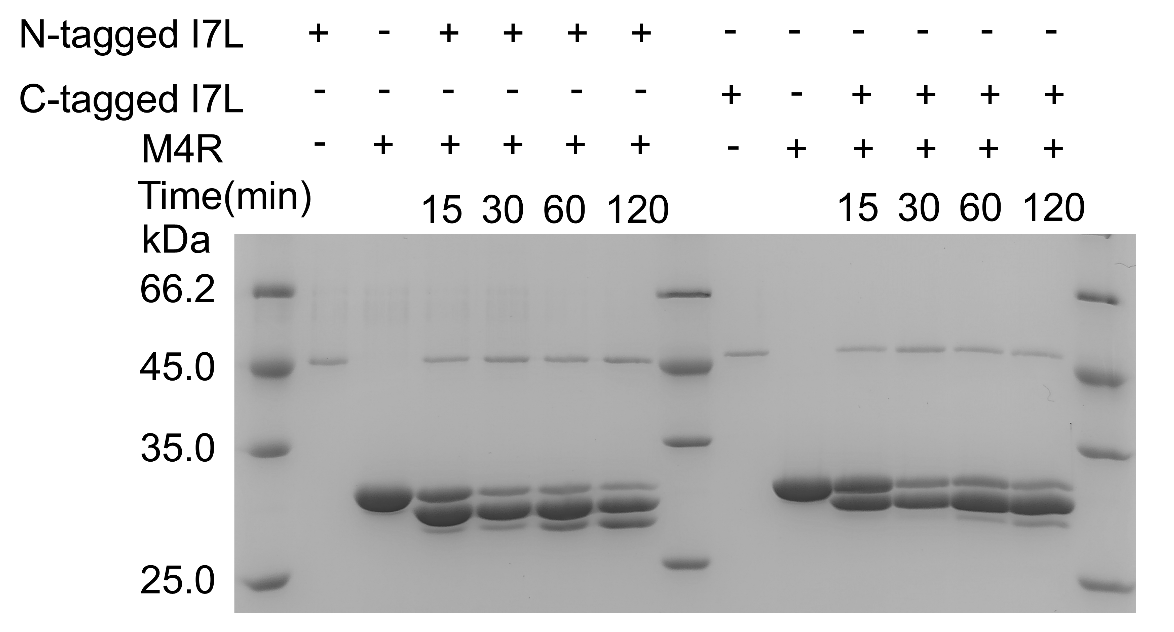
**

**Figure S2.** SDS-PAGE analysis of the MPXV I7L protease incubating with its substrate M4R.


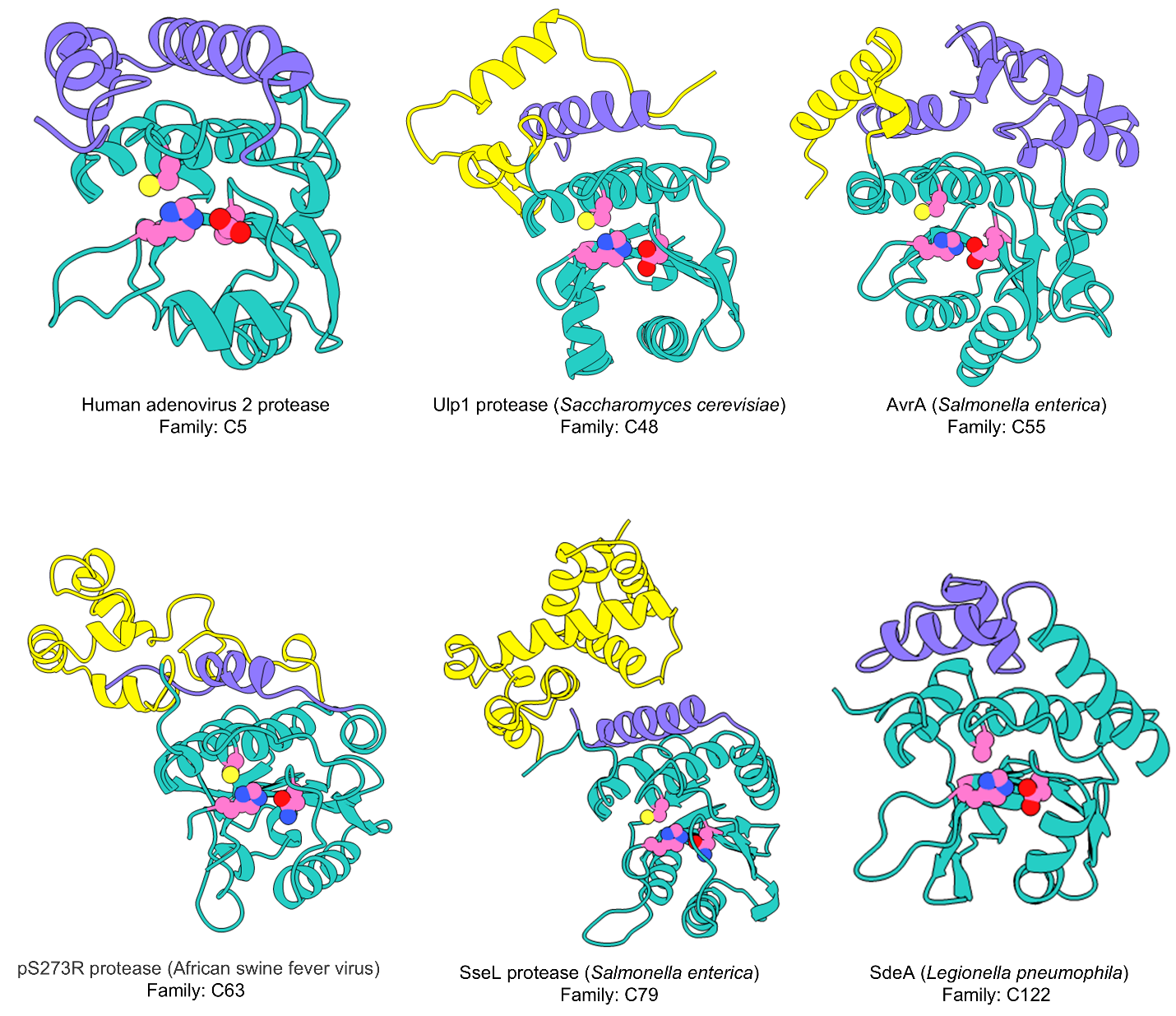


**Figure S3.** Cartoon representation of the representative proteases in Clan CE. The N-terminal and C-terminal segments are shown in yellow and purple, respectively, and the catalytic domain is shown in teal. The catalytic triad residues are highlighted as pink spheres. The classification of the family is based on the MEROPS protease database.


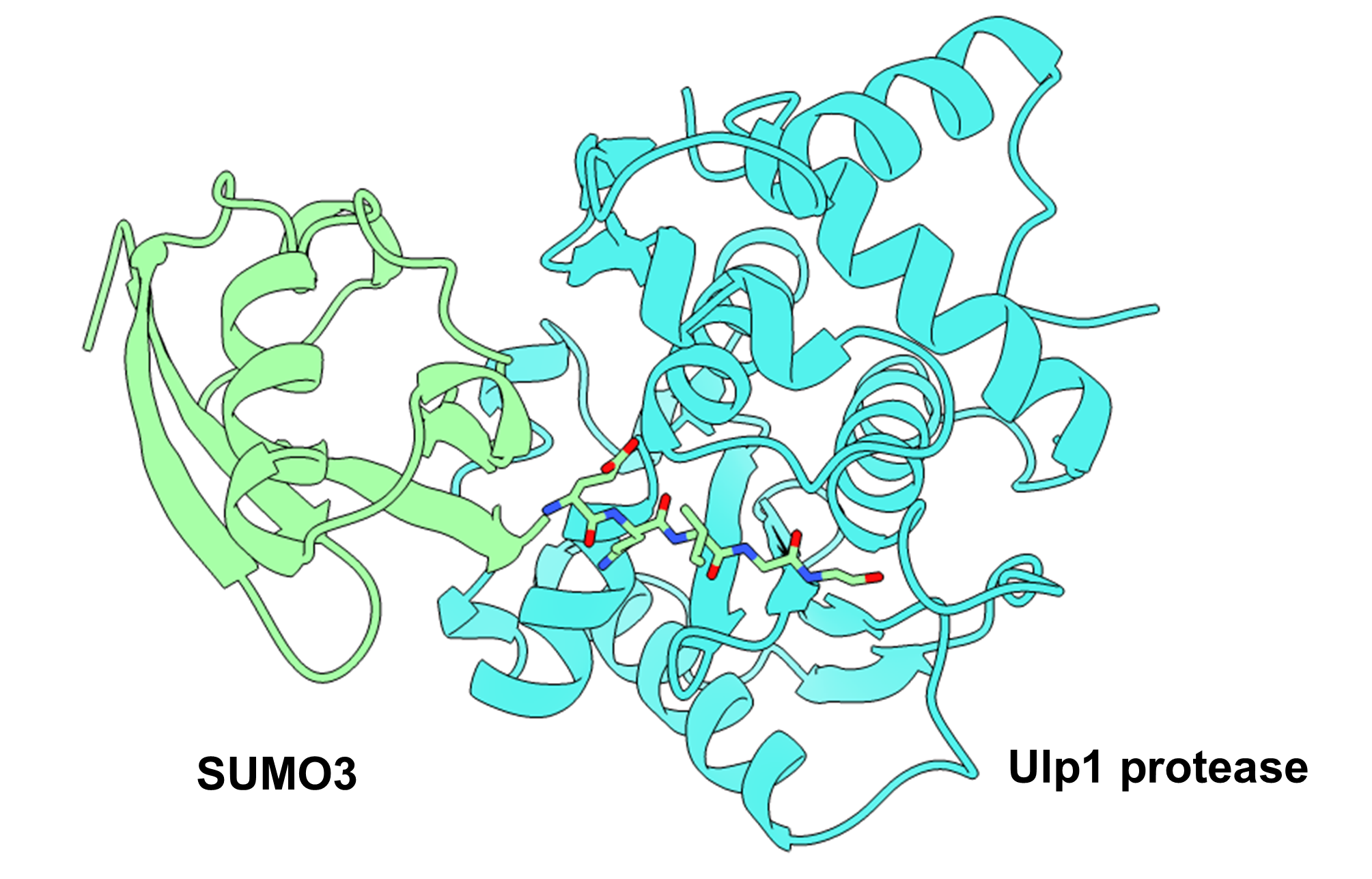


**Figure S4.** Cartoon representation of the crystal structure of Ulp1 protease in complex with SUMO3 (PDB code:1EUV). The Ulp1 protease is shown in cyan cartoons, while SUMO3 is depicted in light green cartoons. The C-terminal residues of SUMO3 are shown as light green sticks.


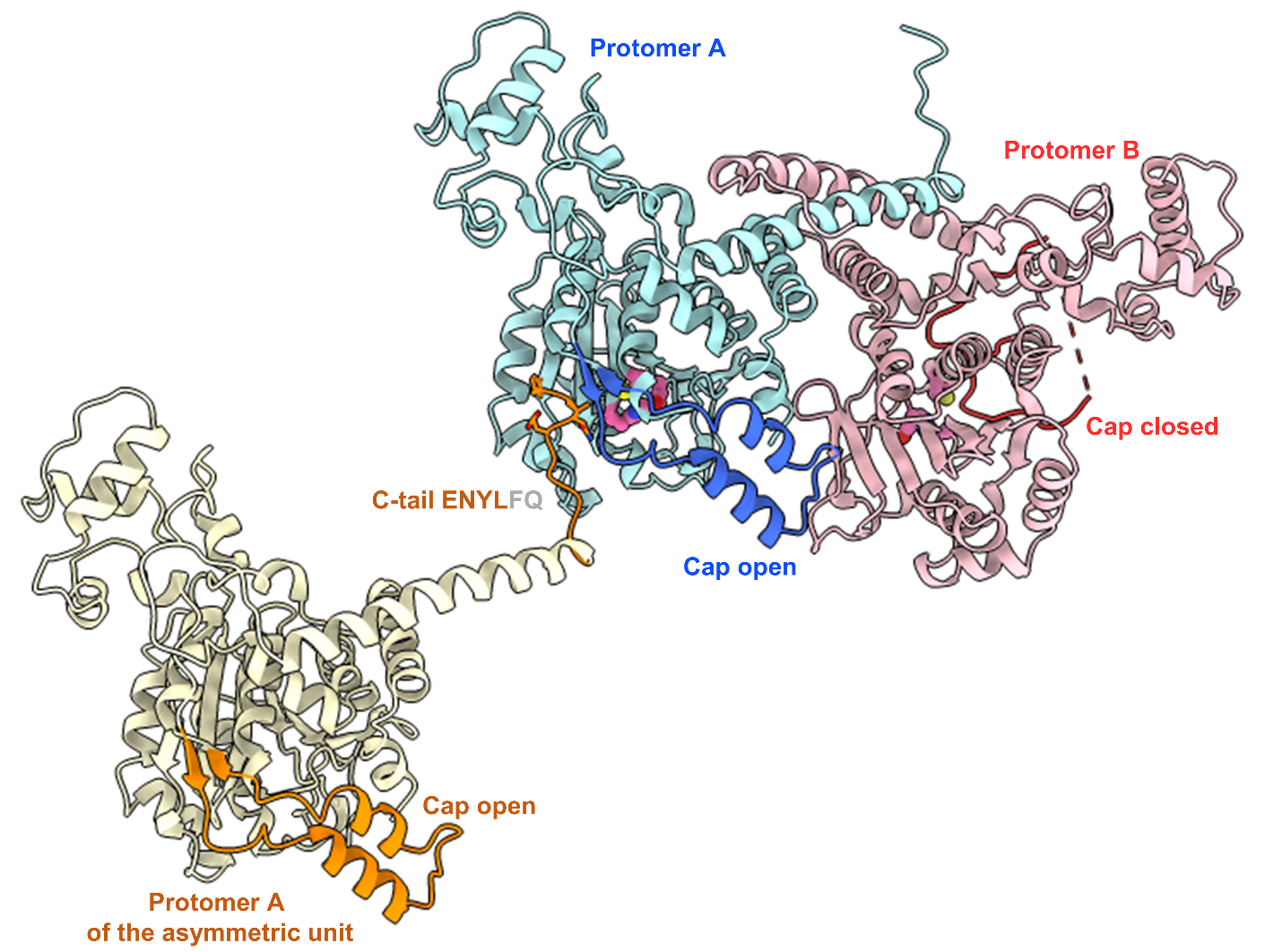


**Figure S5.** Cartoon representations of the remaining C-terminal ENLYFQ segment from another copy of the MPXV I7L protease in the crystallographic asymmetric unit, occupying the substrate-binding pocket of protomer A of the protease (PDB code: 9LIK). Protomer A, B, and the A protomer from another copy of the protease in the asymmetric unit are shown in cyan, pink, and yellow, respectively. The cap regions are highlighted in blue, red, and orange, respectively. The C-terminal ENYL residues of protomer A from another copy are depicted as orange sticks. The catalytic triad residues are highlighted as pink spheres.


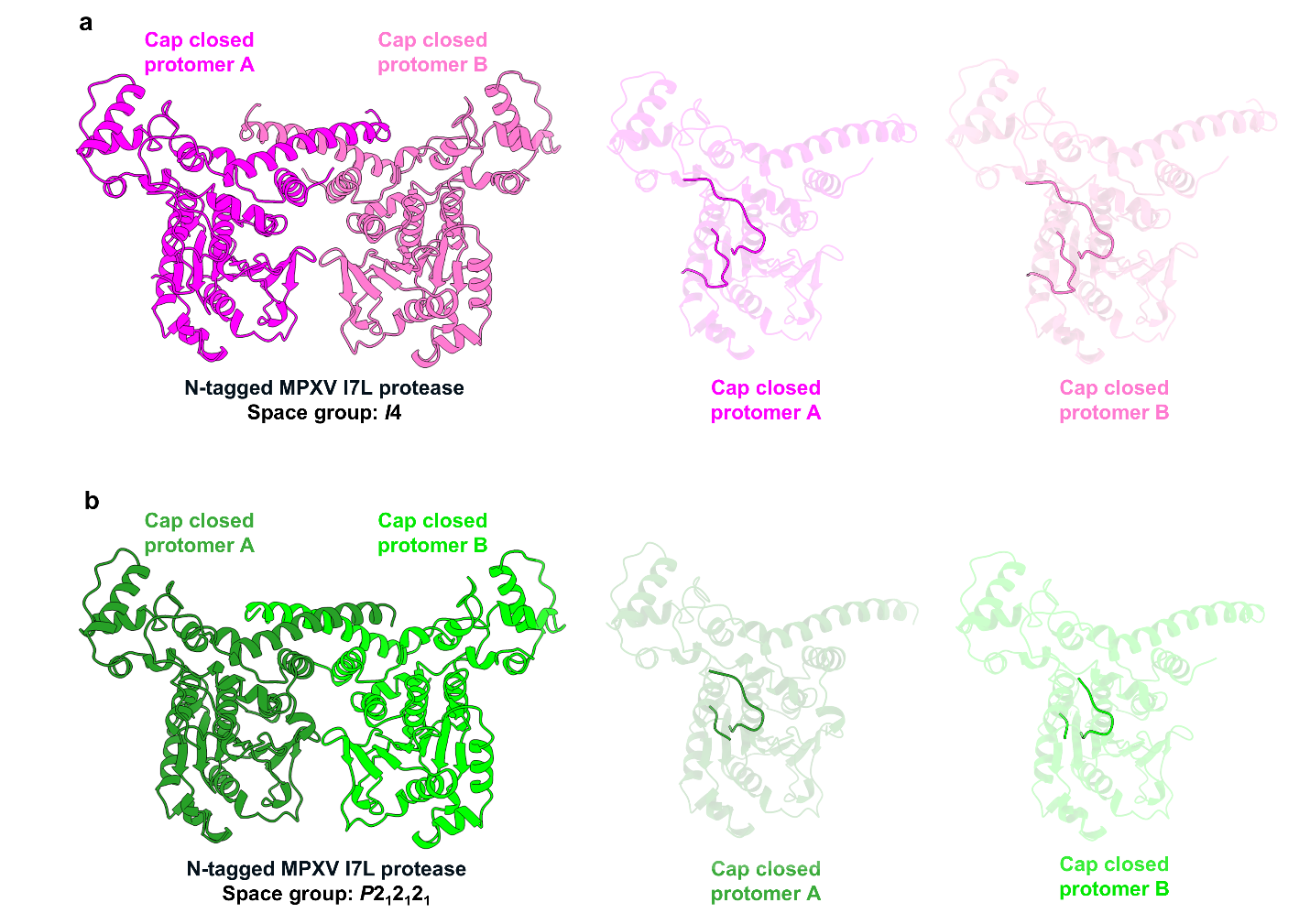


**Figure S6.** Two crystal structures of N-tagged MPXV I7L protease. (a, b) Cartoon representations of the N-tagged I7L protease in the *I*4 space group (PDB code: 9LIL) and the *P*2_1_2_1_2_1_ space group (PDB code: 9LIM) (b). The cap region of the protease is highlighted.


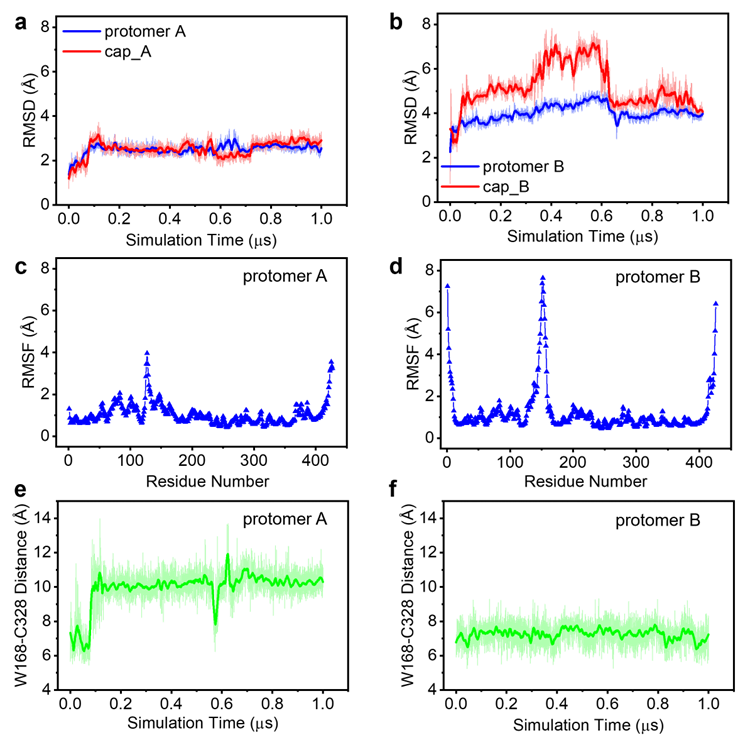


**Figure S7.** Structural dynamics of MPXV I7L protease homodimer. (a, b) Conventional MD simulation trajectory of MPXV I7L protease as indicated by the time series of the main chain root-mean-square deviations (RMSDs) for protomers A (a, blue line) and B (b, blue line), as well as the cap regions in their respective protomers (red lines). (c, d) Residue root-mean-square fluctuations (RMSFs) of MPXV I7L protease in the protomers A (c) and B (d). (e, f) Time dependence of the sidechain distance between Trp168 and the catalytic Cys328 in MPXV I7L protease for protomers A (e) and B (f).


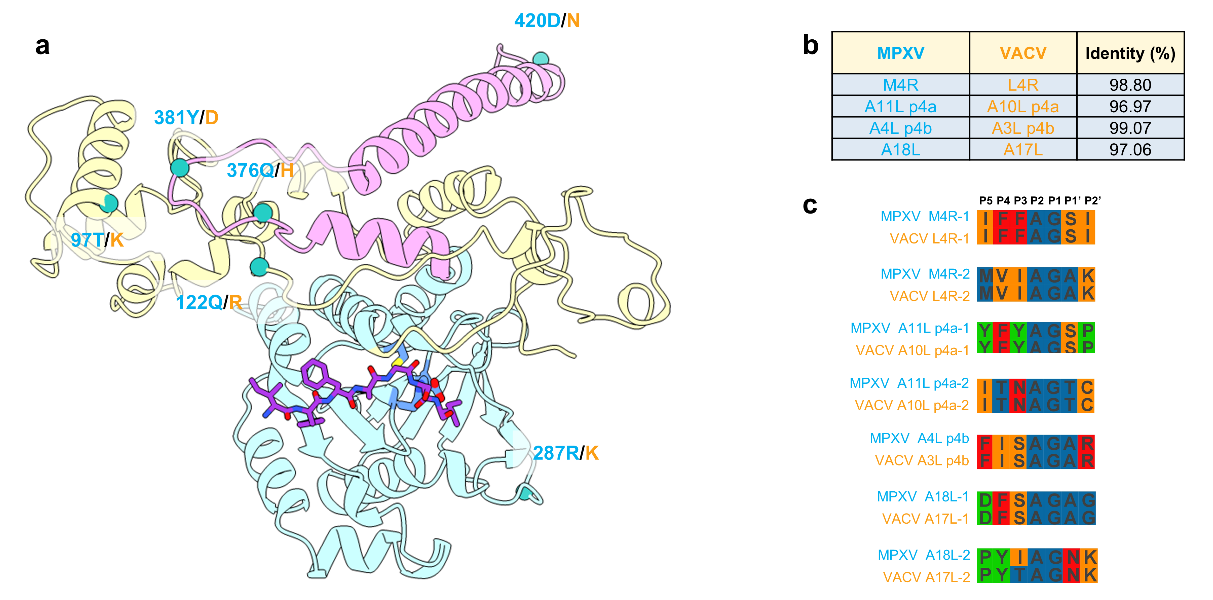


**Figure S8.** Sequence comparison between MPXV and VACV I7L proteases and their substrate proteins. (a) Cartoon representation of the modeled structure of MPXV I7L protease in complex with its substrate peptide by AlphaFold3. The N-terminal and C-terminal segments are shown in light yellow and pink, respectively, and the catalytic domain is displayed in pale cyan. The six amino acid differences between MPXV and VACV I7L are highlighted with cyan dots, with MPXV residues marked in light blue and VACV residues in orange. The substrate peptide is shown in purple sticks. (b) Sequence identities between the substrate proteins of MPXV and VACV I7L proteases. (c) Cleavage motifs (P2'–P5) in the substrate proteins of MPXV and VACV I7L proteases.


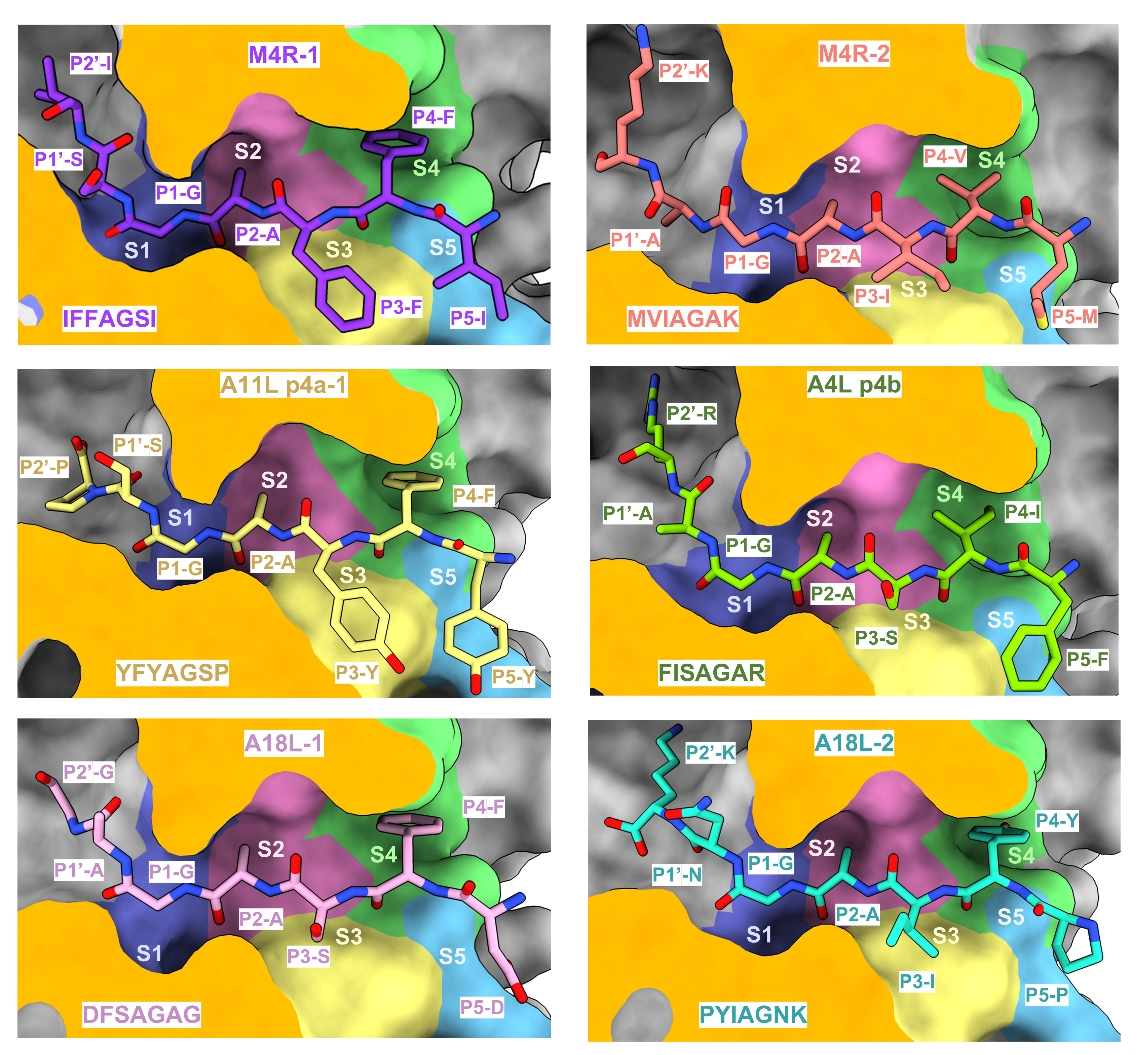


**Figure S9.** Molecular surface representation of the substrate peptides in complex with MPXV I7L protease predicted by AlphaFold3. The substrate peptides M4R-1, M4R-2, A11L p4a-1, A4L p4b, A18L-1, and A18L-2 are shown in purple, salmon, yellow, lime green, pink, and cyan sticks, respectively. The subsites S1-S5 are colored purple, pink, yellow, green, and sky blue, respectively.


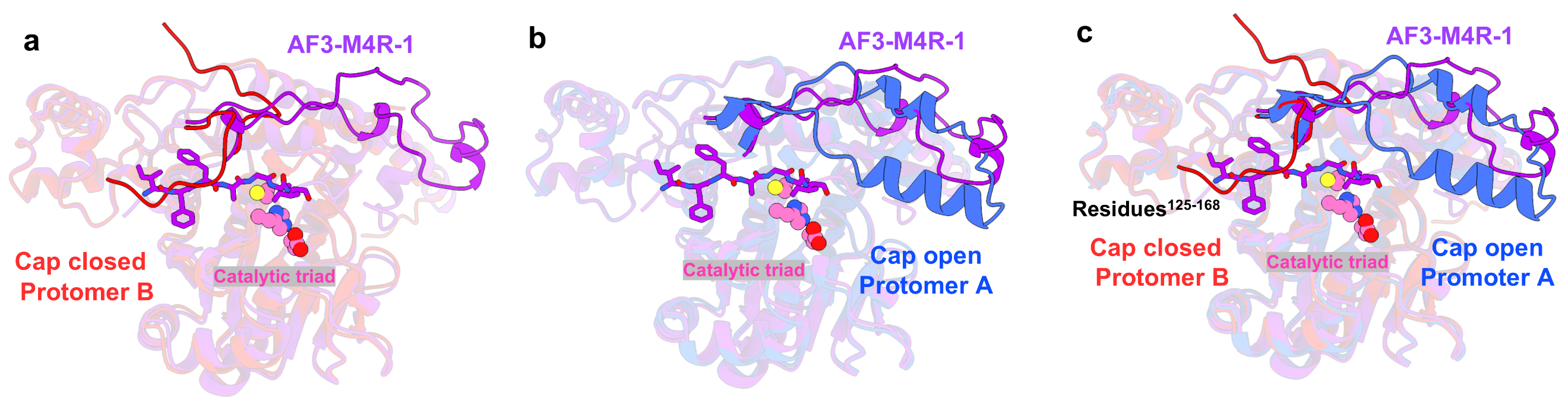


**Figure S10.** Conformational changes of the cap region in MPXV I7L protease revealed by crystal structures and AphaFold3. (a, b) Superimposition of the AlphaFold3-predicted MPXV I7L protease bound with the M4R-1 substrate with protomers B (a) and A (b) in the crystal structure of the C-tagged MPXV I7L protease, respectively. (c) Superimposition of the AlphaFold3-predicted MPXV I7L protease bound with the M4R-1 substrate with protomer A and B in the structure of the C-tagged MPXV I7L protease. The AlphaFold3-predicted MPXV I7L protease is shown as magenta cartoons. The M4R-1 substrate is shown as magenta sticks. Protomers A and B of the C-tagged MPXV I7L protease are shown as red and blue cartoons, respectively. The catalytic triad residues are depicted as pink spheres.


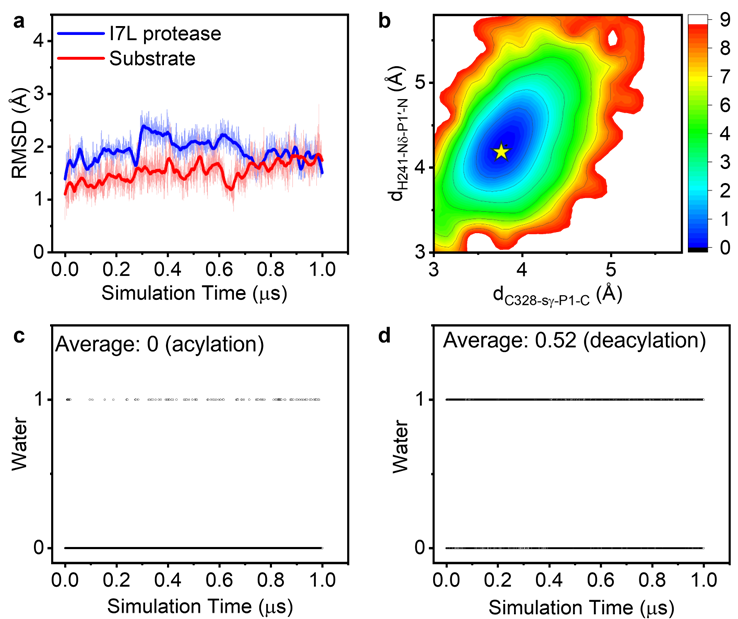


**Figure S11.** Structural features of the I7L protease-substrate complex contributing to the acylation and deacylation reactions. (a) Conventional MD trajectory of the non-covalent complex of MPXV I7L protease-substrate as indicated by the time series of the main chain root-mean-square deviations (RMSDs) for the protease (blue line) and the substrate (red line). (b) The FEL for the non-covalent complex of MPXV I7L protease-substrate along the distance between Cys328-Sγ and substrate’s P1-C atoms (d_C328-Sγ–P1-C_) and the distance between His241-Nε and substrate’s P1'-N atoms (d_H241-Nε–P1′-N_). The contours in the two-dimensional subspace are spaced at intervals of 1.0 kcal/mol. The yellow star indicates the location of the most populated state on the FEL. (c, d) The MD trajectory indicating the existence of a key water molecule in the deacylation but not in the acylation step of the proteolysis cycle.
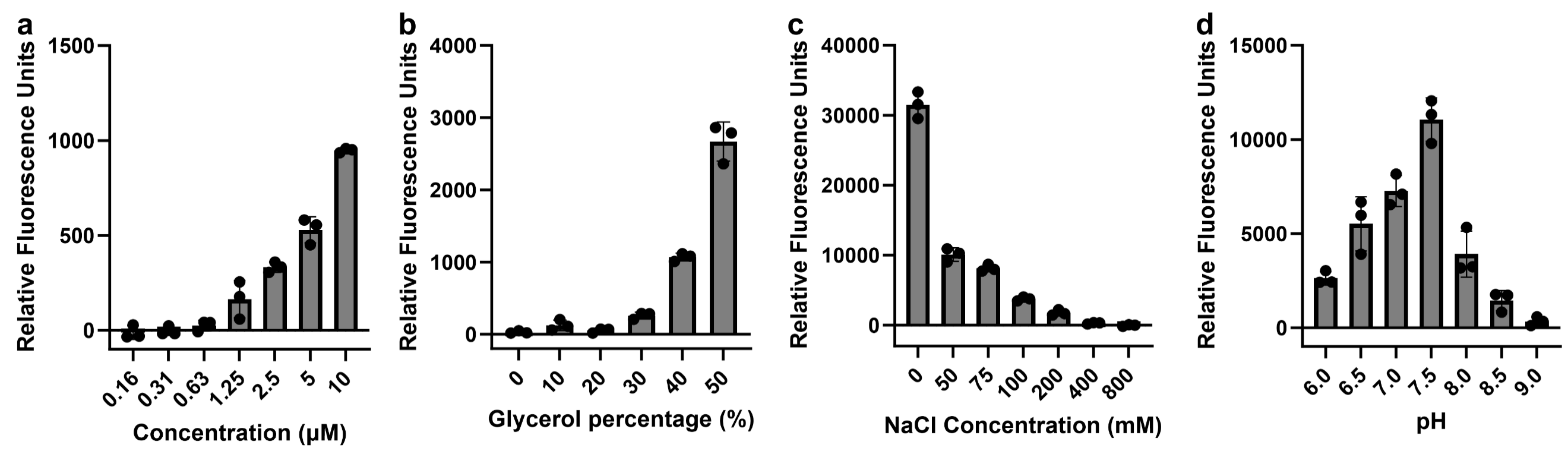


**Figure S12.** Effect of glycerol, sodium chloride (NaCl), and pH on the enzymatic activity of N-tagged I7L protease. (a) Fluorescence intensity generated by the cleavage of a fluorescent substrate at different concentrations of N-tagged I7L protease under physiological conditions (pH 7.5, 150 mM NaCl). (b) Fluorescence intensity generated by the cleavage of a fluorescent substrate by 200 nM N-tagged I7L protease at varying glycerol concentrations, at pH 7.5 and with 150 mM NaCl maintained constant. (c) Fluorescence intensity generated by the cleavage of a fluorescent substrate by 200 nM N-tagged I7L protease at varying NaCl concentrations, at pH 7.5 and with 50% glycerol maintained constant. (d) Fluorescence intensity generated by the cleavage of a fluorescent substrate by 200 nM N-tagged I7L protease at varying pH, with 150 mM NaCl and 50% glycerol maintained constant.


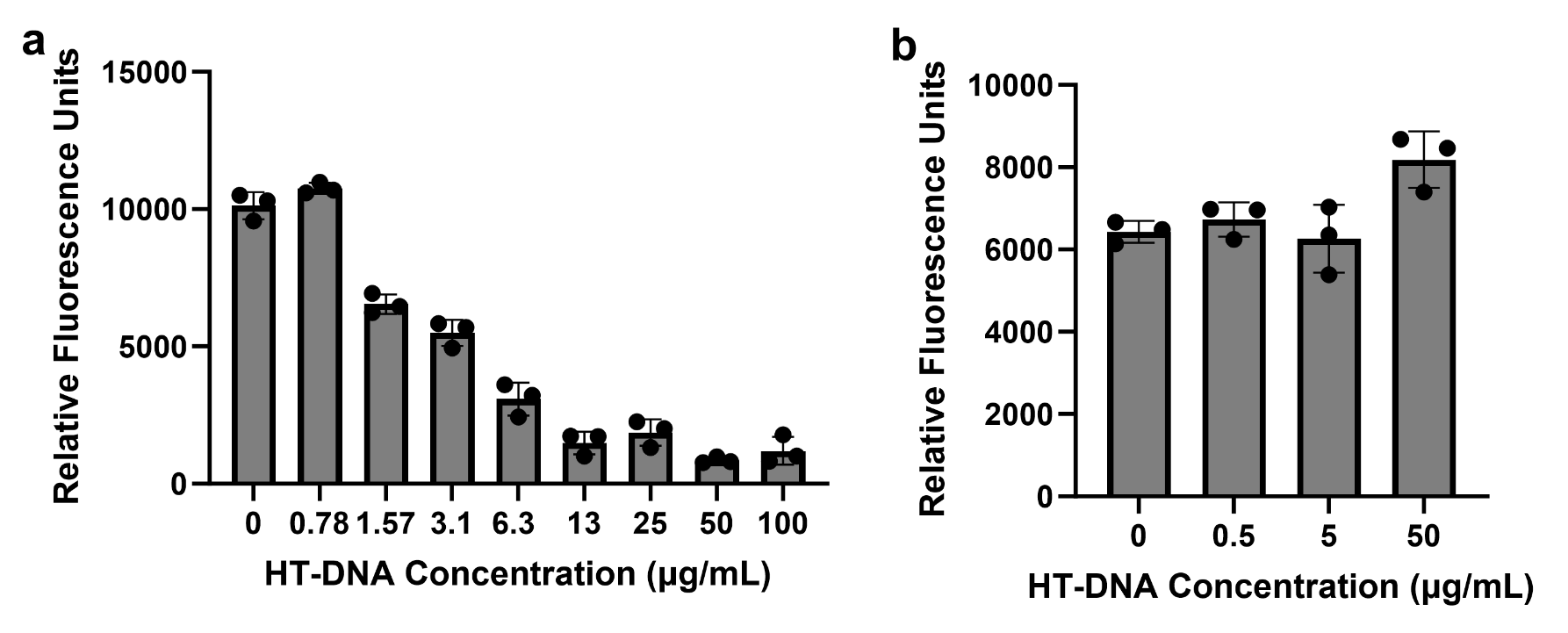


**Figure S13.** Effect of Herring Testes DNA (HT-DNA) on the enzymatic activity of N-tagged I7L protease and SARS-CoV-2 3CL^pro^. (a) Fluorescence intensity generated by the cleavage of a fluorescent substrate by 200 nM N-tagged I7L protease at different concentrations of HT-DNA, at pH 7.5, with 75 mM NaCl and 50% glycerol. (b) Fluorescence intensity generated by the cleavage of a fluorescent substrate by 50 nM SARS-CoV-2 3CL^pro^ at different concentrations of HT-DNA, at pH 7.3 and with 1 mM EDTA.


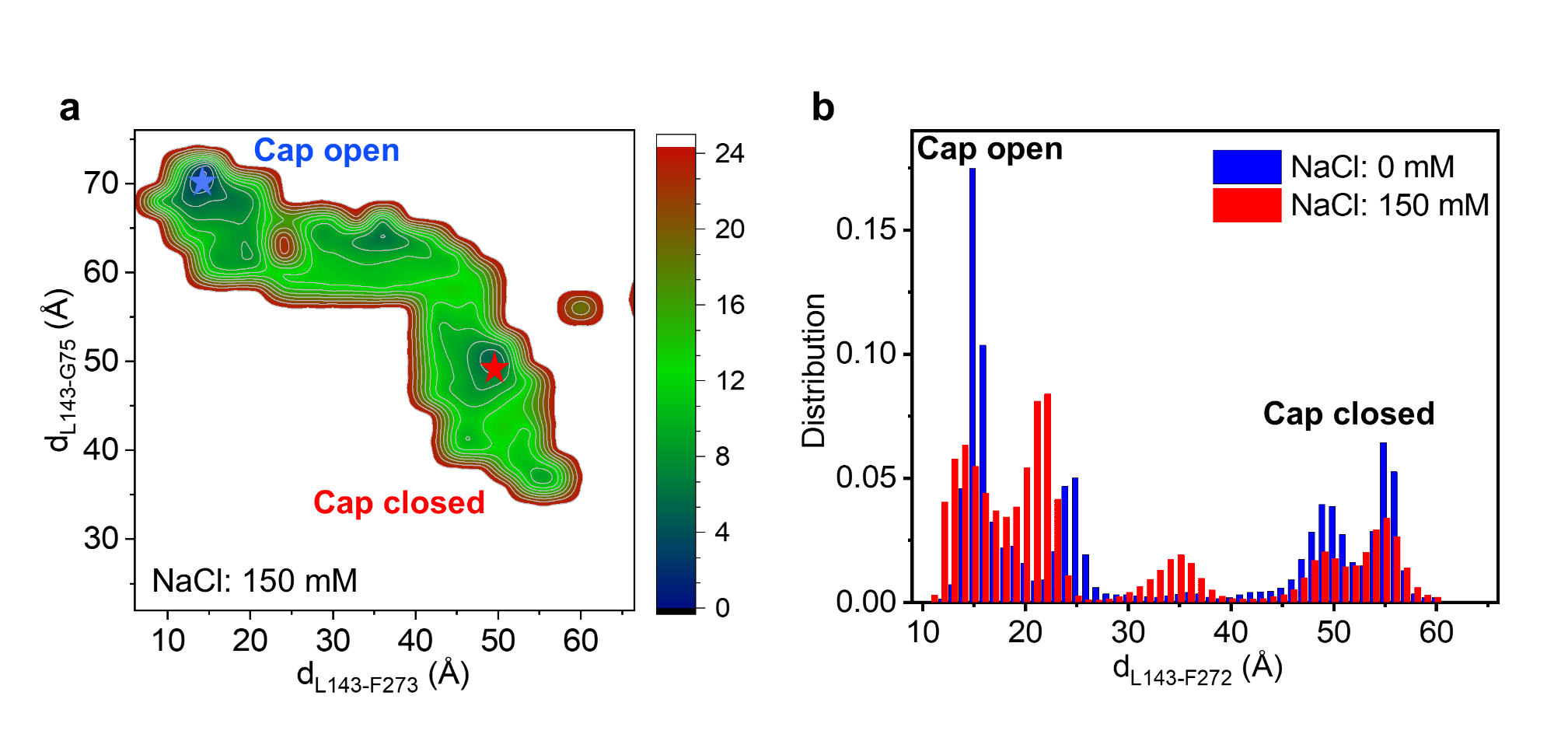


**Figure S14.** Effect of NaCl on the cap region conformation of MPXV I7L protease revealed by GaMD simulations. (a) The free energy landscape (FEL) calculated based on the MD trajectory shows the conformation distribution of the cap region in 150 mM NaCl solution. The FEL was drawn along two coordinates: the distances between residues from Leu143 to Gly75 in one protomer (y-axis) and to Phe273 in the other protomer (x-axis). The color gradient indicates the population density of the conformation, with blue and red representing the most and the least populated ones, respectively. The contours in the two-dimensional subspace are spaced at intervals of 1.0 kcal/mol. (b) The salt influence on the distribution of the protease’s conformation populations (with the focus on the cap region) in the simulations.


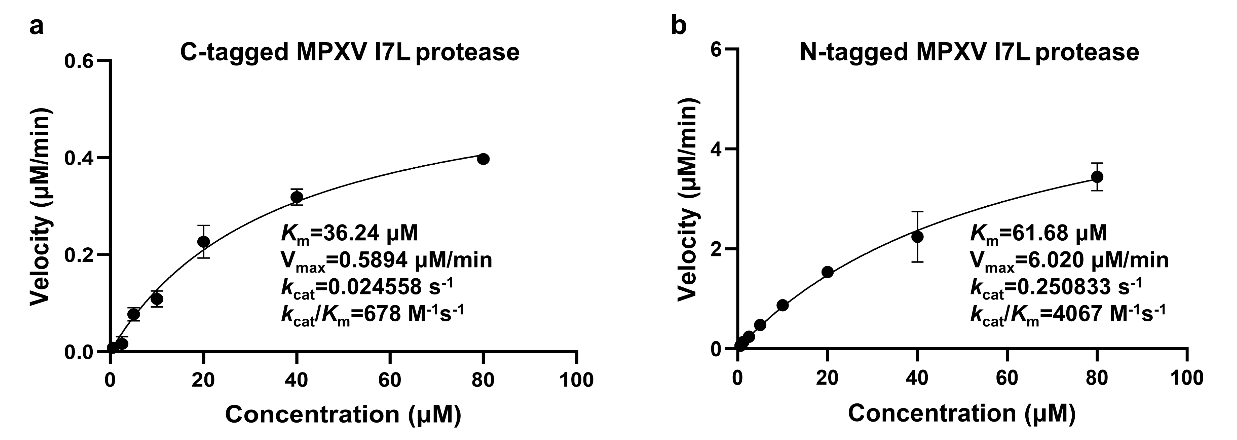


**Figure S15.** Determination of the catalytic efficiency of MPXV I7L proteases. (a, b) The curves of substrate cleavage rates by the C-tagged (a) or N-tagged (b) MPXV I7L protease versus substrate concentrations. Three independent experiments were conducted, and the data are presented as the mean ± SD of these three experiments.


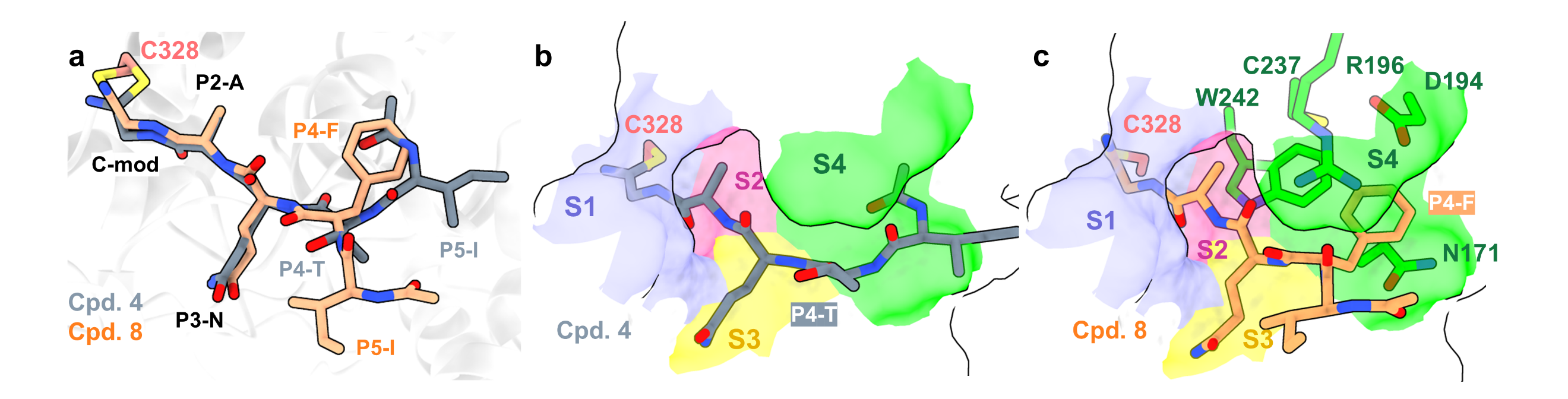


**Figure S16.** Covalent docking models of compounds **4** and **8** with MPXV I7L protease.
(a) Superimposed binding poses of compounds **4** (grey sticks) and **8** (light orange sticks) after covalently docked to the MPXV I7L protease, with the protein depicted in cartoon representation. Molecular surface representation of the MPXV I7L protease highlighting the binding subsites of compounds **4** (b) and **8** (c).


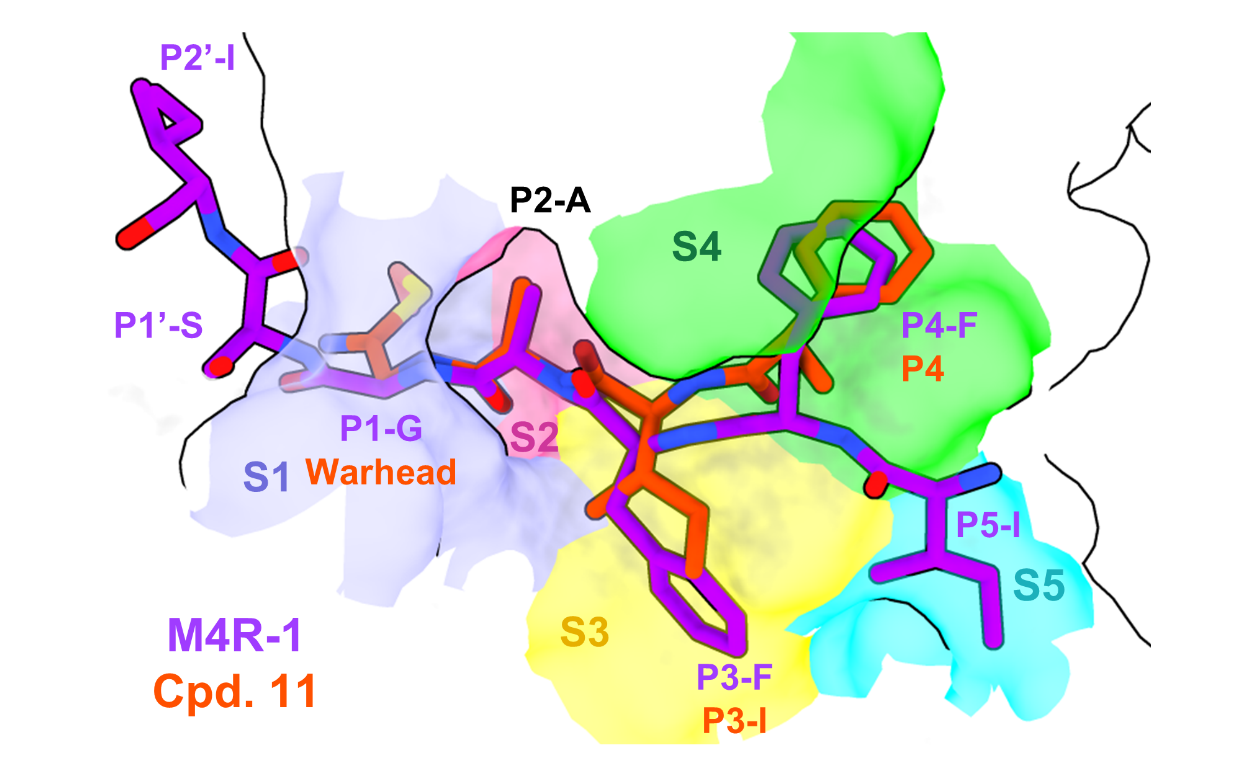


**Figure S17.** Structural comparison of the predicted binding modes of compound **11** (covalent docking model) and the substrate M4R-1 (AlphaFold3 prediction) with MPXV I7L protease. The substrate M4R-1 and compound **11** are depicted as sticks colored purple and red, respectively. The protease subsites (S1–S5) are colored as follows: S1 (purple), S2 (pink), S3 (yellow), S4 (green), and S5 (cyan).


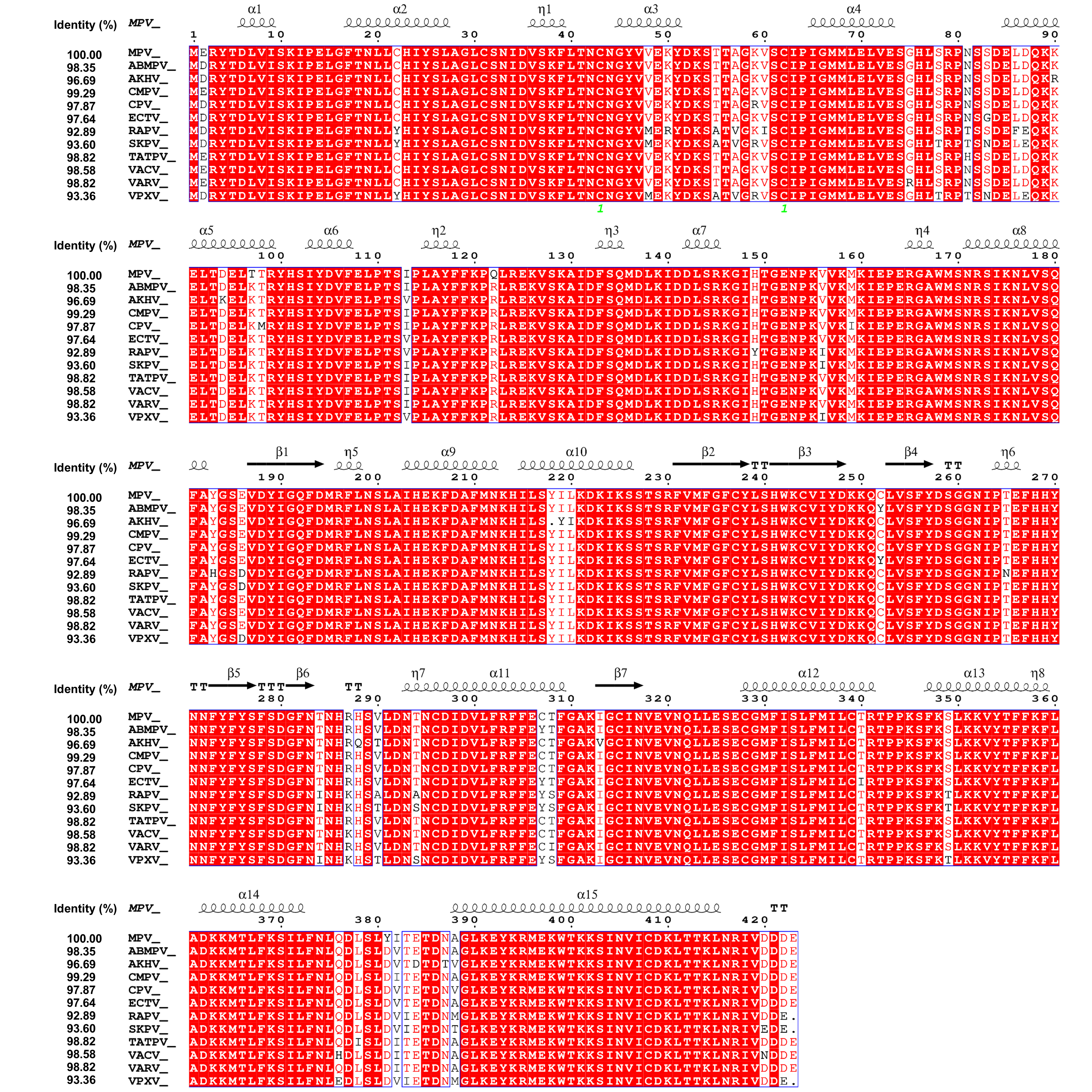


**Figure S18.** Sequence alignment of the I7L protease in twelve orthopoxviruses.


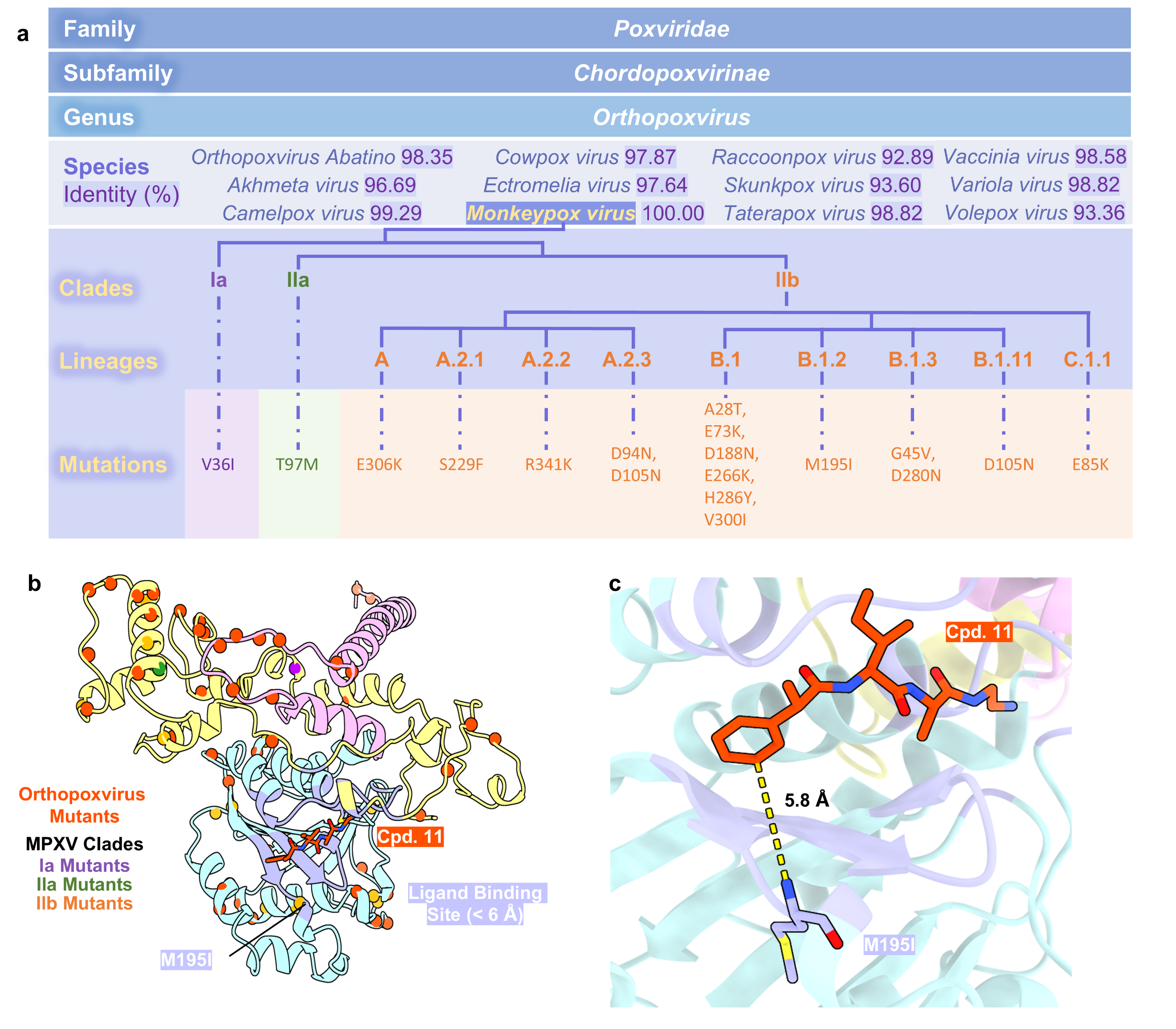


**Figure S19.** (a) Sequence identities between MPXV I7L protease and the proteases of other eleven orthopoxviruses, as well as mutations across different clades of monkeypox I7L protease. (b) Cartoon representation of the structure of MPXV I7L protease docked with compound **11**. The N-terminal segment is shown in light yellow, the C-terminal segment in pink, and the catalytic domain in pale cyan. Compound **11** is shown in red sticks. Amino acid differences between MPXV I7L protease and the corresponding I7L proteases of eleven other orthopoxviruses are marked with red dots. Mutations specific to clade Ⅰa, Ⅱa, and Ⅱb are highlighted in purple, green, and orange dots, respectively. (c) Close-up view of the mutated residue within 6 Å of compound **11**, shown in stick representation. Yellow dashed lines indicate a minimum distance between M195I and compound **11**.

**Table S1.** The Crystallography data collection and refinement statistics of C-tagged and N-tagged MPXV I7L protease.

| **PDB ID** | **9LIK** | **9LIL** | **9LIM** |
| --- | --- | --- | --- |
| Protein | C-tagged MPXV  I7L protease | N-tagged MPXV  I7L protease | N-tagged MPXV  I7L protease |
| The remaining sequence | C-terminal ENLYFQ | N-terminal GSG | N-terminal GSG |
| Space Group | *P*64 | *I*4 | *P*2_1_2_1_2_1_ |
| Cell Dimensions |  |  |  |
| *a*, *b*, *c* (Å) | 187.158, 187.158, 69.5086 | 94.912, 94.912, 114.097 | 42.992, 113.502, 193.144 |
| α, β, γ (°) | 90, 90, 120 | 90, 90, 90 | 90, 90, 90 |
| Wavelength (Å) | 0.97918 | 0.97918 | 0.97918 |
| Reflections (unique) | 41167 (3013) | 25443 (3711) | 42335 (6068) |
| Resolution Range (Å) | 44.95-2.64 | 67.11-2.21 | 97.86-2.32 |
| Highest-Resolution Shell (Å) | 2.70-2.64 | 2.29-2.21 | 2.37-2.32 |
| Redundancy | 20.2 (20.3) | 12.3 (12.4) | 11.1 (11.4) |
| I/σ (I) | 19.1 (1.8) | 7.2 (1.1) | 5.9 (1.0) |
| Highest-Resolution Shell CC_1/2_ | 0.731 | 0.397 | 0.342 |
| Completeness (%) | 99.50 (99.29) | 99.65 (98.42) | 99.69 (98.56) |
| Rwork/Rfree | 0.2424/0.2701 | 0.1978/0.2346 | 0.2174/0.2420 |
| **RMS Values** | | |  |
| Bond length (Å) | 0.006 | 0.003 | 0.002 |
| Bond angle (°) | 0.850 | 0.58 | 0.421 |
| **Numbers of Non-hydrogen Atoms** | | |  |
| Protein | 6351 | 3107 | 6044 |
| Water Oxygen | 10 | 112 | 199 |
| Others | 0 | 0 | 15 |
| Clashscore | 2.76 | 1.46 | 2.26 |
| MolProbity Score | 1.06 | 0.88 | 1.00 |
| **B-factor (Å^2^)** | | |  |
| Protein | 77.93 | 43.71 | 37.46 |
| Water Oxygen | 62.78 | 44.82 | 36.98 |
| **Ramachandran plot** | | |  |
| Favored (%) | 98.03 | 99.22 | 98.40 |
| Allowed (%) | 1.85 | 0.78 | 1.60 |
| Outliers (%) | 0.12 | 0 | 0 |

# Chemistry

**Scheme 1**

Scheme 1. Synthesis of compound **1-5, 7-9**

Reagents and conditions: (a) 2-Aminoacetonitrile hydrochloride, HATU, DIPEA, MeCN.

**Scheme 2**

Scheme 2. Synthesis of compound **6**

Reagents and conditions: (a) 2-Aminoacetonitrile hydrochloride, HATU, DIPEA, MeCN; (b) Pd(PPh_3_)_4_, PhSiH_3_, DCM.

**Scheme 3**

Scheme 3. Synthesis of compound **10, 11**

Reagents and conditions: (a) Boc-L-Alanine, HATU, DIPEA, MeCN; (b) 4M HCl in 1,4-dioxane, DCM; (c) Boc-L-Isoleucine, EDCI, HOBt, NMM, MeCN; (d) TFA, DCM; (e) RCOOH, EDCI, HOBt, NMM, MeCN.

**Characterization data (^1^H NMR and ^13^C NMR) of the synthesized compounds**

**(2S,3S)-2-acetamido-*N*-((S)-1-(((S)-1-(((S)-1-((cyanomethyl)amino)-1-oxopropan-2-yl)amino)-1-oxo-3-phenylpropan-2-yl)amino)-1-oxo-3-phenylpropan-2-yl)-3-methylpentanamide**

Compound **1**, white powder; ^1^H NMR (500 MHz, DMSO-*d*_6_) *δ* 8.48 (t, *J* = 5.6 Hz, 1H), 8.22 (d, *J* = 7.3 Hz, 1H), 8.02 (d, *J* = 8.0 Hz, 1H), 7.94 (d, *J* = 8.3 Hz, 1H), 7.79 (d, *J* = 8.7 Hz, 1H), 7.30 – 7.13 (m, 10H), 4.59 – 4.45 (m, 2H), 4.27 (dd, *J* = 14.3, 7.1 Hz, 1H), 4.15 (d, *J* = 5.9 Hz, 2H), 4.13 – 4.05 (m, 1H), 3.05 (dd, *J* = 14.1, 4.7 Hz, 1H), 2.93 (dd, *J* = 14.0, 4.0 Hz, 1H), 2.83 (m, 1H), 2.76 – 2.66 (m, 1H), 1.82 (s, 3H), 1.60 (d, *J* = 6.4 Hz, 1H), 1.32 – 1.17 (m, 4H), 1.03 – 0.93 (m, 1H), 0.75 (t, *J* = 7.4 Hz, 3H), 0.68 (d, *J* = 6.8 Hz, 3H). ^13^C NMR (126 MHz, DMSO-*d*_6_) *δ* 172.7, 170.9, 170.8, 170.5, 169.1, 137.7, 137.5, 129.2, 129.1, 128.0, 127.9, 126.2, 126.1, 117.5, 56.9, 53.6, 53.5, 48.0, 37.3, 36.2, 27.1, 24.2, 22.4, 17.8, 15.2, 10.9. HRMS (ESI) *m/z* calcd. for C_31_H_40_N_6_O_5_ [M + H]^+^ 577.3133, found 577.3133.

**(2S,3S)-2-((S)-2-((S)-2-acetamido-4-(methylthio)butanamido)-3-methylbutanamido)-*N*-((S)-1-((cyanomethyl)amino)-1-oxopropan-2-yl)-3-methylpentanamide**

Compound **2**, white powder; ^1^H NMR (500 MHz, DMSO-*d*_6_) *δ* 8.56 (t, *J* = 5.6 Hz, 1H), 8.11 – 8.05 (m, 2H), 7.88 – 7.78 (m, 2H), 4.38 (td, *J* = 8.2, 5.5 Hz, 1H), 4.25 (m, 1H), 4.20 – 4.11 (m, 4H), 2.45 – 2.38 (m, 2H), 2.03 (s, 3H), 1.99 – 1.91 (m, 1H), 1.89 – 1.80 (m, 4H), 1.73 (tt, *J* = 9.6, 6.3 Hz, 2H), 1.42 (m, 1H), 1.20 (d, *J* = 7.1 Hz, 3H), 1.14 – 1.03 (m, 1H), 0.86 – 0.78 (m, 12H). ^13^C NMR (126 MHz, DMSO-*d*_6_) *δ* 172.0, 170.6, 170.1, 170.0, 168.6, 116.9, 57.1, 55.9, 51.2, 47.3, 35.8, 31.4, 29.7, 28.9, 26.4, 23.6, 21.8, 18.6, 17.6, 17.1, 14.6, 14.0, 10.3. HRMS (ESI) *m/z* calcd. for C_23_H_40_N_6_O_5_S [M + H]^+^ 513.2854, found 513.2854.

**(S)-2-acetamido-*N*-((S)-1-(((S)-1-(((S)-1-((cyanomethyl)amino)-1-oxopropan-2-yl)amino)-3-(4-hydroxyphenyl)-1-oxopropan-2-yl)amino)-1-oxo-3-phenylpropan-2-yl)-3-(4-hydroxyphenyl)propanamide**

Compound **3**, white powder; ^1^H NMR (500 MHz, DMSO-*d*_6_) *δ* 8.44 (t, *J* = 5.4 Hz, 1H), 8.25 – 8.16 (m, 1H), 8.03 (d, *J* = 7.9 Hz, 1H), 7.95 (dd, *J* = 12.6, 8.3 Hz, 2H), 7.26 – 7.14 (m, 5H), 7.04 (d, *J* = 7.8 Hz, 2H), 6.97 (d, *J* = 8.0 Hz, 2H), 6.69 – 6.58 (m, 4H), 4.52 – 4.43 (m, 2H), 4.35 (dd, *J* = 12.1, 6.2 Hz, 2H), 4.30 – 4.24 (m, 1H), 4.14 (d, *J* = 5.5 Hz, 2H), 3.05 – 2.89 (m, 2H), 2.83 – 2.66 (m, 3H), 1.71 (s, 3H), 1.23 (d, *J* = 7.0 Hz, 3H). ^13^C NMR (126 MHz, DMSO-*d*_6_) 172.7, 171.4, 170.7, 169.0, 155.8, 155.6, 137.6, 130.1, 130.0, 129.2, 128.1, 127.9, 127.5, 126.2, 117.5, 114.9, 114.7, 54.0, 53.9, 53.7, 48.0, 37.3, 36.6, 36.4, 27.1, 22.4, 17.8. HRMS (ESI) *m/z* calcd. for C_34_H_38_N_6_O_7_ [M + H]^+^ 643.2875, found 643.2874.

**(S)-2-((2S,3R)-2-((2S,3S)-2-acetamido-3-methylpentanamido)-3-hydroxybutanamido)-*N*^1^-((S)-1-((cyanomethyl)amino)-1-oxopropan-2-yl)succinamide**

Compound **4**, white powder; ^1^H NMR (500 MHz, DMSO-*d*_6_) *δ* 8.56 (t, *J* = 5.6 Hz, 1H), 8.15 (d, *J* = 7.2 Hz, 1H), 7.97 (dd, *J* = 18.4, 8.2 Hz, 2H), 7.75 (d, *J* = 8.1 Hz, 1H), 7.52 (s, 1H), 7.05 (s, 1H), 4.98 (s, 1H), 4.54 (q, *J* = 6.7 Hz, 1H), 4.24 – 4.17 (m, 3H), 4.12 – 4.08 (m, 2H), 4.02 – 3.95 (m, 1H), 2.66 – 2.55 (m, 2H), 1.87 (s, 3H), 1.73 (dd, *J* = 16.3, 10.1 Hz, 1H), 1.47 – 1.39 (m, 1H), 1.23 (d, *J* = 7.2 Hz, 3H), 1.15 – 1.07 (m, 1H), 1.03 (d, *J* = 6.3 Hz, 3H), 0.88 – 0.78 (m, 6H). ^13^C NMR (126 MHz, DMSO-*d*_6_) *δ* 172.1, 171.4, 170.8, 169.8, 169.1, 168.7, 116.8, 65.9, 57.3, 56.4, 49.0, 47.7, 36.3, 35.5, 26.5, 23.8, 21.8, 18.7, 16.7, 14.8, 10.3. HRMS (ESI) *m/z* calcd. for C_21_H_35_N_7_O_7_ [M + H]^+^ 498.2671, found 498.2670.

**(2S,3S)-2-((S)-2-acetamido-3-phenylpropanamido)-*N*-((S)-1-(((S)-1-((cyanomethyl)amino)-1-oxopropan-2-yl)amino)-3-hydroxy-1-oxopropan-2-yl)-3-methylpentanamide**

Compound **5**, white powder; ^1^H NMR (500 MHz, DMSO-*d*_6_) *δ* 8.53 (t, *J* = 5.6 Hz, 1H), 8.14 (d, *J* = 7.3 Hz, 1H), 8.09 (d, *J* = 8.4 Hz, 1H), 8.00 (d, *J* = 7.4 Hz, 1H), 7.92 (d, *J* = 8.7 Hz, 1H), 7.28 – 7.24 (m, 4H), 7.21 – 7.16 (m, 1H), 4.56 (td, *J* = 9.9, 4.2 Hz, 1H), 4.36 – 4.20 (m, 3H), 4.13 (dd, *J* = 5.6, 1.7 Hz, 2H), 3.68 – 3.56 (m, 3H), 3.04 – 2.96 (m, 1H), 2.72 (dd, *J* = 13.9, 10.2 Hz, 1H), 1.79 – 1.71 (m, 4H), 1.48 – 1.39 (m, 1H), 1.23 (d, *J* = 7.2 Hz, 3H), 1.13 – 1.03 (m, 1H), 0.86 – 0.80 (m, 6H). ^13^C NMR (126 MHz, DMSO-*d*_6_) *δ* 172.7, 171.3, 170.9, 169.7, 169.1, 138.0, 129.1, 128.0, 126.1, 117.4, 61.6, 56.8, 54.7, 53.7, 48.1, 37.2, 36.8, 27.1, 24.2, 22.4, 17.7, 15.2, 11.1. HRMS (ESI) *m/z* calcd. for C_25_H_36_N_6_O_6_ [M + H]^+^ 517.2769, found 517.2767.

**(4S,7S,10S,13S)-13-acetamido-10-benzyl-1-cyano-7-(hydroxymethyl)-4-methyl-3,6,9,12-tetraoxo-2,5,8,11-tetraazapentadecan-15-oic acid**

Compound **6**, white powder; ^1^H NMR (500 MHz, DMSO-*d*_6_) *δ* 12.27 (s, 1H), 8.50 (t, *J* = 5.5 Hz, 1H), 8.16 (dd, *J* = 7.5, 3.1 Hz, 2H), 8.10 (d, *J* = 7.6 Hz, 1H), 7.81 (d, *J* = 8.0 Hz, 1H), 7.27 – 7.15 (m, 5H), 4.55 – 4.48 (m, 2H), 4.35 – 4.26 (m, 2H), 4.14 (d, *J* = 5.6 Hz, 2H), 3.67 – 3.56 (m, 3H), 3.05 (dd, *J* = 13.8, 4.0 Hz, 1H), 2.81 (dd, *J* = 13.8, 9.1 Hz, 1H), 2.60 (dd, *J* = 16.6, 5.5 Hz, 1H), 2.40 – 2.34 (m, 1H), 1.80 (s, 3H), 1.26 (d, *J* = 7.1 Hz, 3H). ^13^C NMR (126 MHz, DMSO-*d*_6_) *δ* 173.2, 172.2, 171.3, 171.1, 170.2, 170.0, 138.0, 129.8, 128.4, 126.7, 117.9, 62.1, 55.4, 54.2, 49.9, 48.6, 37.7, 36.2, 27.6, 22.9, 18.1. HRMS (ESI) *m/z* calcd. for C_23_H_30_N_6_O_8_ [M + H]^+^ 519.2198, found 519.2200.

**(S)-1-acetyl-*N*-((S)-1-(((2S,3S)-1-(((S)-1-((cyanomethyl)amino)-1-oxopropan-2-yl)amino)-3-methyl-1-oxopentan-2-yl)amino)-3-(4-hydroxyphenyl)-1-oxopropan-2-yl)pyrrolidine-2-carboxamide**

Compound **7**, white powder; ^1^H NMR (500 MHz, DMSO-*d*_6_) *δ* 8.62 – 8.48 (m, 1H), 8.25 – 8.06 (m, 1H), 7.83 – 7.59 (m, 2H), 7.06 – 6.95 (m, 2H), 6.62 (dd, *J* = 8.5, 2.7 Hz, 2H), 4.60 – 4.54 (m, 1H), 4.44 – 4.39 (m, 1H), 4.29 – 4.21 (m, 2H), 4.14 (dd, *J* = 5.6, 2.8 Hz, 2H), 3.55 – 3.39 (m, 1H), 3.35 – 3.24 (m, 1H), 2.99 – 2.89 (m, 1H), 2.76 – 2.66 (m, 1H), 2.14 – 2.04 (m, 1H), 2.00 – 1.87 (m, 2H), 1.82 – 1.63 (m, 4H), 1.60 – 1.52 (m, 2H), 1.50 – 1.37 (m, 1H), 1.22 (dd, *J* = 7.1, 1.9 Hz, 3H), 1.13 – 1.04 (m, 1H), 0.88 – 0.78 (m, 6H). ^13^C NMR (126 MHz, DMSO-*d*_6_) *δ* 172.0, 170.9, 170.4, 170.1, 169.9, 169.9, 168.5, 168.0, 155.1, 155.1, 129.4, 129.3, 127.2, 127.2, 116.9, 114.1, 59.7, 58.7, 56.1, 55.8, 53.3, 47.3, 47.2, 46.9, 45.5, 36.3, 35.8, 35.6, 35.2, 30.9, 28.4, 26.4, 23.6, 23.5, 23.4, 21.7, 21.5, 21.2, 17.0, 17.0, 14.6, 10.3. HRMS (ESI) *m/z* calcd. for C_27_H_38_N_6_O_6_ [M + H]^+^ 543.2926, found 543.2928.

**(S)-2-((S)-2-((2S,3S)-2-acetamido-3-methylpentanamido)-3-phenylpropanamido)-*N*^1^-((S)-1-((cyanomethyl)amino)-1-oxopropan-2-yl)succinamide**

Compound **8**, white powder; ^1^H NMR (500 MHz, DMSO-*d*_6_) *δ* 8.61 (t, *J* = 5.6 Hz, 1H), 8.22 (d, *J* = 7.3 Hz, 1H), 8.14 (d, *J* = 7.7 Hz, 1H), 7.98 (d, *J* = 8.2 Hz, 1H), 7.81 (d, *J* = 8.7 Hz, 1H), 7.56 (s, 1H), 7.26 – 7.20 (m, 4H), 7.19 – 7.15 (m, 1H), 7.08 (s, 1H), 4.57 – 4.48 (m, 2H), 4.21 (p, *J* = 7.2 Hz, 1H), 4.16 – 4.04 (m, 3H), 3.01 (dd, *J* = 13.9, 4.4 Hz, 1H), 2.79 (dd, *J* = 13.9, 9.6 Hz, 1H), 2.65 – 2.56 (m, 1H), 2.46 (d, *J* = 5.9 Hz, 1H), 1.83 (s, 3H), 1.62 (td, *J* = 10.3, 3.4 Hz, 1H), 1.35 – 1.28 (m, 1H), 1.24 (d, *J* = 7.3 Hz, 3H), 1.07 – 0.96 (m, 1H), 0.80 – 0.68 (m, 6H). ^13^C NMR (126 MHz, DMSO-*d*_6_) *δ* 172.1, 171.4, 170.4, 170.2, 169.8, 168.6, 137.0, 128.5, 127.3, 125.5, 116.8, 56.3, 53.0, 49.0, 47.7, 36.7, 36.4, 35.5, 26.5, 23.6, 21.8, 16.7, 14.6, 10.3. HRMS (ESI) *m/z* calcd. for C_26_H_37_N_7_O_6_ [M + H]^+^ 544.2878, found 544.2878.

**(2S,3S)-2-((S)-2-acetamido-3-methylbutanamido)-*N*-((S)-1-((cyanomethyl)amino)-1-oxopropan-2-yl)-3-methylpentanamide**

Compound **9**, white powder; ^1^H NMR (500 MHz, DMSO-*d*_6_) *δ* 8.57 (t, *J* = 5.6 Hz, 1H), 8.07 (d, *J* = 7.0 Hz, 1H), 7.89 (d, *J* = 8.8 Hz, 1H), 7.75 (d, *J* = 8.6 Hz, 1H), 4.23 (t, *J* = 7.1 Hz, 1H), 4.20 – 4.15 (m, 2H), 4.13 (d, *J* = 5.7 Hz, 2H), 2.00 – 1.91 (m, 1H), 1.87 (s, 3H), 1.72 (td, *J* = 10.1, 3.2 Hz, 1H), 1.47 – 1.39 (m, 1H), 1.20 (d, *J* = 7.1 Hz, 3H), 1.13 – 1.02 (m, 1H), 0.87 – 0.78 (m, 12H). ^13^C NMR (126 MHz, DMSO-*d*_6_) *δ* 173.1, 171.5, 171.1, 169.7, 118.0, 58.2, 57.1, 48.4, 37.0, 30.7, 27.5, 24.7, 23.0, 19.7, 18.6, 18.2, 15.7, 11.5. HRMS (ESI) *m/z* calcd. for C_18_H_31_N_5_O_4_ [M + H]^+^ 382.2449, found 382.2449.

**(2S,3S)-*N*-((S)-1-((cyanomethyl)amino)-1-oxopropan-2-yl)-3-methyl-2-(2-phenylacetamido)pentanamide**

Compound **10**, white powder; ^1^H NMR (500 MHz, DMSO-*d*_6_) *δ* 8.54 (t, *J* = 5.6 Hz, 1H), 8.17 (d, *J* = 7.1 Hz, 1H), 8.10 (d, *J* = 8.8 Hz, 1H), 7.32 – 7.24 (m, 4H), 7.23 – 7.18 (m, 1H), 4.27 – 4.16 (m, 2H), 4.12 (d, *J* = 5.6 Hz, 2H), 3.53 (d, *J* = 13.8 Hz, 1H), 3.46 (d, *J* = 13.8 Hz, 1H), 1.72 (td, *J* = 10.1, 3.4 Hz, 1H), 1.45 – 1.37 (m, 1H), 1.20 (d, *J* = 7.1 Hz, 3H), 1.13 – 1.02 (m, 1H), 0.85 – 0.76 (m, 6H). ^13^C NMR (126 MHz, DMSO-*d*_6_) *δ* 173.2, 171.4, 170.6, 137.1, 129.5, 128.6, 126.7, 118.0, 57.0, 48.4, 42.5, 37.2, 27.5, 24.7, 18.1, 15.8, 11.4. HRMS (ESI) *m/z* calcd. for C_19_H_26_N_4_O_3_ [M + H]^+^ 359.2078, found 359.2077.

**(2S,3S)-*N*-((S)-1-((cyanomethyl)amino)-1-oxopropan-2-yl)-3-methyl-2-((S)-2-phenylpropanamido)pentanamide**

Compound **11**, white powder; ^1^H NMR (500 MHz, DMSO-*d*_6_) *δ* 8.52 (t, *J* = 5.6 Hz, 1H), 8.06 (d, *J* = 7.0 Hz, 1H), 8.00 (d, *J* = 8.9 Hz, 1H), 7.35 – 7.25 (m, 4H), 7.24 – 7.17 (m, 1H), 4.23 – 4.14 (m, 2H), 4.10 (d, *J* = 5.8 Hz, 2H), 3.81 (q, *J* = 7.0 Hz, 1H), 1.73 (td, *J* = 10.2, 3.4 Hz, 1H), 1.48 – 1.41 (m, 1H), 1.32 (d, *J* = 7.1 Hz, 3H), 1.14 (d, *J* = 7.1 Hz, 3H), 1.12 – 1.06 (m, 1H), 0.89 – 0.79 (m, 6H). ^13^C NMR (126 MHz, DMSO-*d*_6_) *δ* 172.7, 172.0, 170.1, 141.4, 127.4, 126.7, 125.7, 116.8, 55.7, 47.2, 43.7, 36.1, 26.3, 23.6, 18.2, 16.9, 14.6, 10.3. HRMS (ESI) *m/z* calcd. for C_20_H_28_N_4_O_3_ [M + H]^+^ 373.2234, found 373.2236.

**Spectra of the compounds**

^1^H NMR spectrum of compound **1** (500 MHz, DMSO-*d*_6_)


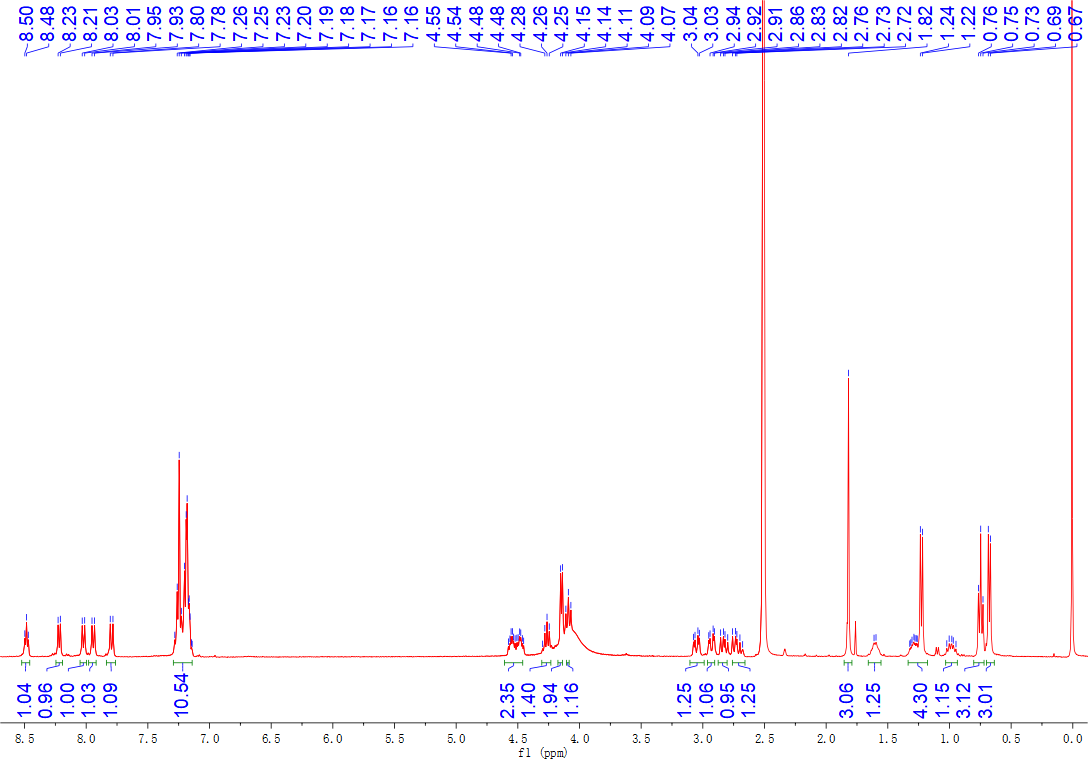


^13^C NMR spectrum of compound **1** (126 MHz, DMSO-*d*_6_)


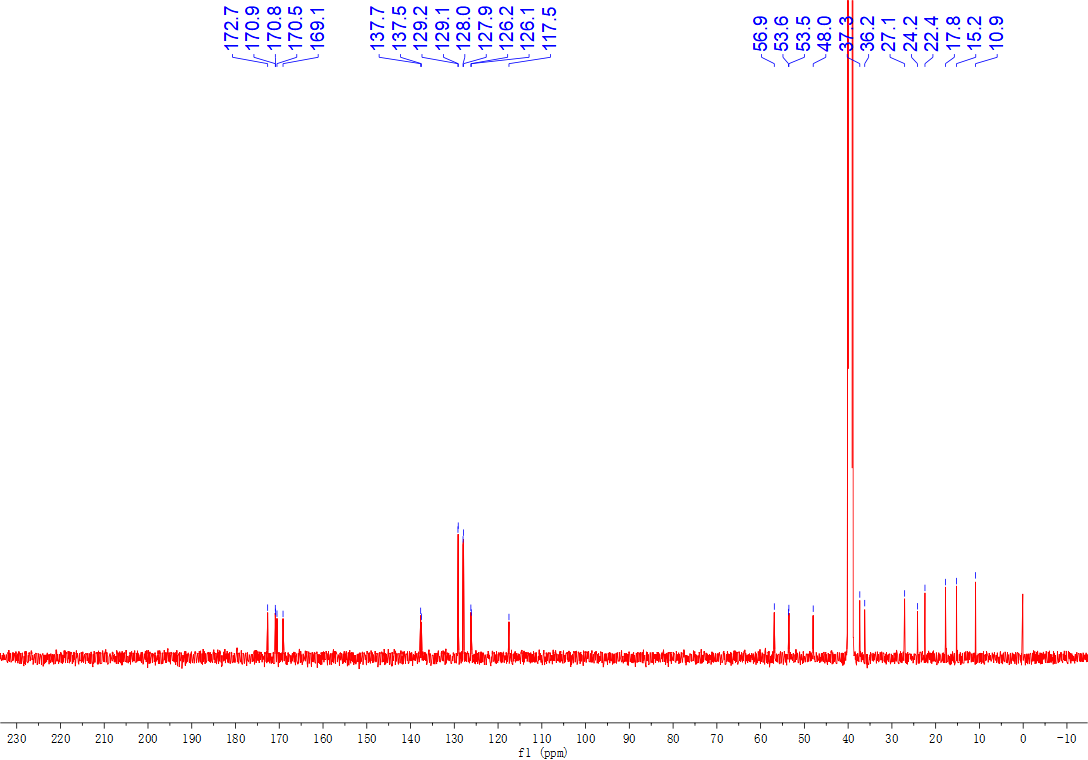


Copy of ESI-HR spectra of compound **1**


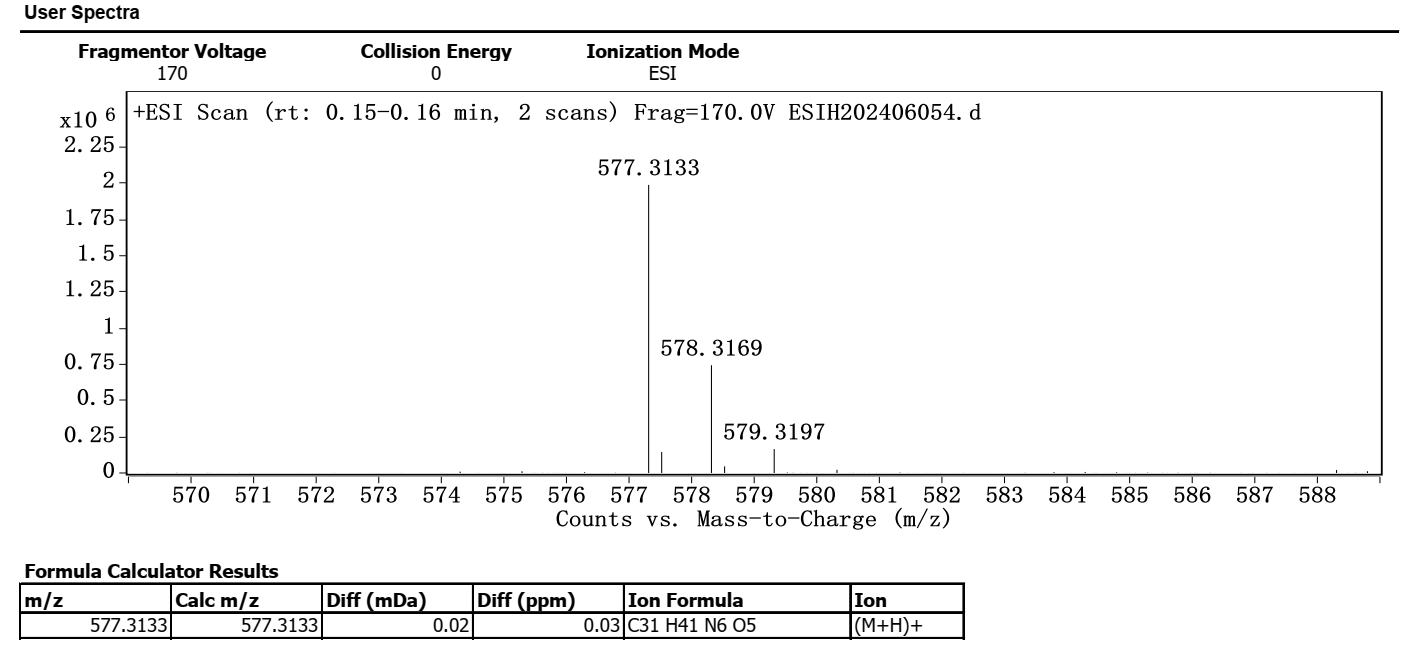


Copy HPLC spectra of compound **1**


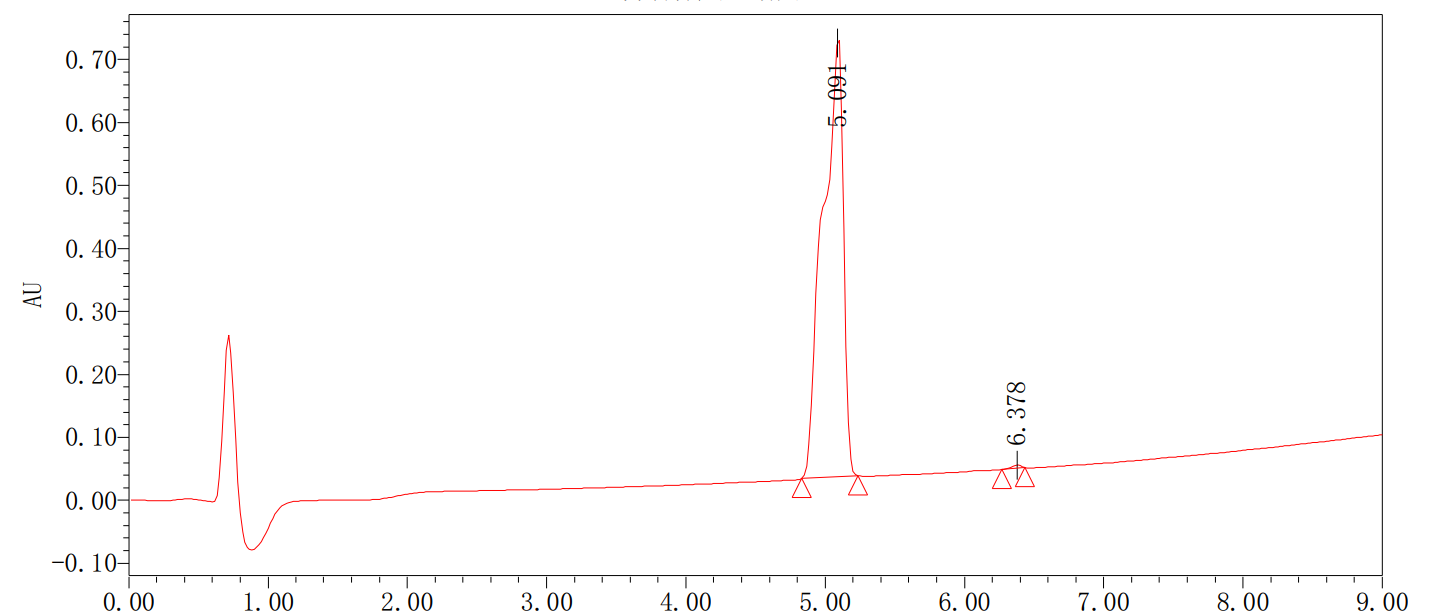


Peak result

|  | Retention time (min) | %Area |
| --- | --- | --- |
| 1 | 5.091 | 99.70 |
| 2 | 6.378 | 0.30 |

^1^H NMR spectrum of compound **2** (500 MHz, DMSO-*d*_6_)


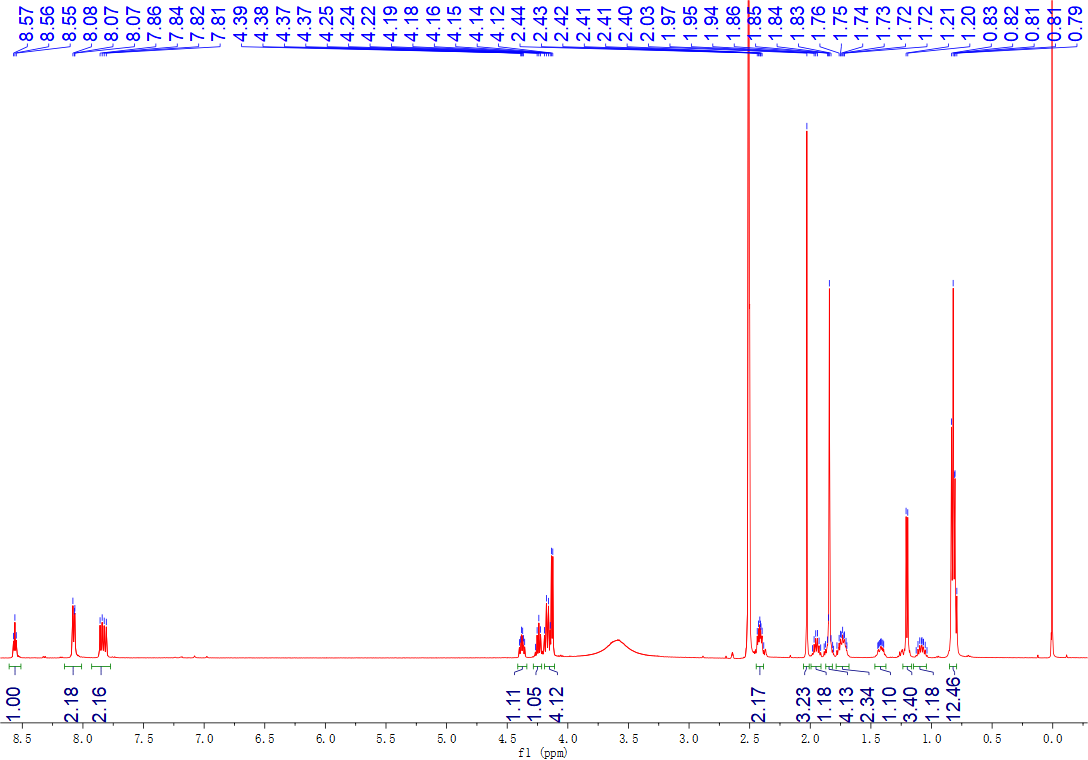


^13^C NMR spectrum of compound **2** (126 MHz, DMSO-*d*_6_)


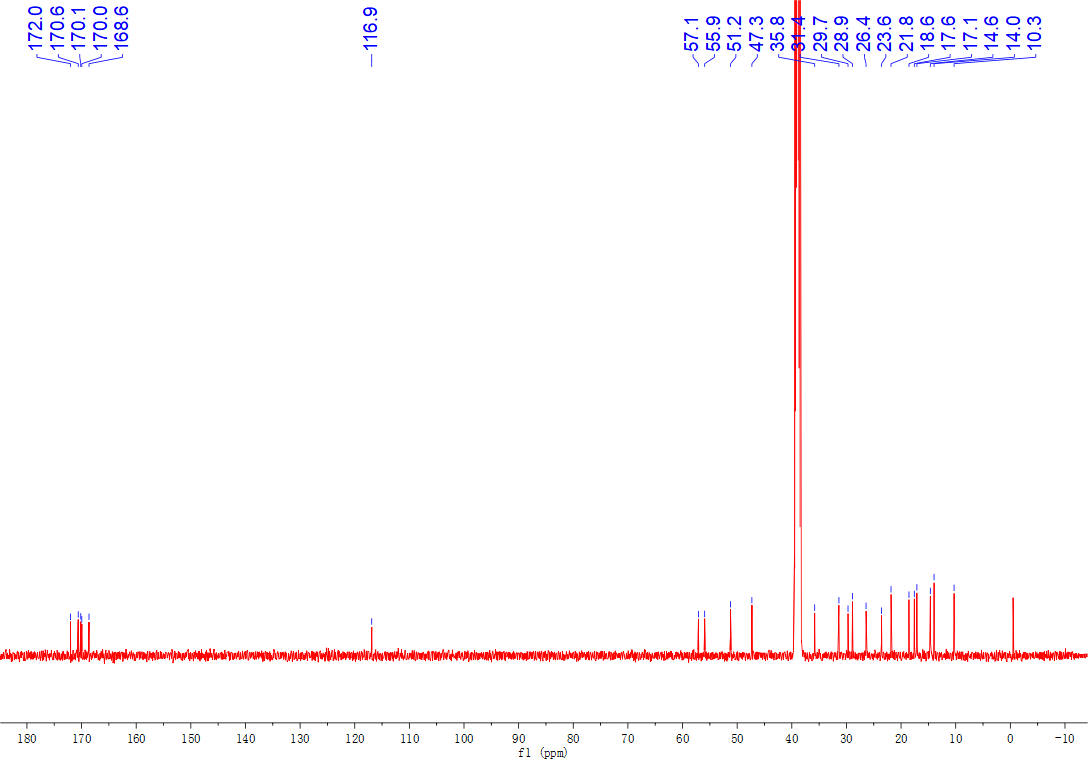


Copy of ESI-HR spectra of compound **2**


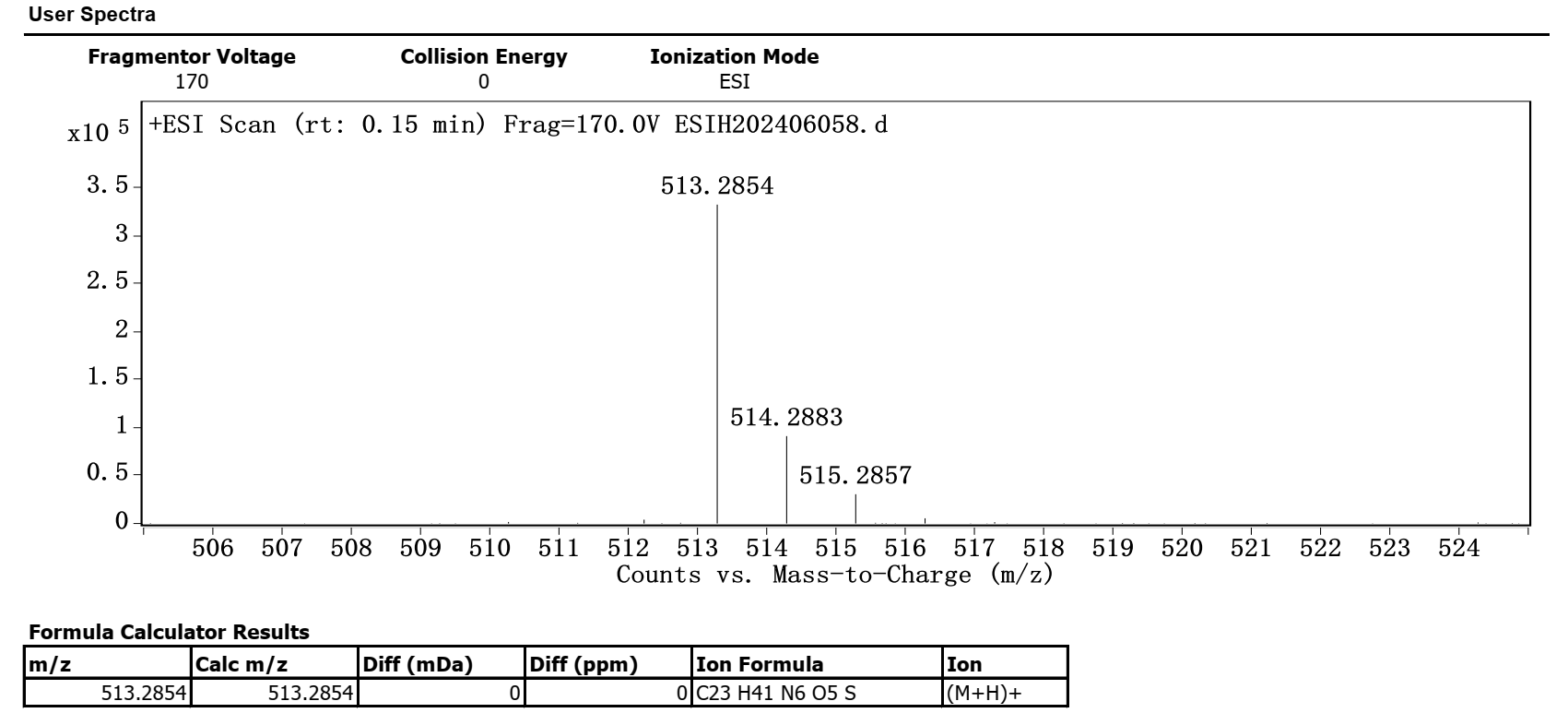


Copy HPLC spectra of compound **2**


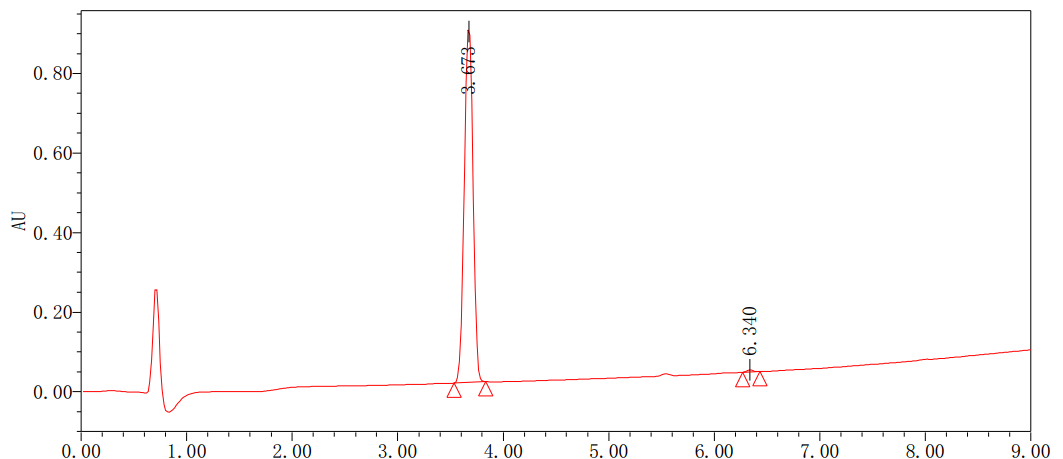


Peak result

|  | Retention time (min) | %Area |
| --- | --- | --- |
| 1 | 3.673 | 99.57 |
| 2 | 6.340 | 0.43 |

^1^H NMR spectrum of compound **3** (500 MHz, DMSO-*d*_6_)


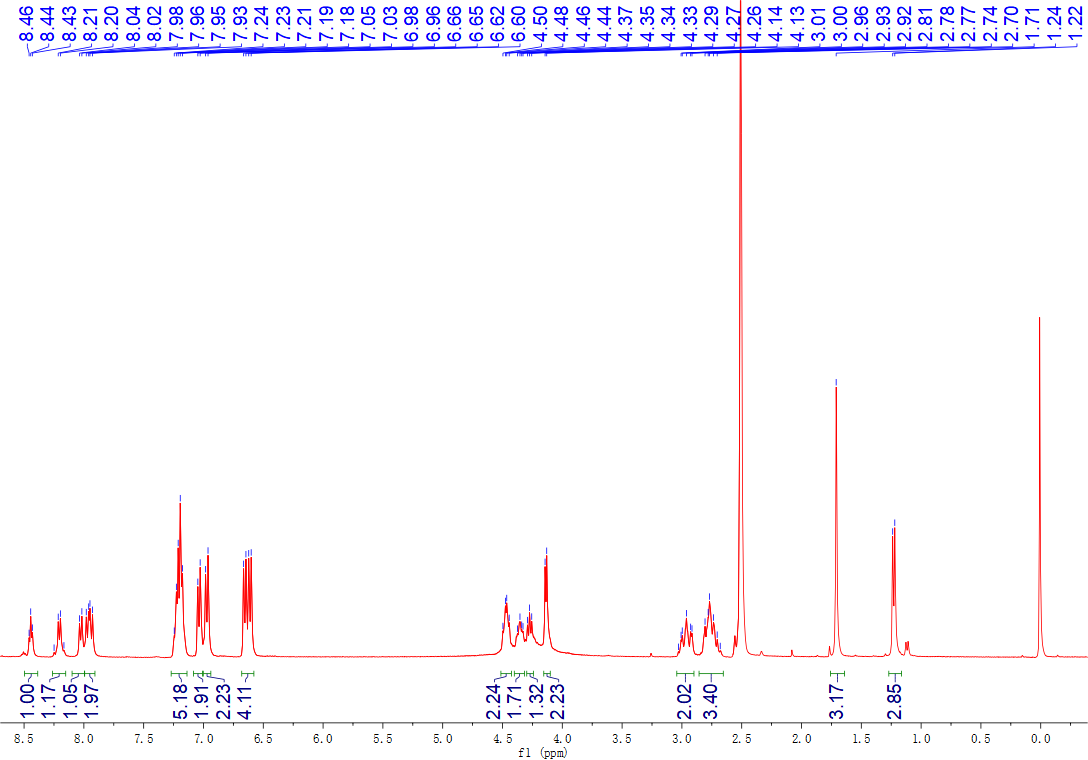


^13^C NMR spectrum of compound **3** (126 MHz, DMSO-*d*_6_)


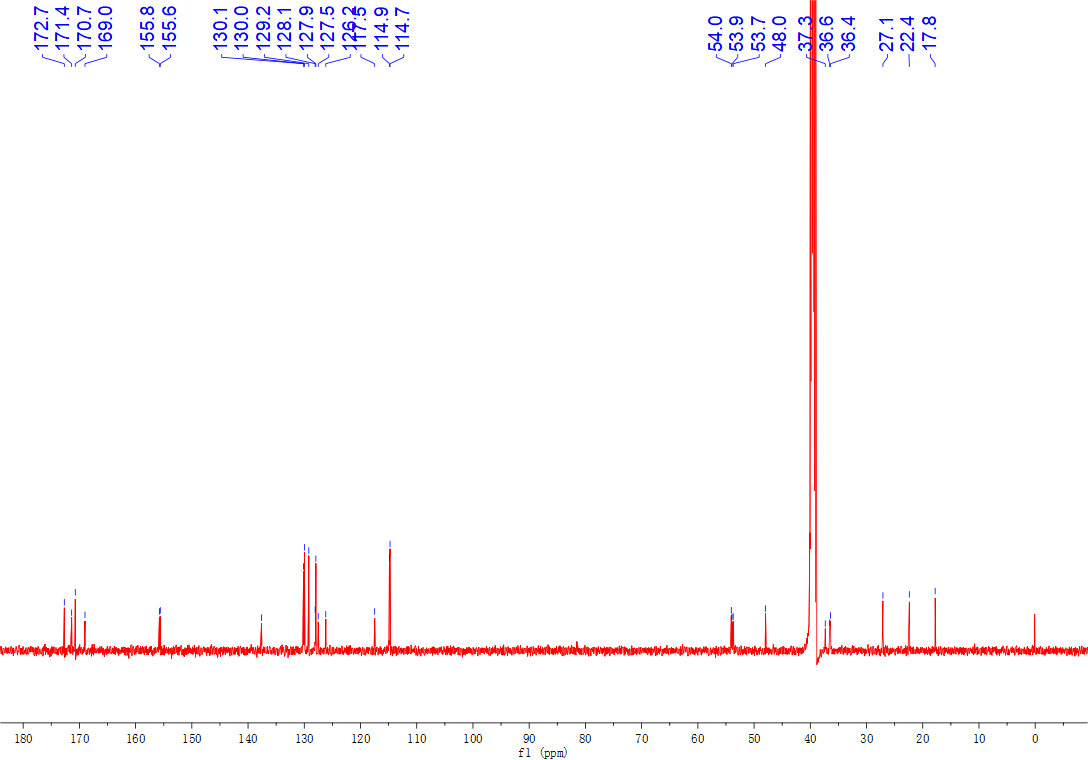


Copy of ESI-HR spectra of compound **3**


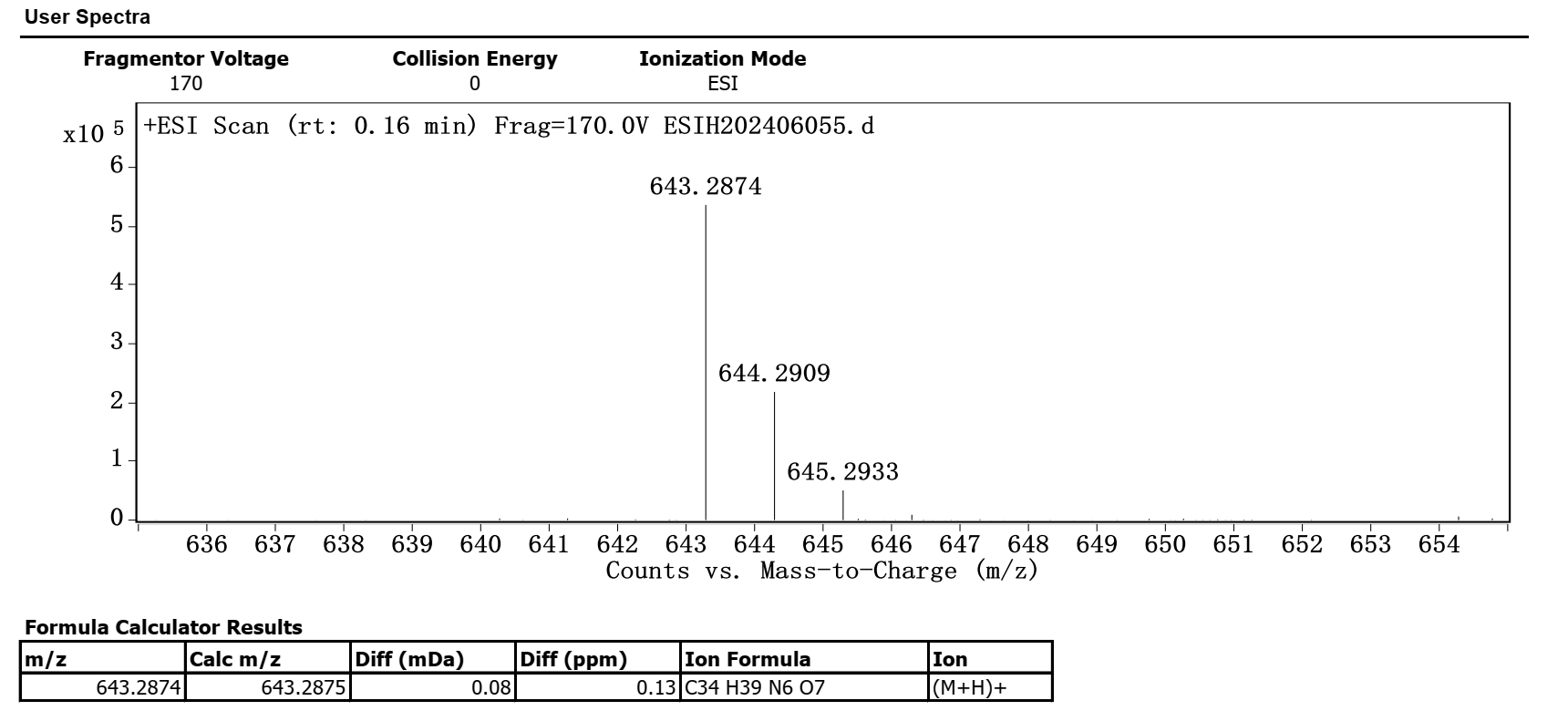


Copy HPLC spectra of compound **3**


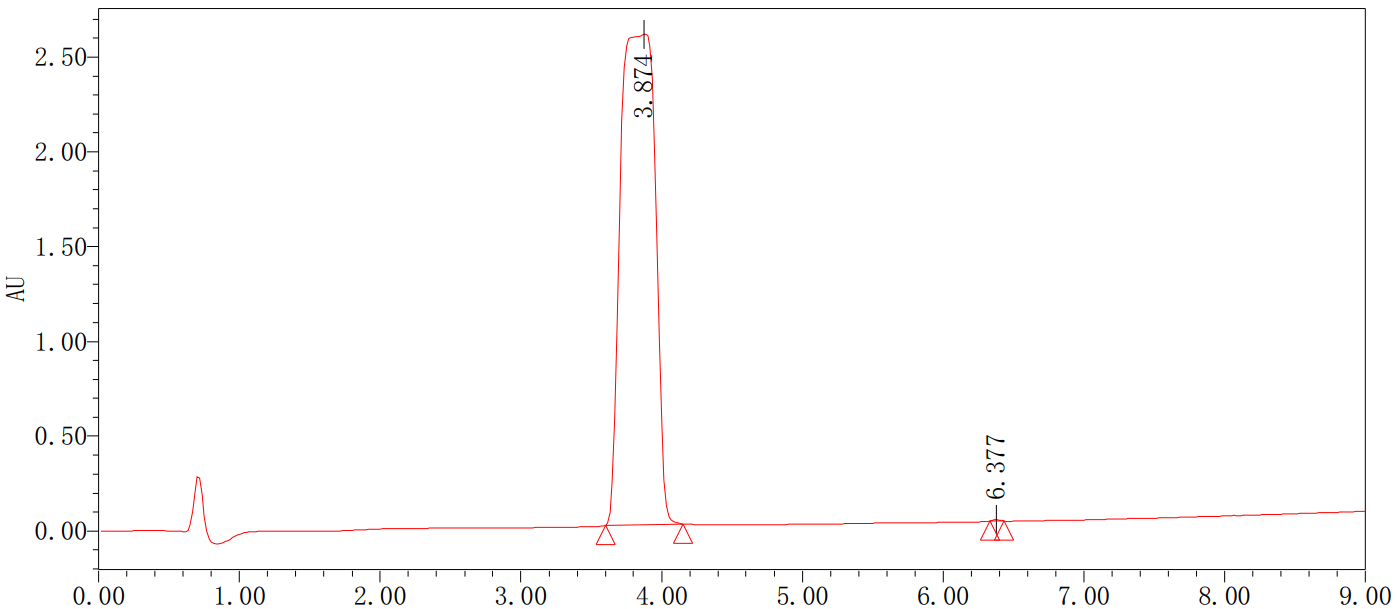


Peak result

|  | Retention time (min) | %Area |
| --- | --- | --- |
| 1 | 3.874 | 99.94 |
| 2 | 6.377 | 0.06 |

^1^H NMR spectrum of compound **4** (500 MHz, DMSO-*d*_6_)


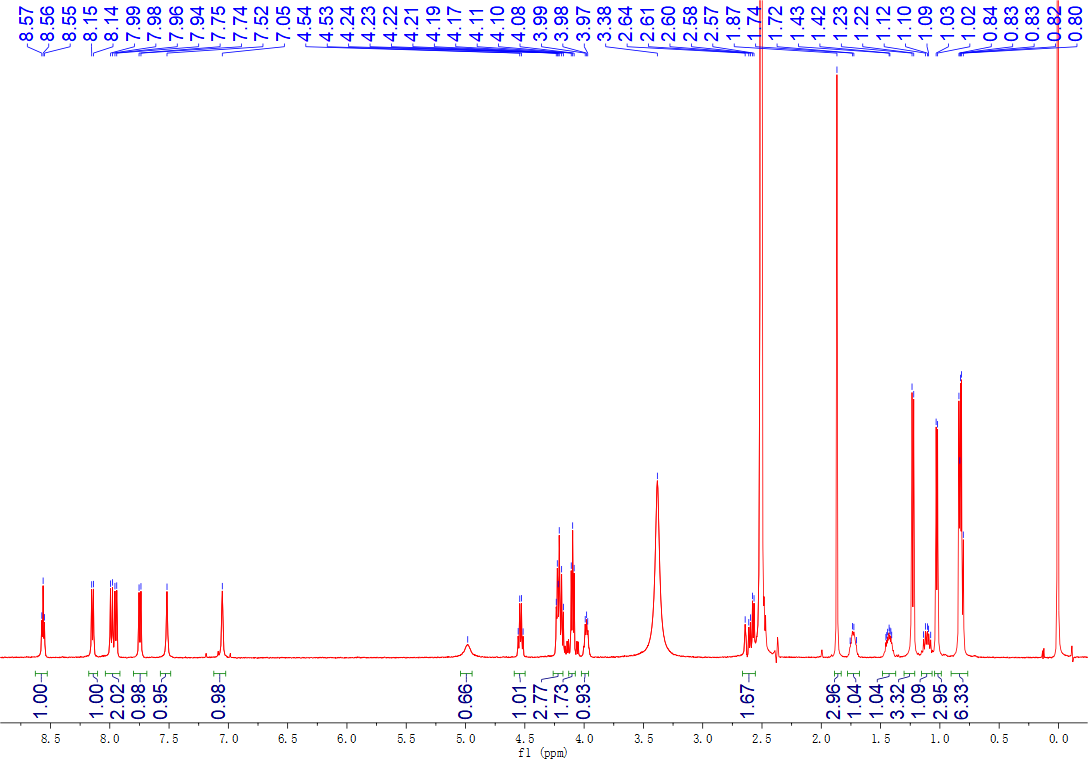


^13^C NMR spectrum of compound **4** (126 MHz, DMSO-*d*_6_)


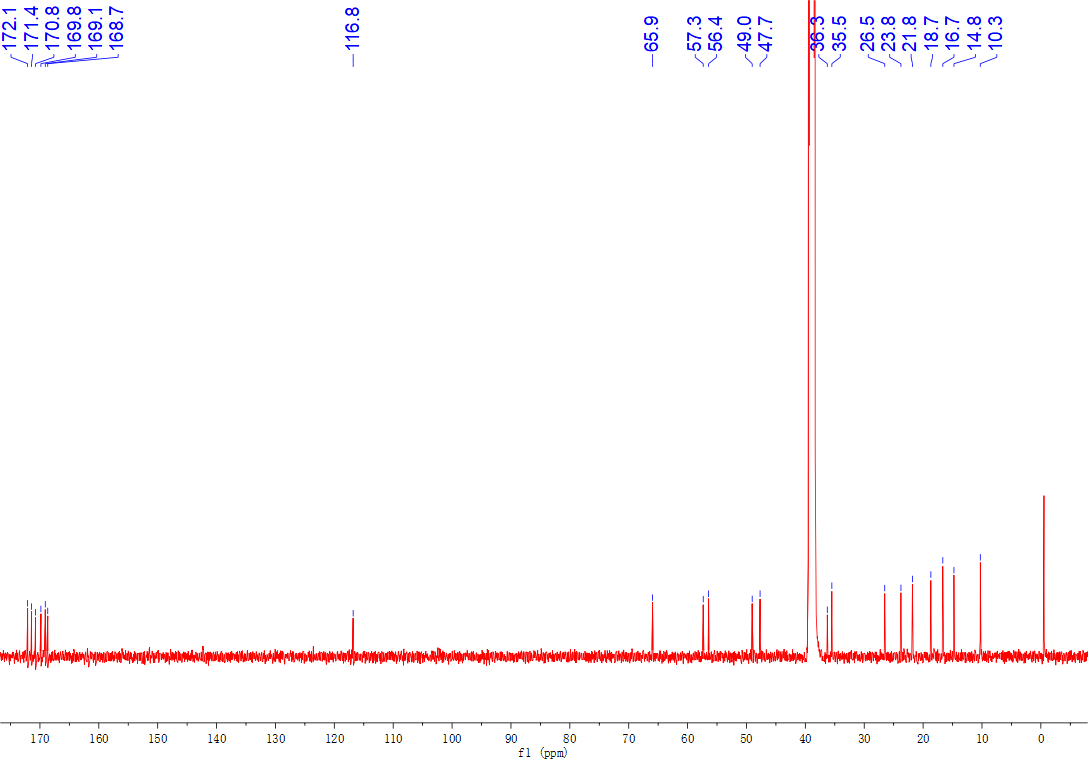


Copy of ESI-HR spectra of compound **4**


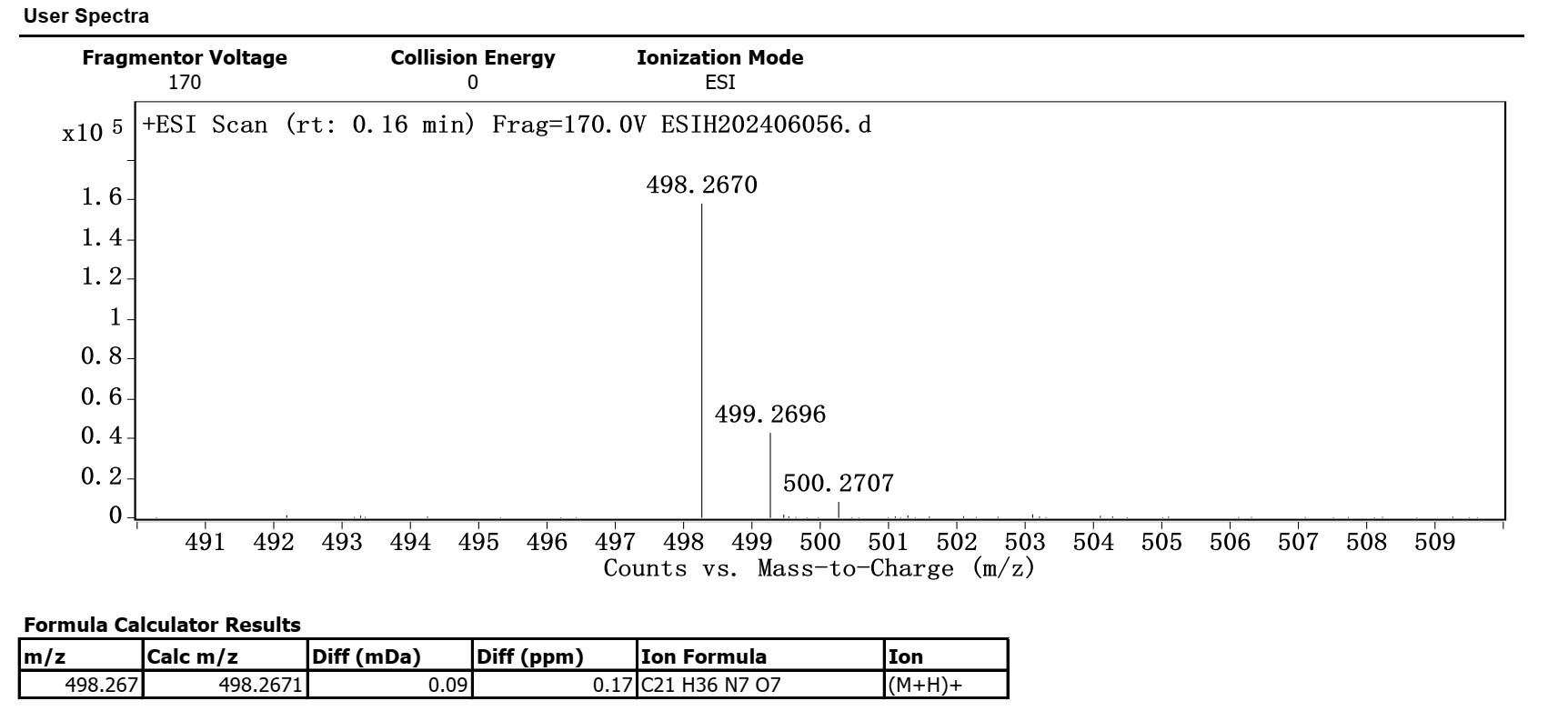


Copy HPLC spectra of compound **4**


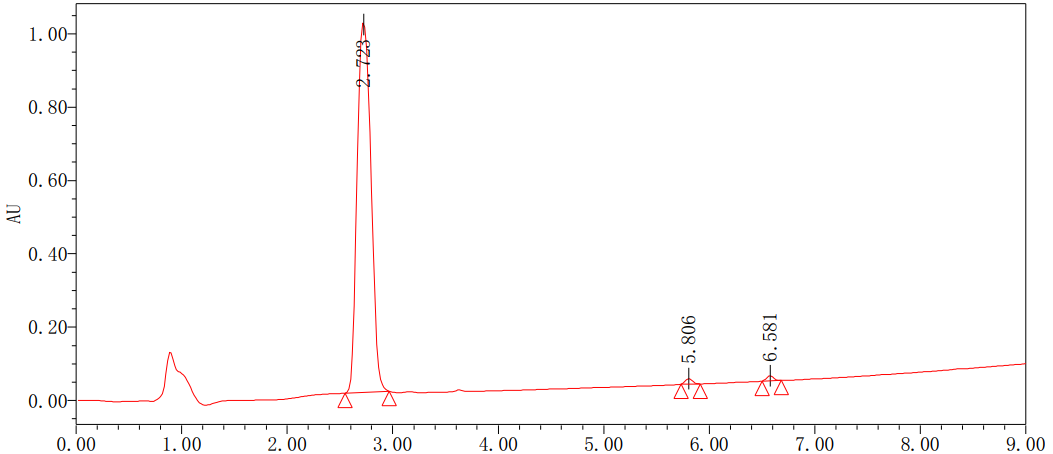


Peak result

|  | Retention time (min) | %Area |
| --- | --- | --- |
| 1 | 2.723 | 98.56 |
| 2 | 5.806 | 0.76 |
| 3 | 6.581 | 0.68 |

^1^H NMR spectrum of compound **5** (500 MHz, DMSO-*d*_6_)


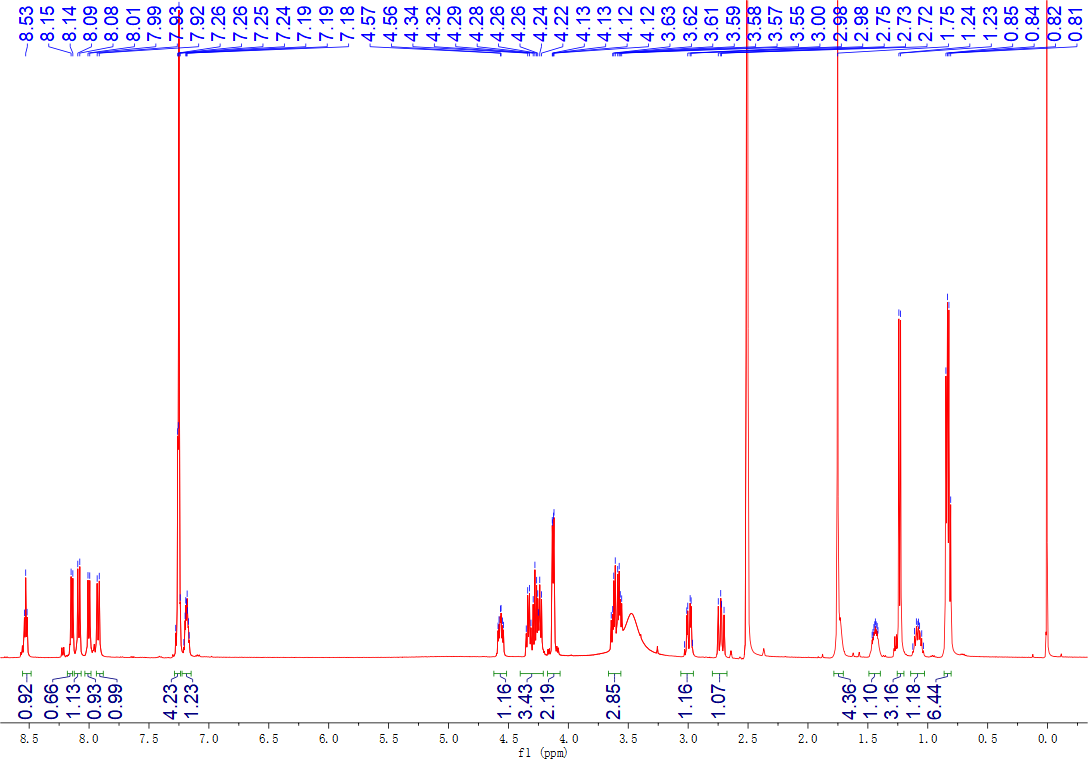


^13^C NMR spectrum of compound **5** (126 MHz, DMSO-*d*_6_)


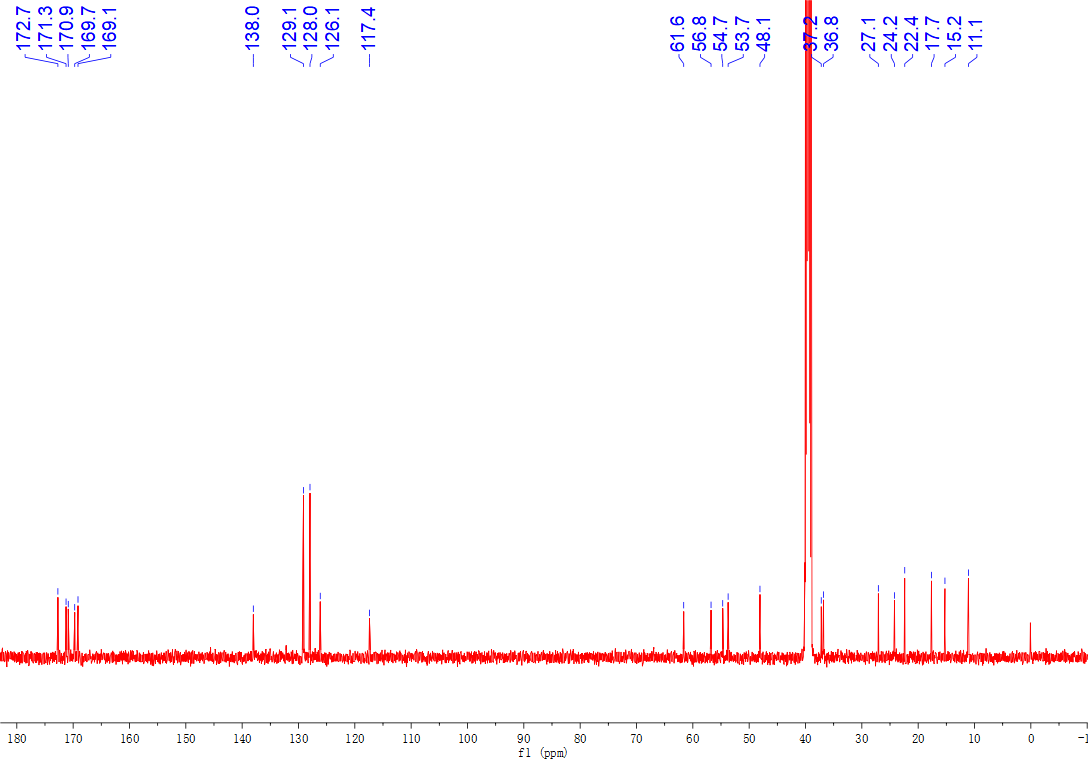


Copy of ESI-HR spectra of compound **5**


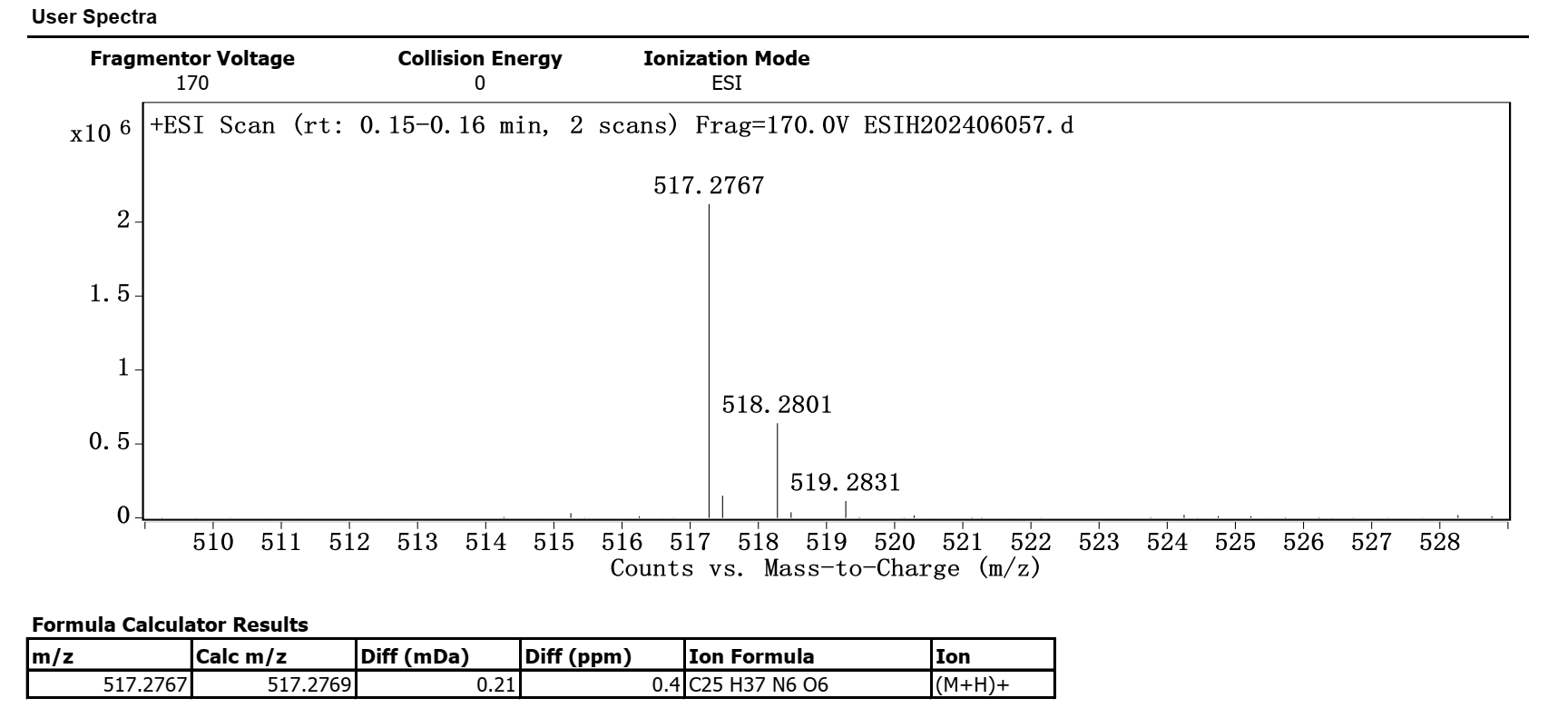


Copy HPLC spectra of compound **5**


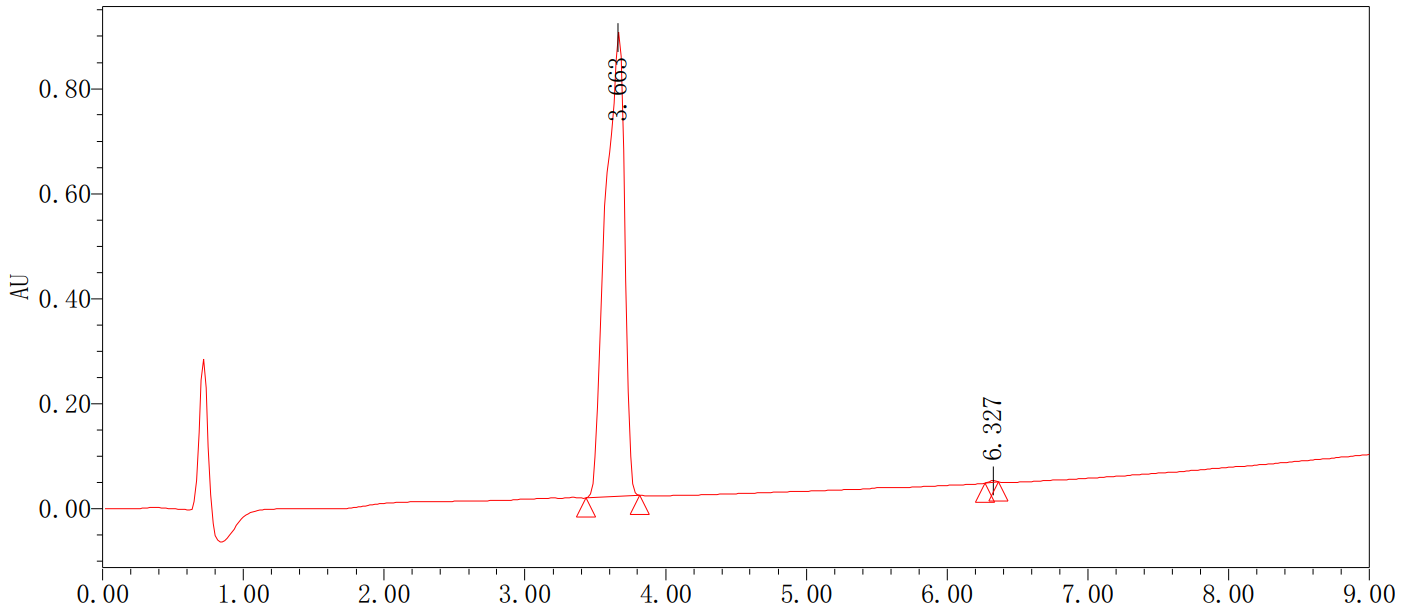


Peak result

|  | Retention time (min) | %Area |
| --- | --- | --- |
| 1 | 3.663 | 99.85 |
| 2 | 6.327 | 0.15 |

^1^H NMR spectrum of compound **6** (500 MHz, DMSO-*d*_6_)


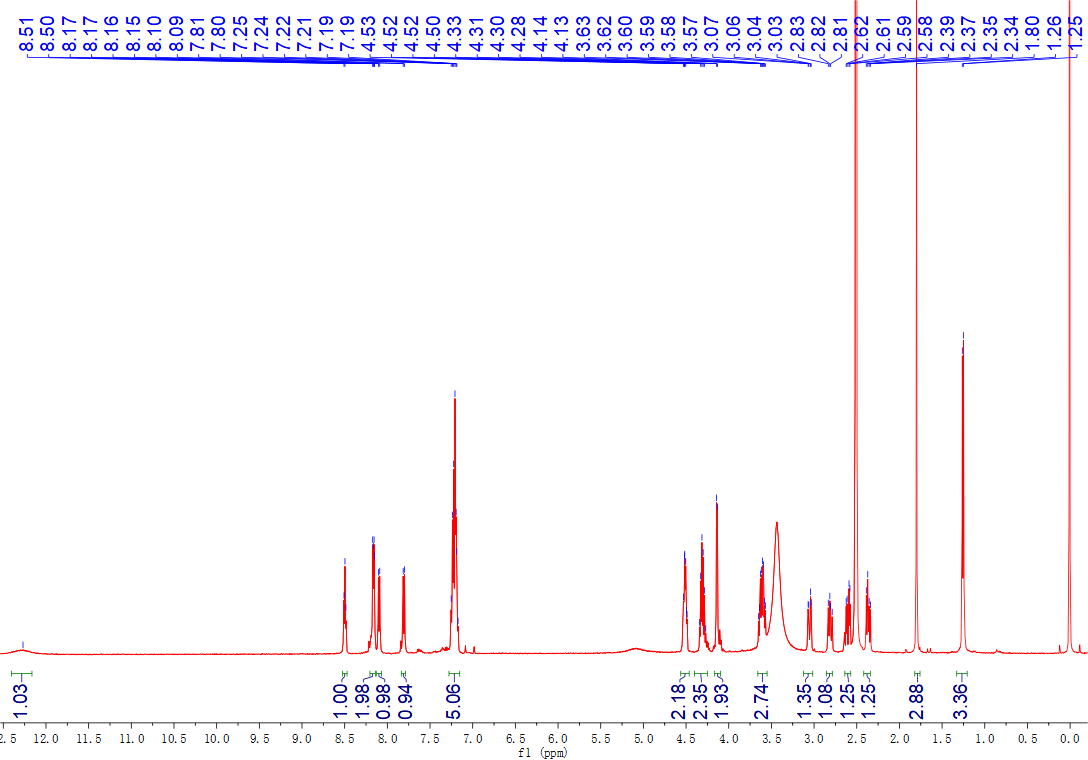

^13^C NMR spectrum of compound **6** (126 MHz, DMSO-*d*_6_)


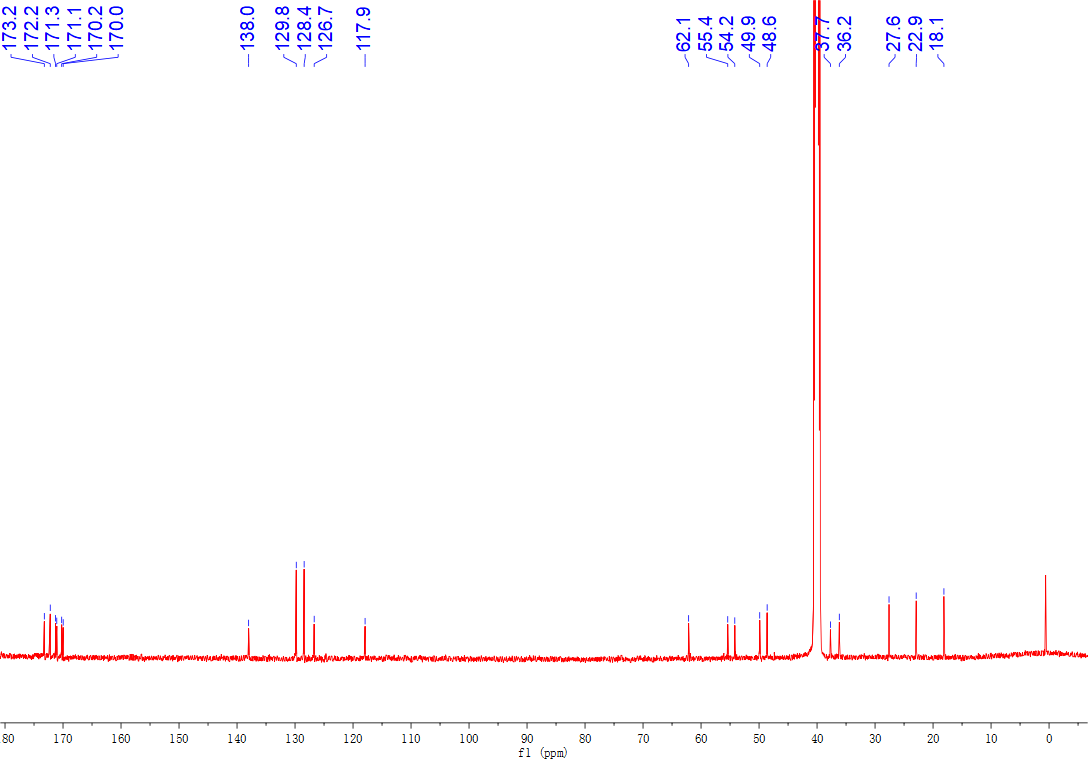


Copy of ESI-HR spectra of compound **6**


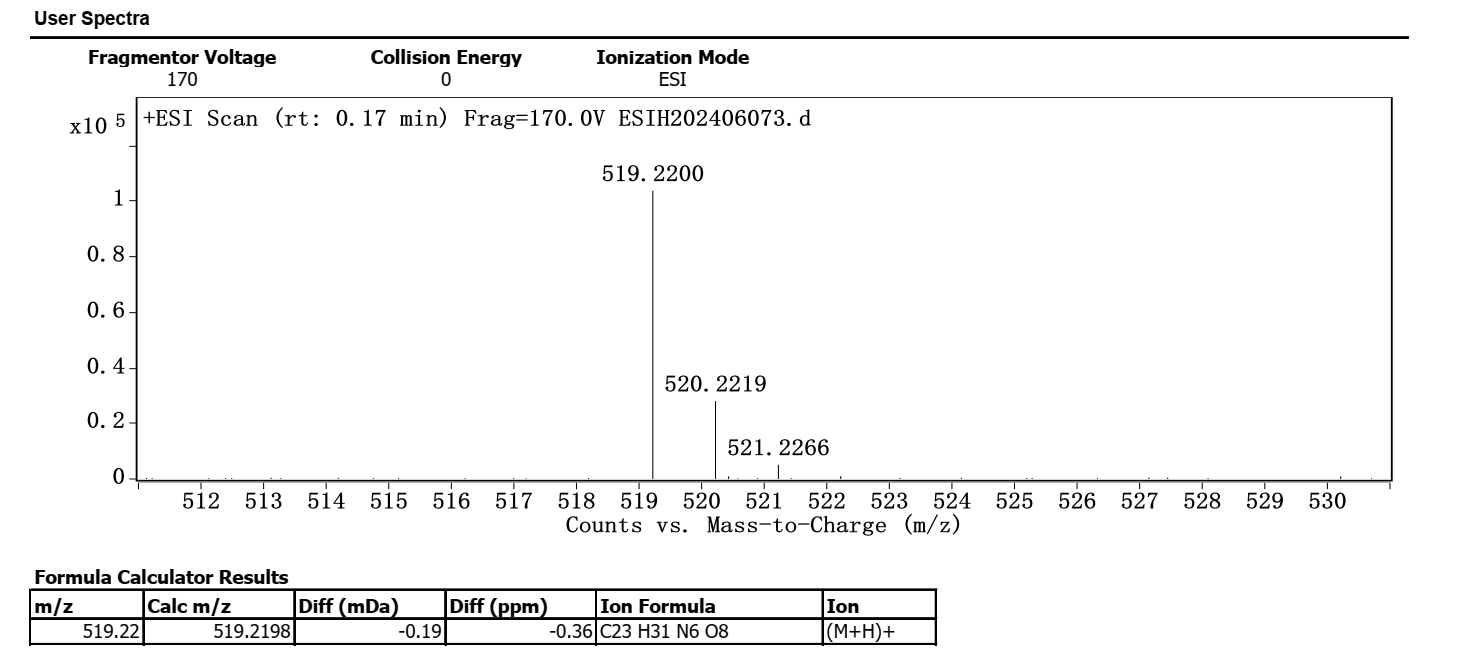


Copy HPLC spectra of compound **6**

**
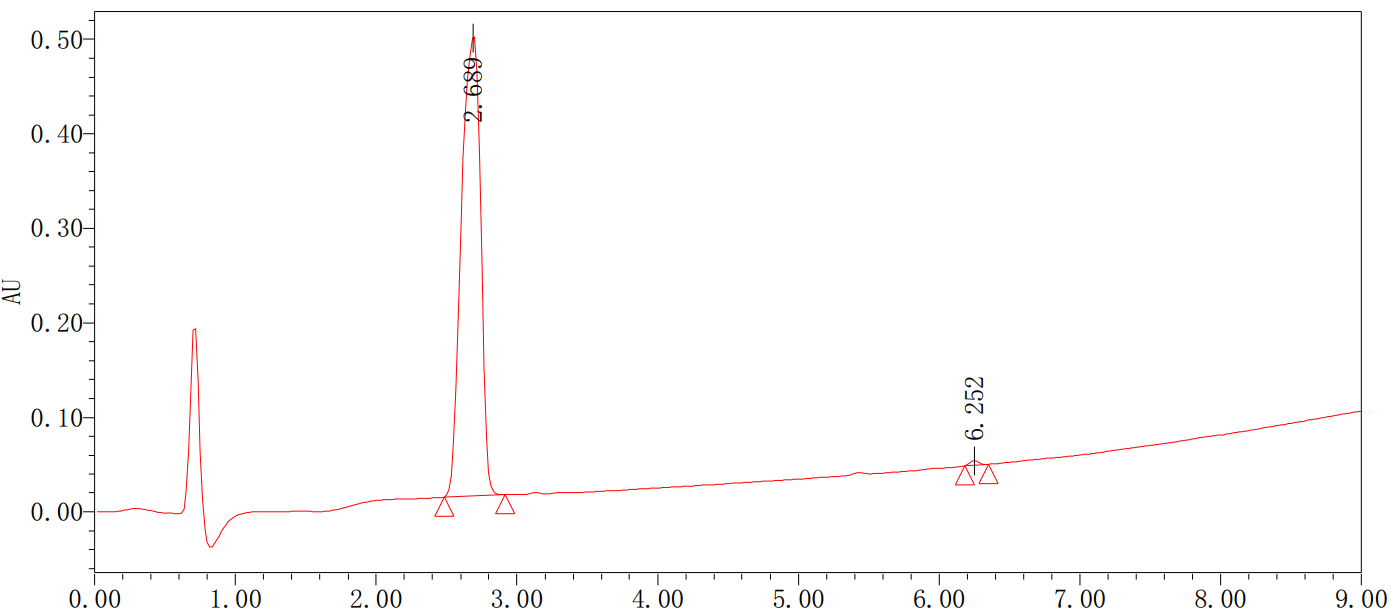
**

Peak result

|  | Retention time (min) | %Area |
| --- | --- | --- |
| 1 | 2.689 | 99.56 |
| 2 | 6.252 | 0.44 |

^1^H NMR spectrum of compound **7** (500 MHz, DMSO-*d*_6_)


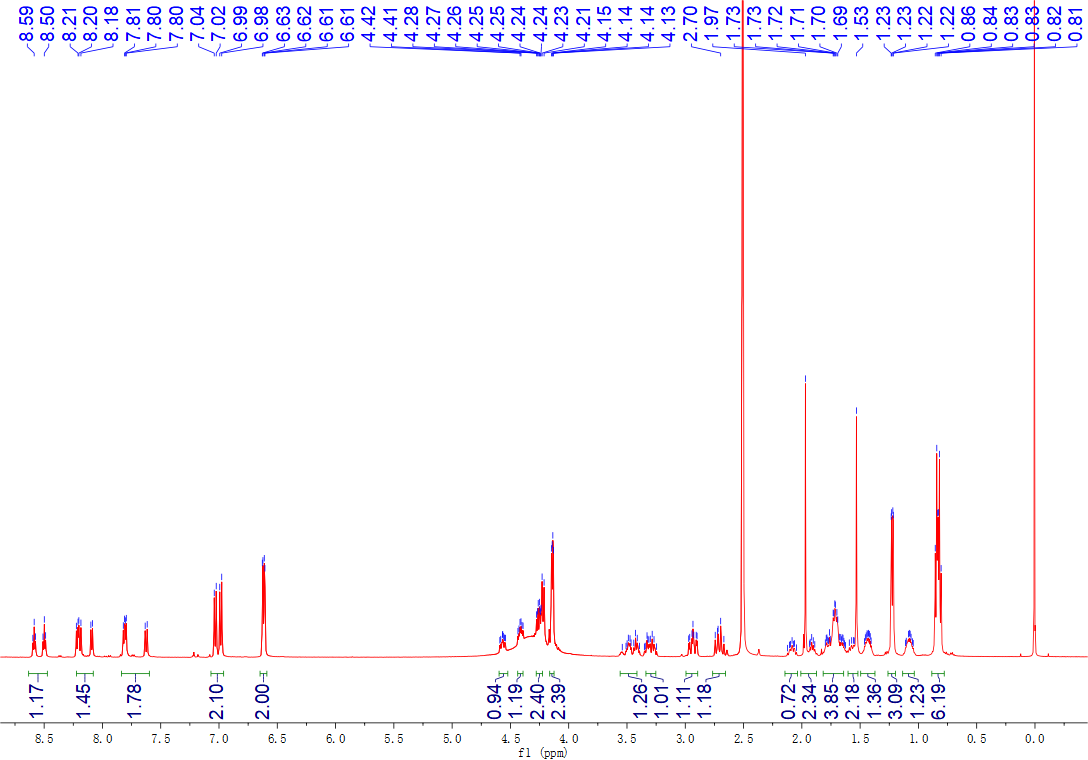


^13^C NMR spectrum of compound **7** (126 MHz, DMSO-*d*_6_)


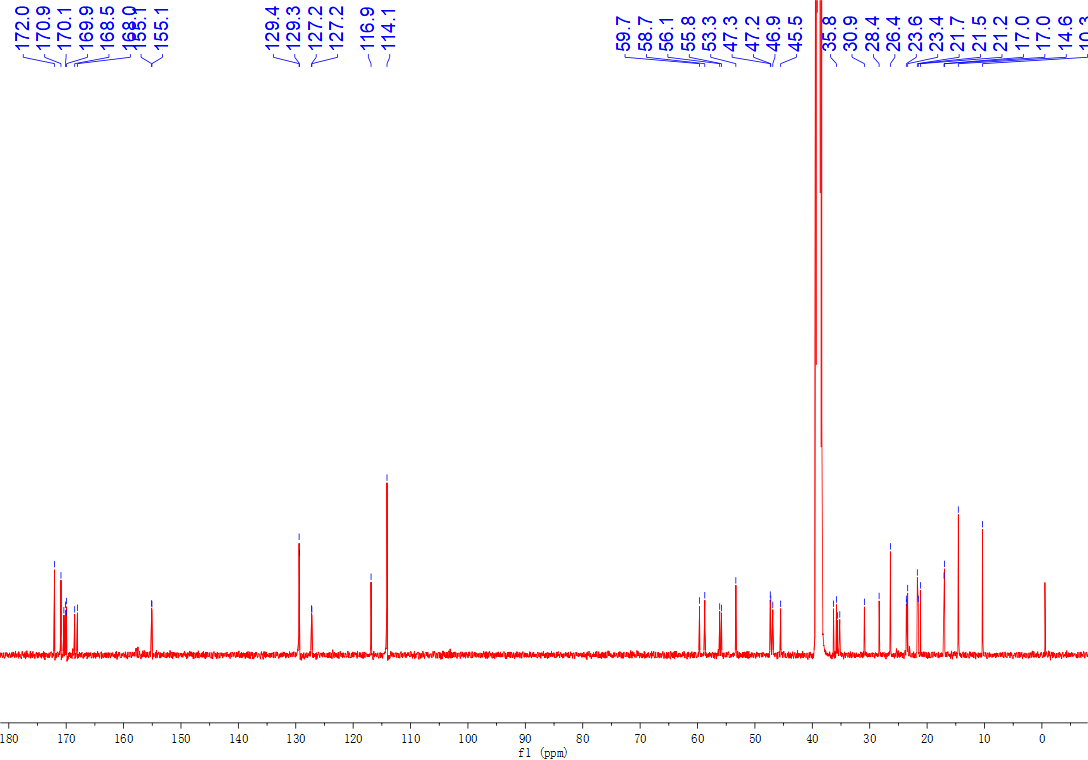


Copy of ESI-HR spectra of compound **7**


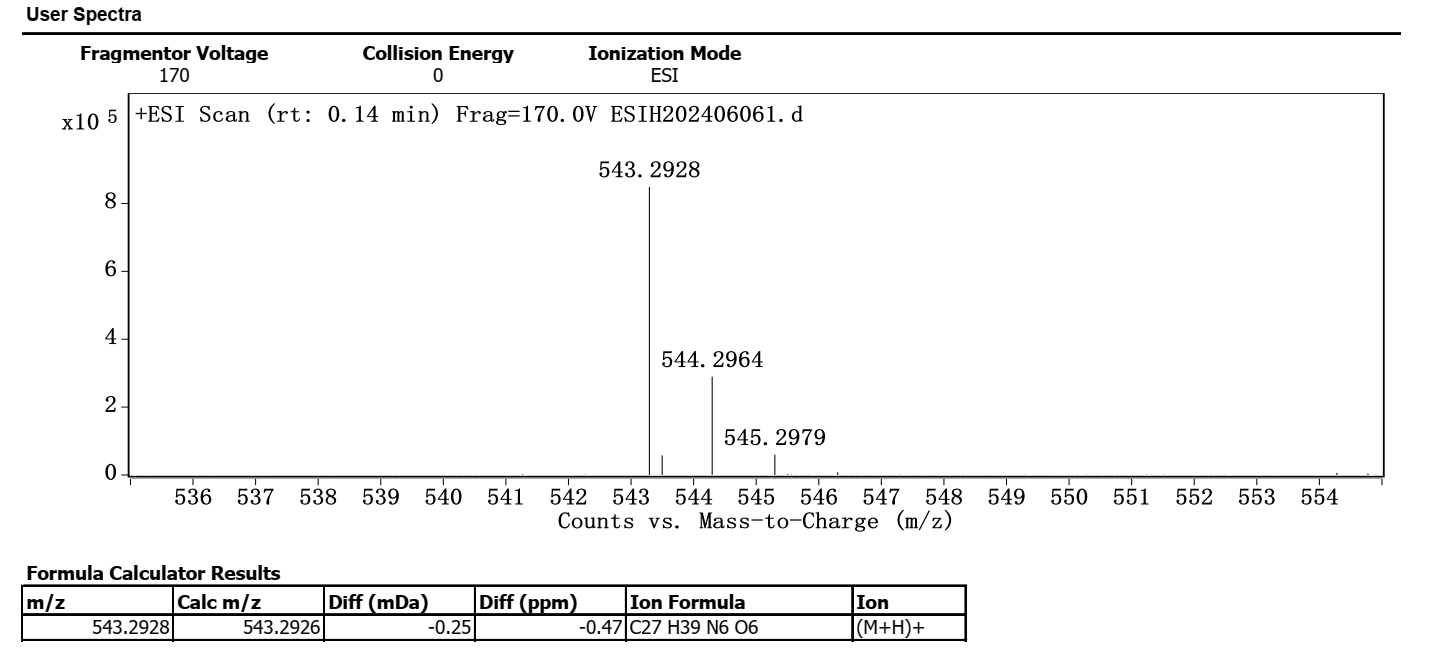


Copy HPLC spectra of compound **7**

**
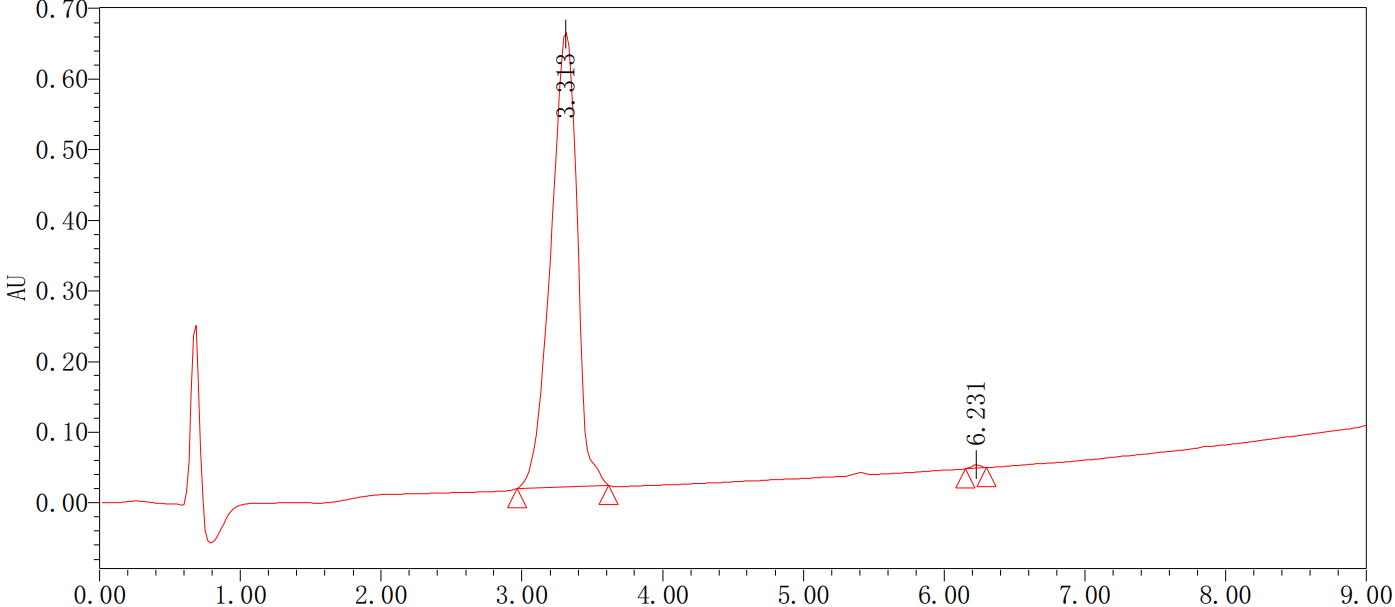
**

Peak result

|  | Retention time (min) | %Area |
| --- | --- | --- |
| 1 | 3.313 | 99.77 |
| 2 | 6.231 | 0.23 |

^1^H NMR spectrum of compound **8** (500 MHz, DMSO-*d*_6_)


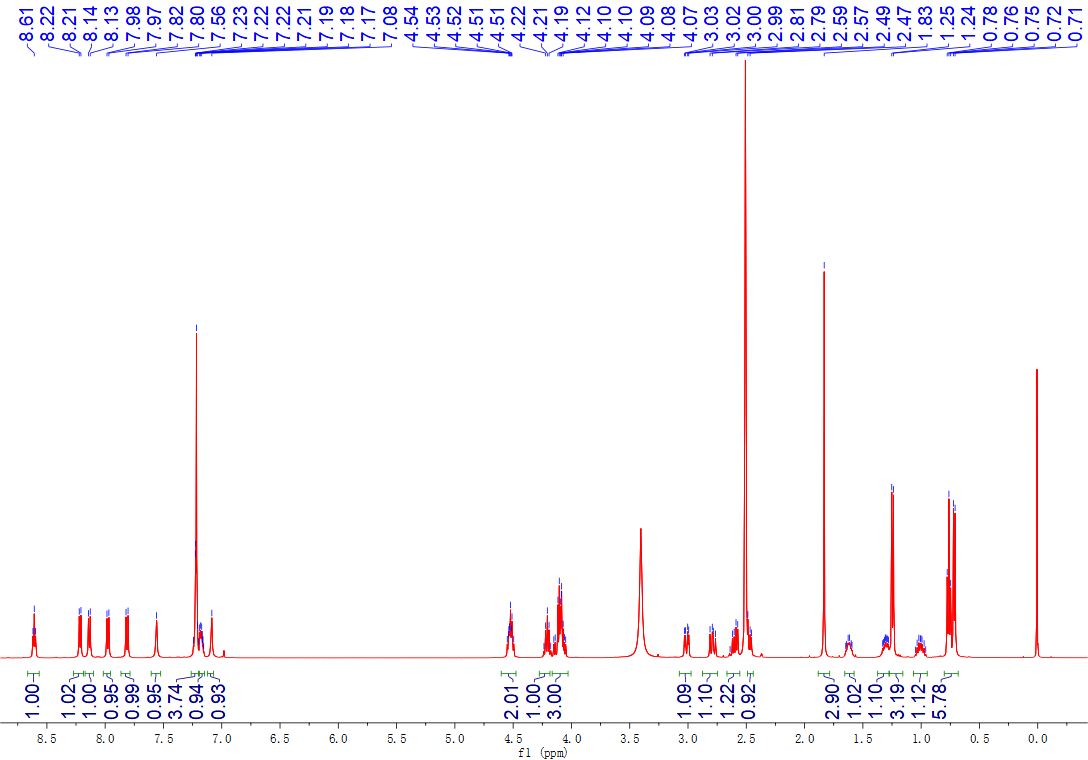


^13^C NMR spectrum of compound **8** (126 MHz, DMSO-*d*_6_)


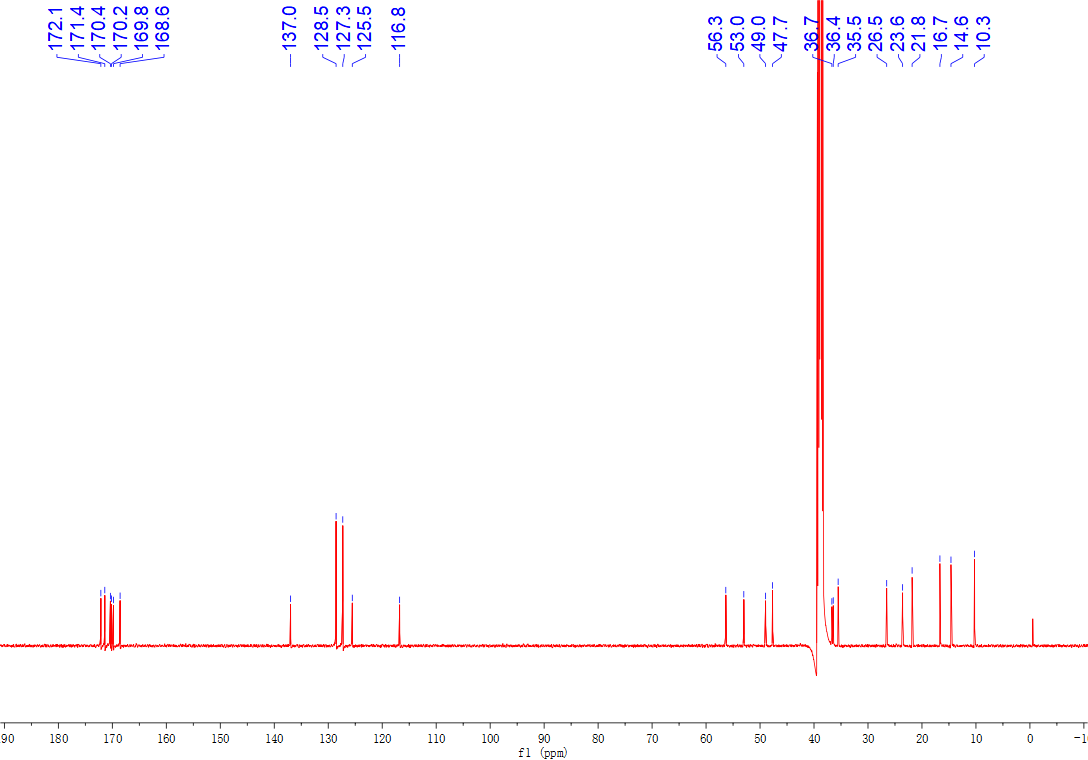


Copy of ESI-HR spectra of compound **8**


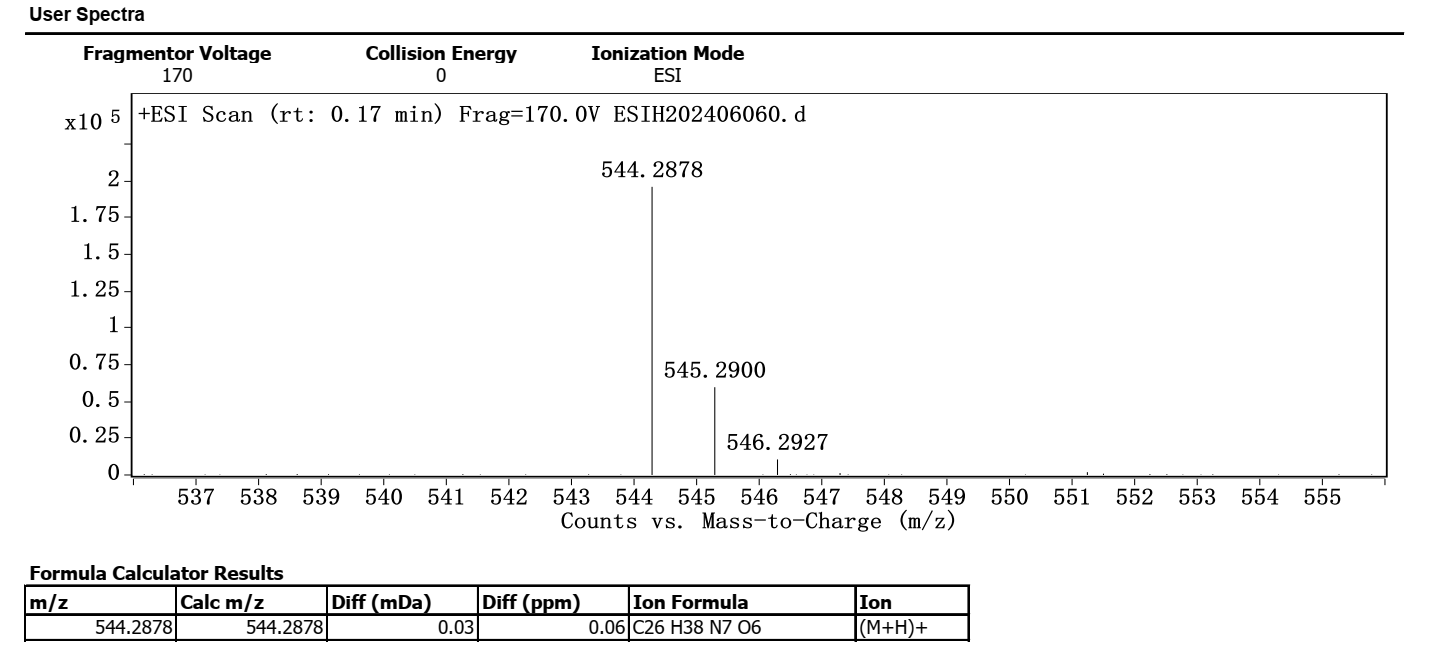


Copy HPLC spectra of compound **8**

**
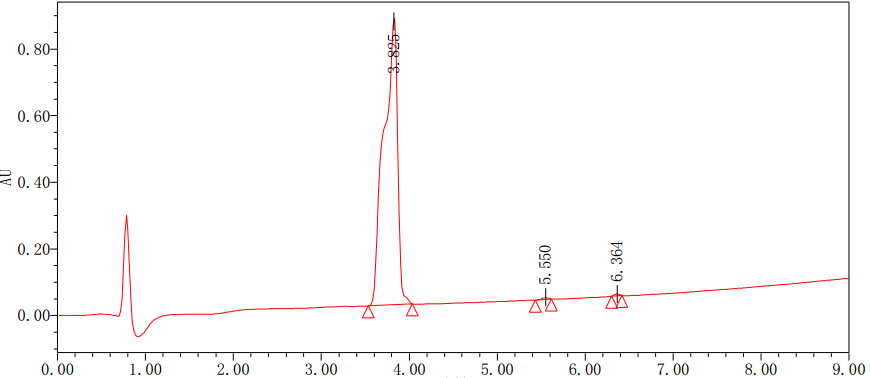
**

Peak result

|  | Retention time (min) | %Area |
| --- | --- | --- |
| 1 | 3.825 | 99.45 |
| 2 | 5.550 | 0.31 |
| 3 | 6.364 | 0.24 |

^1^H NMR spectrum of compound **9** (500 MHz, DMSO-*d*_6_)


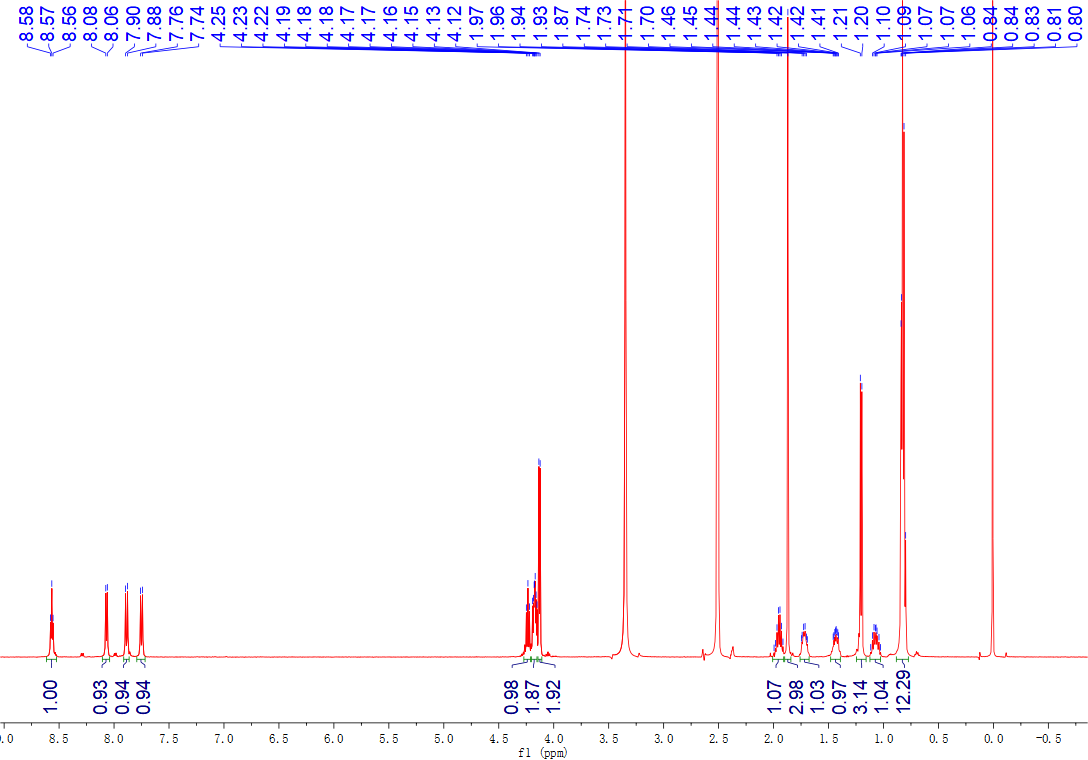


^13^C NMR spectrum of compound **9** (126 MHz, DMSO-*d*_6_)


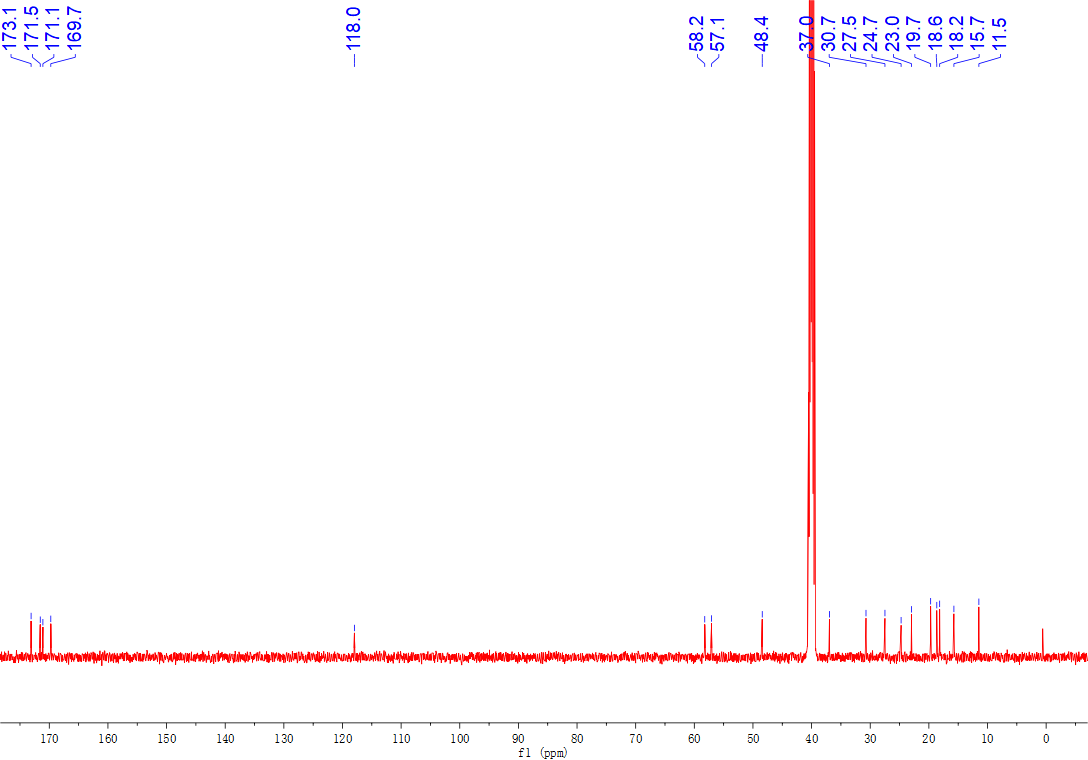


Copy of ESI-HR spectra of compound **9**


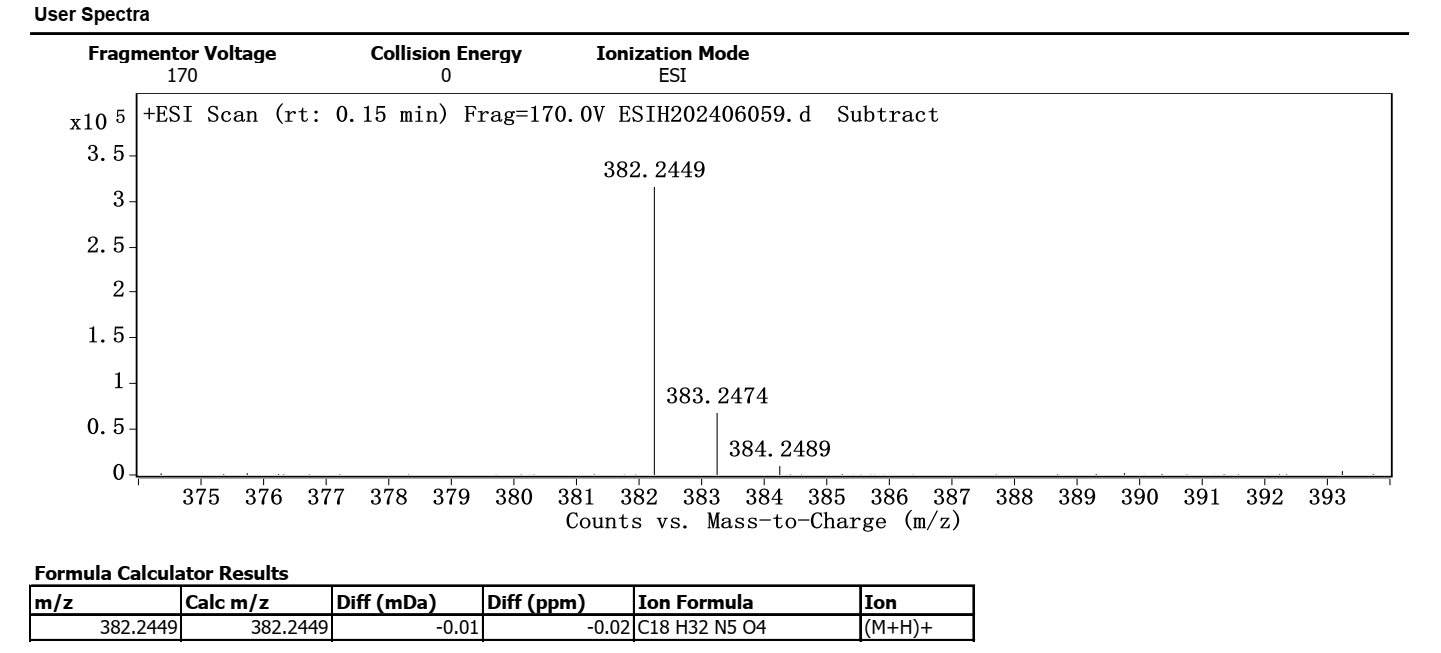


Copy HPLC spectra of compound **9**

**
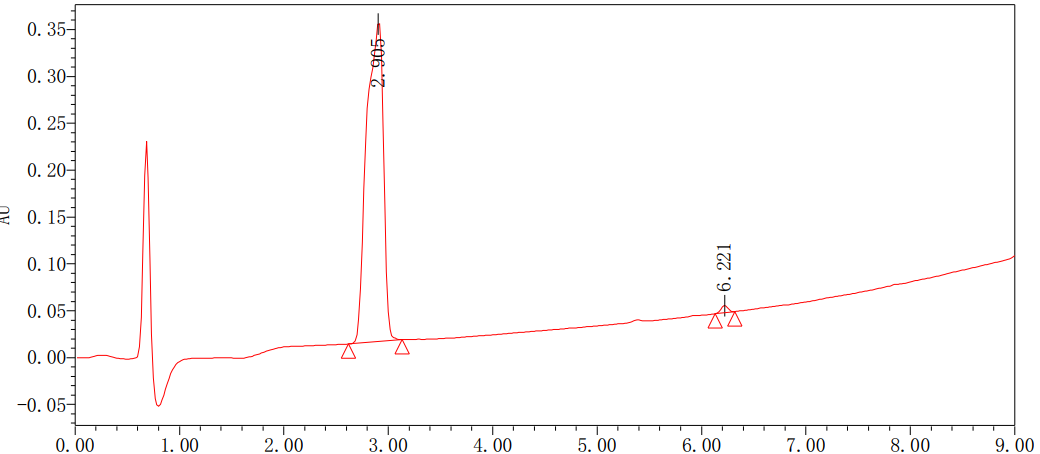
**

Peak result

|  | Retention time (min) | %Area |
| --- | --- | --- |
| 1 | 2.905 | 99.12 |
| 2 | 6.221 | 0.88 |

^1^H NMR spectrum of compound **10** (500 MHz, DMSO-*d*_6_)


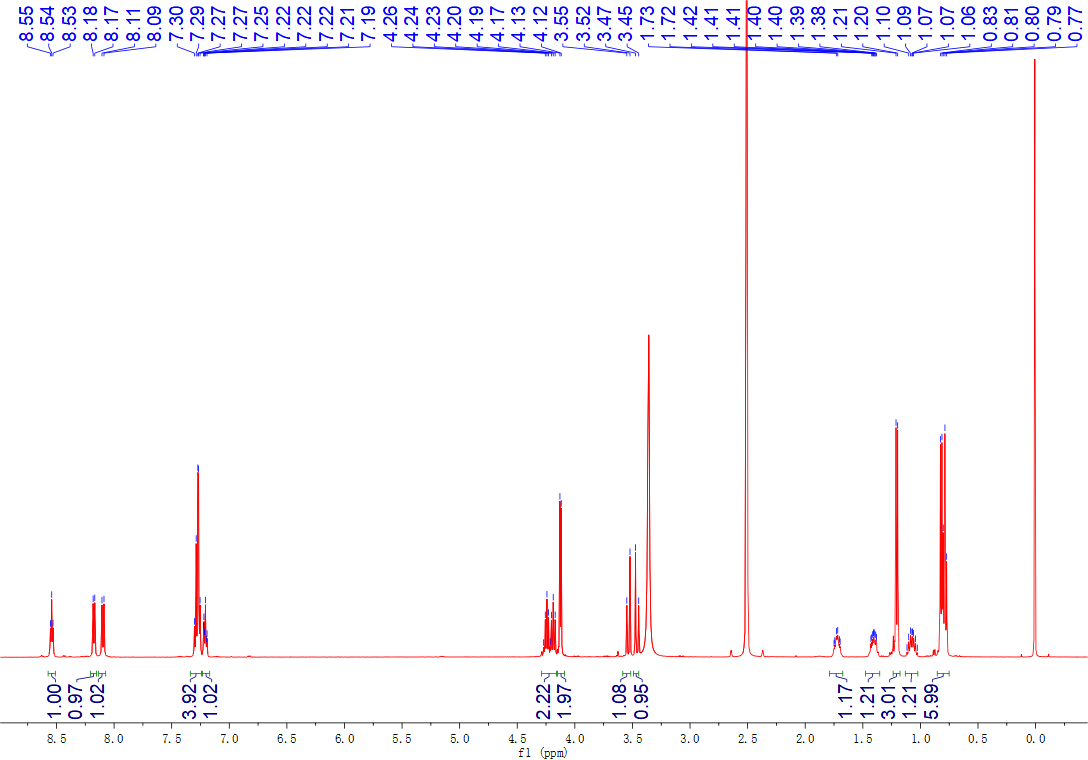


^13^C NMR spectrum of compound **10** (126 MHz, DMSO-*d*_6_)


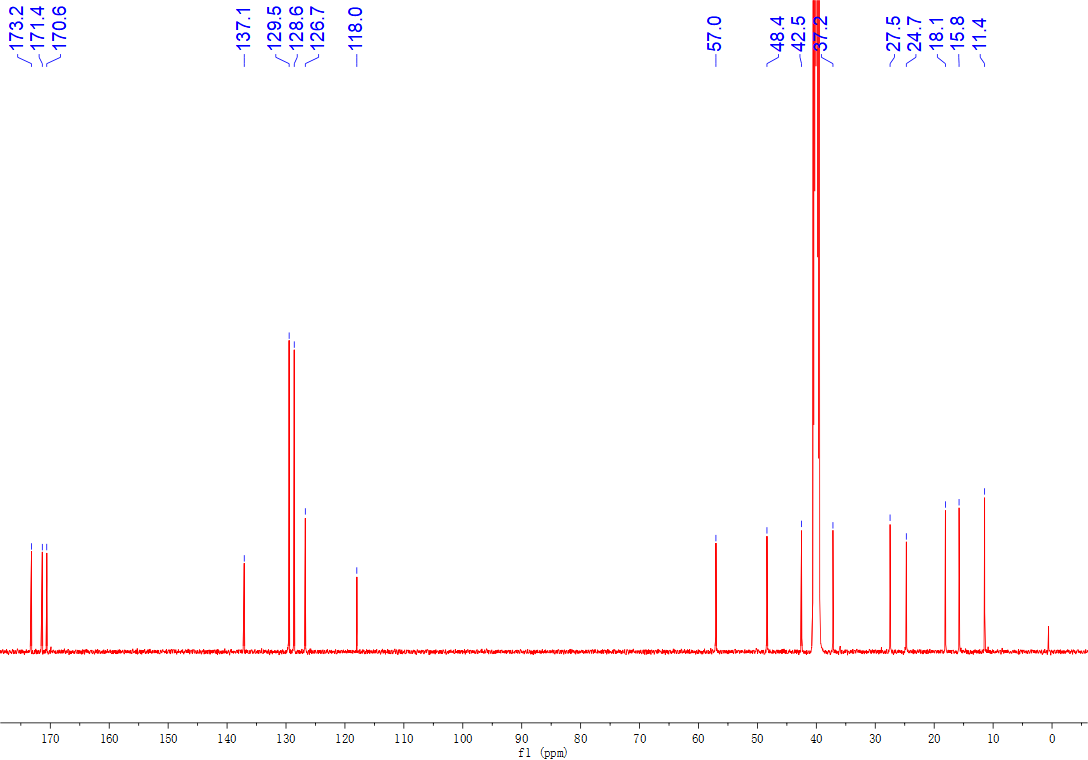


Copy of ESI-HR spectra of compound **10**


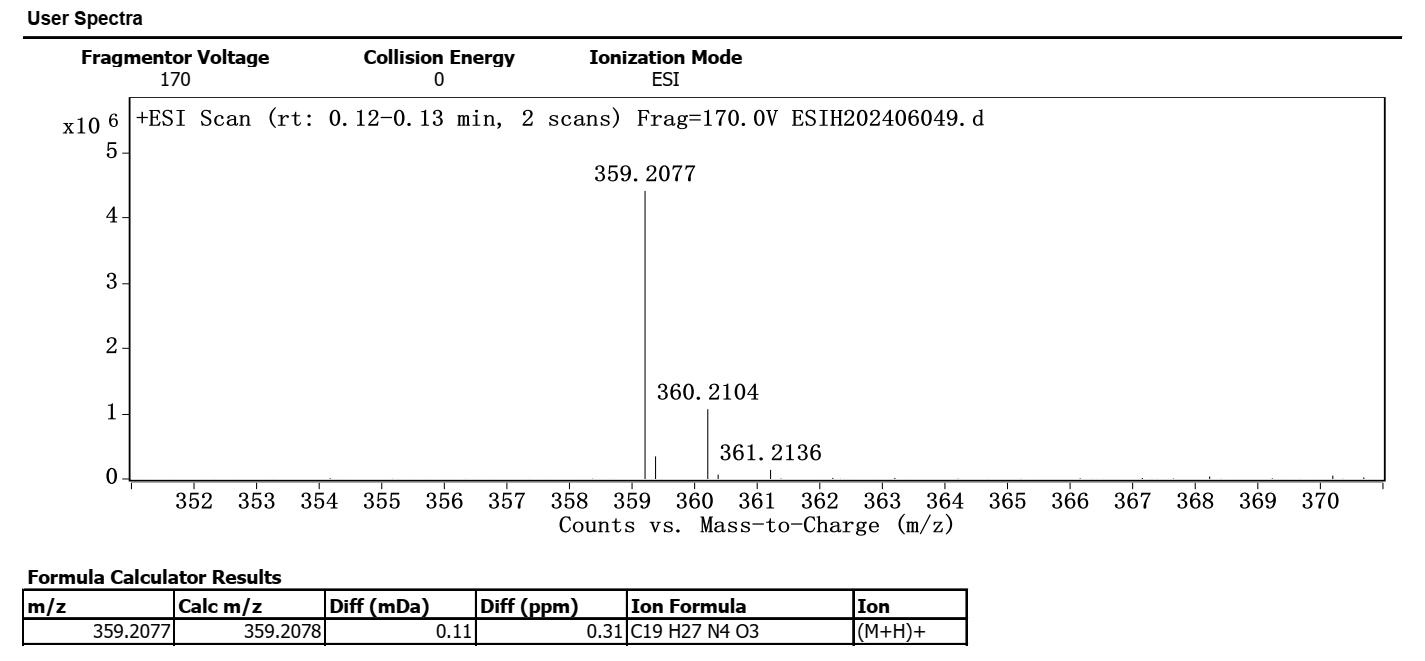


Copy HPLC spectra of compound **10**


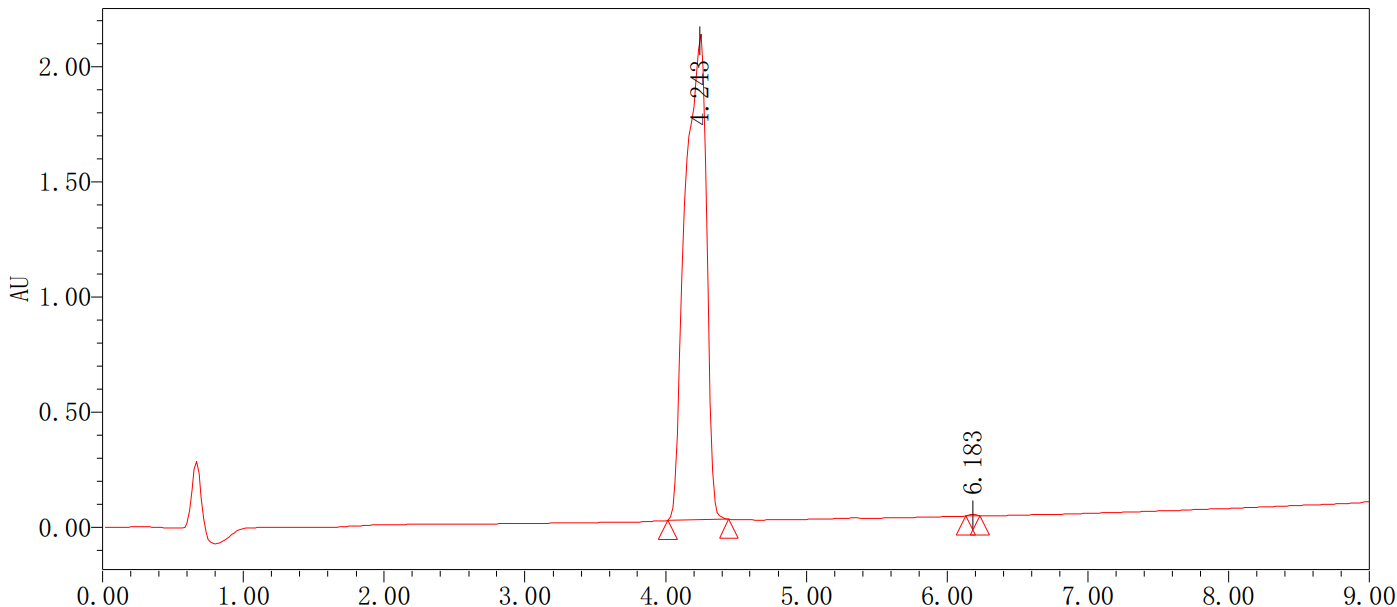


Peak result

|  | Retention time (min) | %Area |
| --- | --- | --- |
| 1 | 4.243 | 99.92 |
| 2 | 6.183 | 0.08 |

^1^H NMR spectrum of compound **11** (500 MHz, DMSO-*d*_6_)


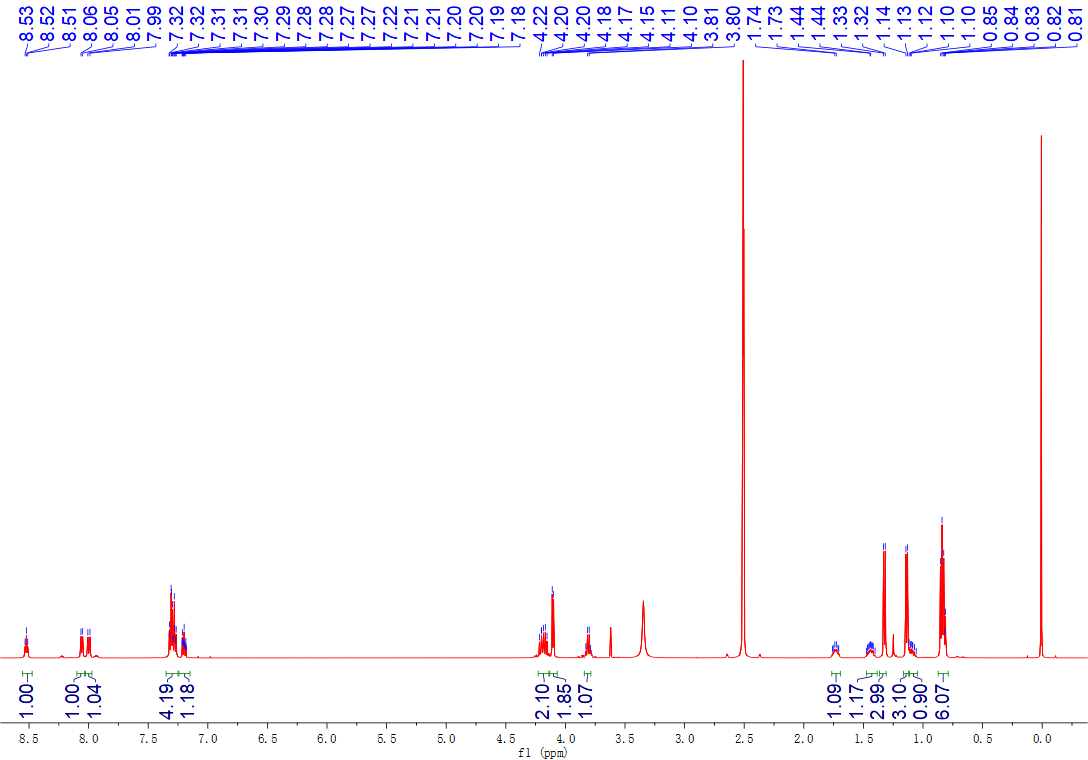


^13^C NMR spectrum of compound **11** (126 MHz, DMSO-*d*_6_)


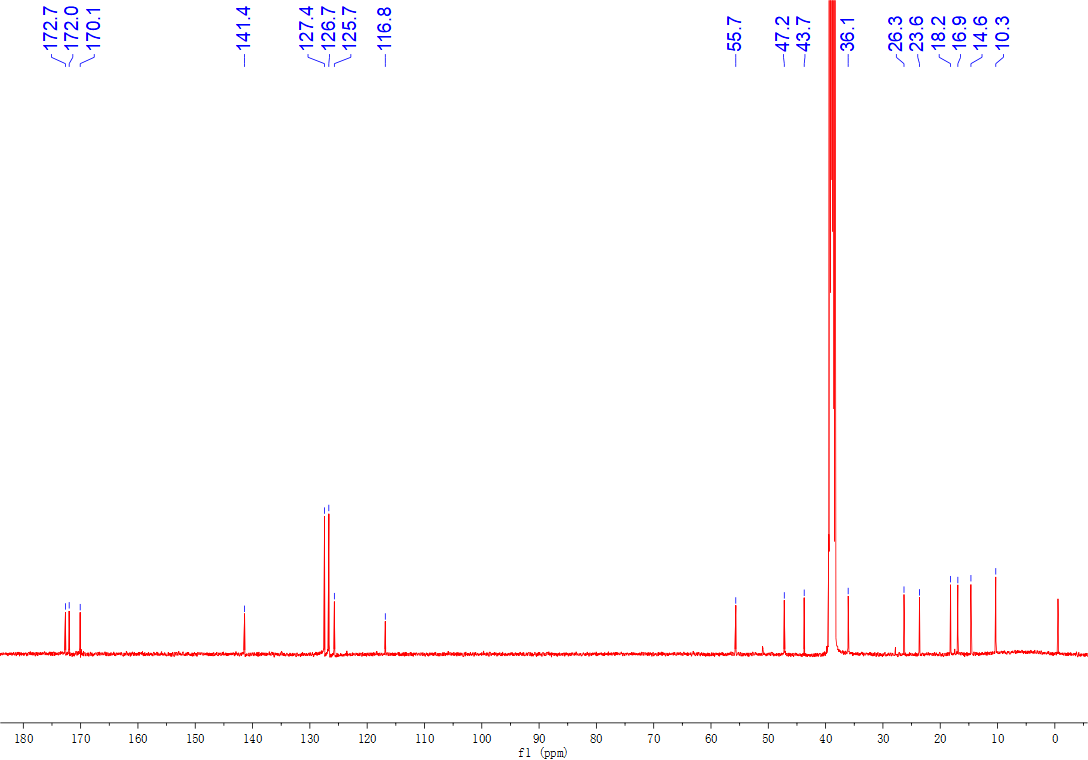


Copy of ESI-HR spectra of compound **11**


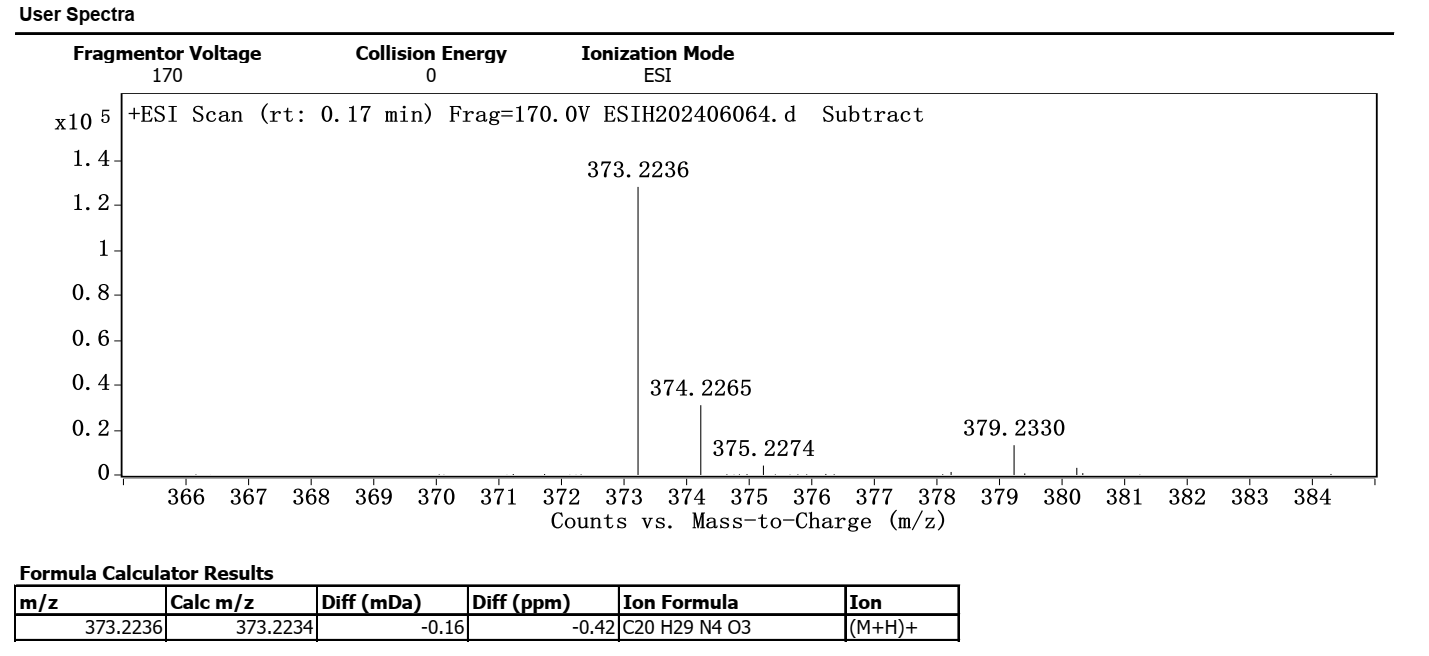


Copy HPLC spectra of compound **11**


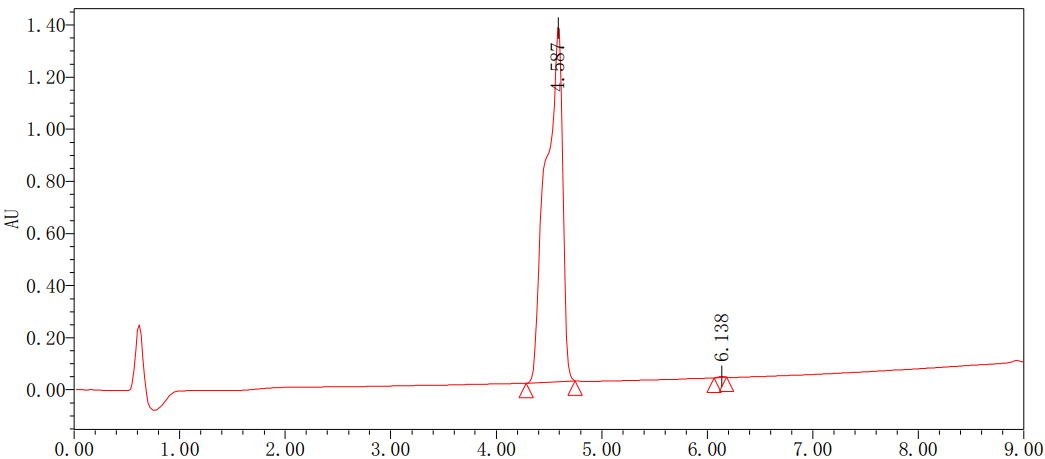


Peak result

|  | Retention time (min) | %Area |
| --- | --- | --- |
| 1 | 4.587 | 99.90 |
| 2 | 6.138 | 0.10 |

# References

[1] F. Yu, Q. Wang, M. Li, H. Zhou, K. Liu, K. Zhang, Z. Wang, Q. Xu, C. Xu, Q. Pan, J. He, *J. Appl. Crystallogr.* **2019**, *52*, 472-477.

[2] W.-Z. Zhang, J.-C. Tang, S.-S. Wang, Z.-J. Wang, W.-M. Qin, J.-H. He, *Nucl. Sci. Tech.* **2019**, *30*, 170.

[3] W. Minor, M. Cymborowski, Z. Otwinowski, M. Chruszcz, *Acta Crystallogr. D* **2006**, *62*, 859–866.

[4] A. J. McCoy, R. W. Grosse-Kunstleve, P. D. Adams, M. D. Winn, L. C. Storoni, R. J. Read, *J. Appl. Crystallogr.* **2007**, *40*, 658–674.

[5] P. Emsley, K. Cowtan, *Acta Crystallogr. D* **2004**, *60*, 2126–2132.

[6] P. D. Adams, R. W. Grosse-Kunstleve, L. W. Hung, T. R. Ioerger, A. J. McCoy, N. W. Moriarty, R. J. Read, J. C. Sacchettini, N. K. Sauter, T. C. Terwilliger, *Acta Crystallogr. D* **2002**, *58*, 1948–1954.

[7] J. Abramson, J. Adler, J. Dunger, R. Evans, T. Green, A. Pritzel, O. Ronneberger, L. Willmore, A. J. Ballard, J. Bambrick, S. W. Bodenstein, D. A. Evans, C. C. Hung, M. O'Neill, D. Reiman, K. Tunyasuvunakool, Z. Wu, A. Žemgulytė, E. Arvaniti, C. Beattie, O. Bertolli, A. Bridgland, A. Cherepanov, M. Congreve, A. I. Cowen-Rivers, A. Cowie, M. Figurnov, F. B. Fuchs, H. Gladman, R. Jain, Y. A. Khan, C. M. R. Low, K. Perlin, A. Potapenko, P. Savy, S. Singh, A. Stecula, A. Thillaisundaram, C. Tong, S. Yakneen, E. D. Zhong, M. Zielinski, A. Žídek, V. Bapst, P. Kohli, M. Jaderberg, D. Hassabis, J. M. Jumper, *Nature* **2024**, *630*, 493-500.

[8] G. M. Sastry, M. Adzhigirey, T. Day, R. Annabhimoju, W. Sherman, *J. Comput. Aided Mol. Des.* **2013**, *27*, 221-234.

[9] E. Harder, W. Damm, J. Maple, C. Wu, M. Reboul, J. Y. Xiang, L. Wang, D. Lupyan, M. K. Dahlgren, J. L. Knight, J. W. Kaus, D. S. Cerutti, G. Krilov, W. L. Jorgensen, R. Abel, R. A. Friesner, *J. Chem. Theory Comput.* **2016**, *12*, 281-296.

[10] K. Zhu, K. W. Borrelli, J. R. Greenwood, T. Day, R. Abel, R. S. Farid, E. Harder, *J. Chem. Inf. Model.* **2014**, *54*, 1932-1940.

[11] C. Wang, P. Bradley, D. Baker, *J. Mol. Biol.* **2007**, *373*, 503-519.

[12] D. J. Mandell, E. A. Coutsias, T. Kortemme, *Nat. Methods* **2009**, *6*, 551-552.

[13] S. Jo, T. Kim, V. G. Iyer, W. Im, *J. Comput. Chem.* **2008**, *29*, 1859-1865.

[14] Case, H.M. Aktulga, K. Belfon, I.Y. Ben-Shalom, J.T. Berryman, S.R. Brozell, D.S. Cerutti, T.E. Cheatham, III, G.A. Cisneros, V.W.D. Cruzeiro, T.A. Darden, R.E. Duke, G. Giambasu, M.K. Gilson, H. Gohlke, A.W. Goetz, R. Harris, S. Izadi, S.A. Izmailov, K. Kasavajhala, M.C. Kaymak, E. King, A. Ko valenko, T. Kurtzman, T.S. Lee, S. LeGrand, P. Li, C. Lin, J. Liu, T. Luchko, R. Luo, M. Machado, V. Man, M. Manathunga, K.M. Merz, Y. Miao, O. Mikhailovskii, G. Monard, H. Nguyen, K.A. O’Hearn, A. Onufriev, F. Pan, S. Pantano, R. Qi, A. Rahnamoun, D.R. Roe, A. Roitberg, C. Sagui, S. Schott-Verdugo, A. Shajan, J. Shen, C.L. Simmerling, N.R. Skrynnikov, J. Smith, J. Swails, R.C. Walker, J. Wang, J. Wang, H. Wei, R.M. Wolf, X. Wu, Y. Xiong, Y. Xue, D.M. York, S. Zhao, and P.A. Kollman, Amber 2022, University of California, San Francisco (America), 2022.

[15] J. A. Maier, C. Martinez, K. Kasavajhala, L. Wickstrom, K. E. Hauser, C. Simmerling, *J. Chem. Theory Comput.* **2015**, *11*, 3696-3713.

[16] W. L. Jorgensen, J. Chandrasekhar, J. D. Madura, R. W. Impey, M. L. Klein, *J. Chem. Phys.* **1983**, *79*, 926-935.

[17] J. P. Ryckaert, G. Ciccotti, H. J. C. Berendsen, *J. Comput. Phys.* **1977**, *23*, 327-341.

[18] T. Darden, D. York, L. Pedersen, *J. Chem. Phys.* **1993**, *98*, 10089-10092.

[19] Y. L. Miao, V. A. Feher, J. A. McCammon, *J. Chem. Theory Comput.* **2015**, *11*, 3584-3595.

[20] P. Ayaz, A. Lyczek, Y. Paung, V. R. Mingione, R. E. Iacob, P. W. de Waal, J. R. Engen, M. A. Seeliger, Y. Shan, D. E. Shaw, *Nat. Commun.* **2023**, *14*, 1885.

[21] R. C. Walker, M. F. Crowley, D. A. Case, *J. Comput. Chem.* **2008**, *29*, 1019-1031.

[22] G. D. Seabra, R. C. Walker, M. Elstner, D. A. Case, A. E. Roitberg, *J. Phys. Chem. A* **2007**, *111*, 5655-5664.

[23] B. Isralewitz, M. Gao, K. Schulten, *Curr. Opin. Struct. Biol.* **2001**, *11*, 224-230.

[24] G. M. Torrie, J. P. Valleau, *J. Comput. Phys.* **1977**, *23*, 187-199.

[25] S. Kumar, D. Bouzida, R. H. Swendsen, P. A. Kollman, J. M. Rosenberg, *J. Comput. Chem.* **1992**, *13*, 1011-1021.
